# A method for investigating spatiotemporal growth patterns at cell and tissue levels during C-looping in the embryonic chick heart

**DOI:** 10.1016/j.isci.2022.104600

**Published:** 2022-06-14

**Authors:** Nazanin Ebrahimi, Mahyar Osanlouy, Chris P. Bradley, M. Fabiana Kubke, Dane A. Gerneke, Peter J. Hunter

**Affiliations:** 1 University of Auckland, Auckland Bioengineering Institute, Auckland 1010, New Zealand; 2 University of Auckland, Anatomy and Medical Imaging, Auckland 1010, New Zealand

**Keywords:** Biological sciences, Developmental biology, Biological sciences research methodologies

## Abstract

We developed a workflow using multi-scale and multi-disciplinary experimental and computational approaches to analyze C-looping (the first phase of cardiac looping) of the chick across four developing hearts. We provide the first 3D datasets for the C-looping heart with cell to organism level information, including datasets of heart images and segmented myocardial cells within the heart. We used these datasets to investigate, as a proof-of-concept, the differential spatiotemporal patterns of growth at both the cellular and tissue levels, and demonstrate how geometrical changes of C-looping at the tissue level are linked to growth features at the cellular level. Our methodological pipeline provides preliminary results for qualitative and quantitative evidence of various cellular and tissue features as potential candidates regarding the mechanism of C-looping. This pipeline can be used and extended in future studies to include larger specimen samples for detailed analyses of, and potentially new insights into, cardiac C-looping.

## Highlights

• A hybrid experimental-computational pipeline to study heart C-looping
• A multi-disciplinary, multi-scale study of growth-related development in C-looping
• Insights for future studies to address differential growth during C-looping
• Potential to extend the pipeline for detailed analysis of mechanisms of C-looping

## Graphical abstract

**Figure:**
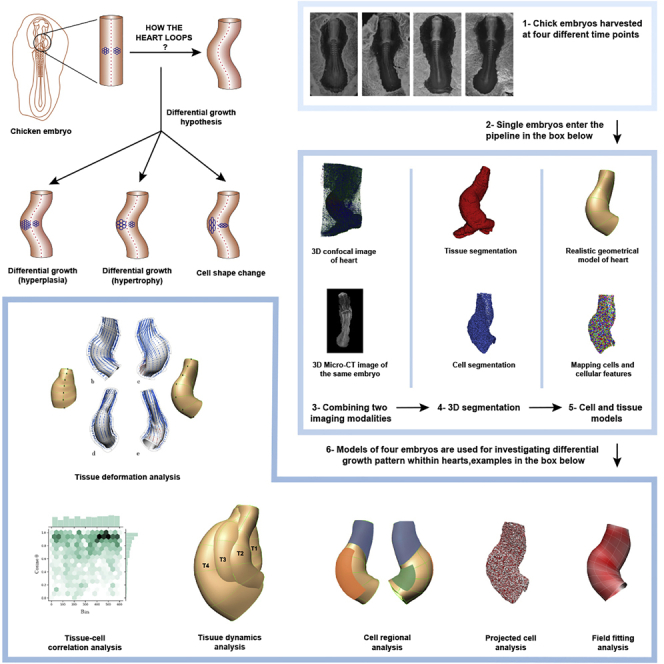

## Introduction

Heart development in chicks consists of a series of events starting with the formation of cardiac ‘fields’ (Buckingham et al., 2005), followed by linear heart tube formation. The heart tube then undergoes bending, elongation, and rotation to form a C-looped tube. The C-looped heart then transforms into an S-shaped tube through further rotational configuration changes. The looping phase is followed by cardiac septation and valve formation, giving rise to a four-chambered heart (Gruber et al., 2012; Männer, 2000). Normal looping is essential for the proper alignment of the future cardiac chambers and for inflow and outflow tracts with respect to each other (Olson and Srivastava, 1996; Harvey, 1998; Männer, 2009). Abnormal looping is associated with different congenital heart defects which are the most common birth defects occurring in almost 1% of births (Lee et al., 1998; Kathiriya and Srivastava, 2000; Hoffman and Kaplan, 2002; Reller et al., 2008). Over the past few decades considerable research has gone into understanding the process of looping. Many groups have studied the genetic and molecular basis of heart development and identified key factors involved in the looping process (Harvey, 2002; Moorman and Christoffels, 2003; Brand, 2003; Linask and VanAuker, 2007; Wagner and Siddiqui, 2007; Rana et al., 2013; Miquerol and Kelly, 2013). A number of researchers have examined the mechanical processes and proposed various mechanical forces to drive the looping (Taber et al., 1995; Voronov et al., 2004; Taber, 2006, 2014). Mathematical and computational modeling approaches have also been used to examine the proposed mechanisms (Taber and Perucchio, 2000; Latacha et al., 2005; Ramasubramanian et al., 2006; Shi et al., 2014b; Le Garrec et al., 2017; Honda et al., 2020). As a result, a number of hypotheses have been proposed to explain the looping process (Taber et al., 1995; Taber, 2014). However, none of the hypotheses have yet been fully accepted or rejected (Taber, 2014; Shi et al., 2014b). The differential growth hypothesis, which has attracted much interest in the field, addresses the potential role of differential growth of myocardial cells during looping (Moorman and Christoffels, 2003; Soufan et al., 2006; Sizarov et al., 2011; Shi et al., 2014a, b; Taber, 2014). However, gaps remain in the published data in relation to examining the hypothesis of differential cell growth. Because of insufficient data, the role of cell shape change also remains to be studied further (Manasek et al., 1972), and the cell shape change hypothesis has neither been rejected nor proven to be involved in the C-looping process. Further experimentation is therefore needed to provide more evidence. Also, a major question that remains to be answered is how geometrical changes of looping at the tissue level are linked to the growth features at the cell level.

In this study, the focus is on the C-looping phase of cardiac looping. C-looping is considered as the transformation of the straight heart tube to a C-looped shape, just before the advent of the early S-looping phase (Männer, 2009). When considering C-looping, it is important to differentiate between a) the rotational movements of C-looping, and b) the directionality of looping (the handedness of rotation). Note that the latter is not the focus of this study.

This body of research is conducted around two broad avenues of inquiry. The first is to develop an experimental pipeline to obtain data from the cell to organ level in the context of the whole embryo. The second aims to integrate the obtained experimental data into an anatomically based descriptive model of C-looping to capture corresponding localized information of cellular features and the large-scale deformation of heart shape throughout C-looping. With these two approaches we are able to address the patterns of differential growth spatially and temporally at the cell to organ level, and to investigate how tissue deformation relates to the changes of growth-related cellular features and orientation.

## Results

### A cell to organism 3D dataset for the C-looping heart from a single embryo

**Figure 1:**
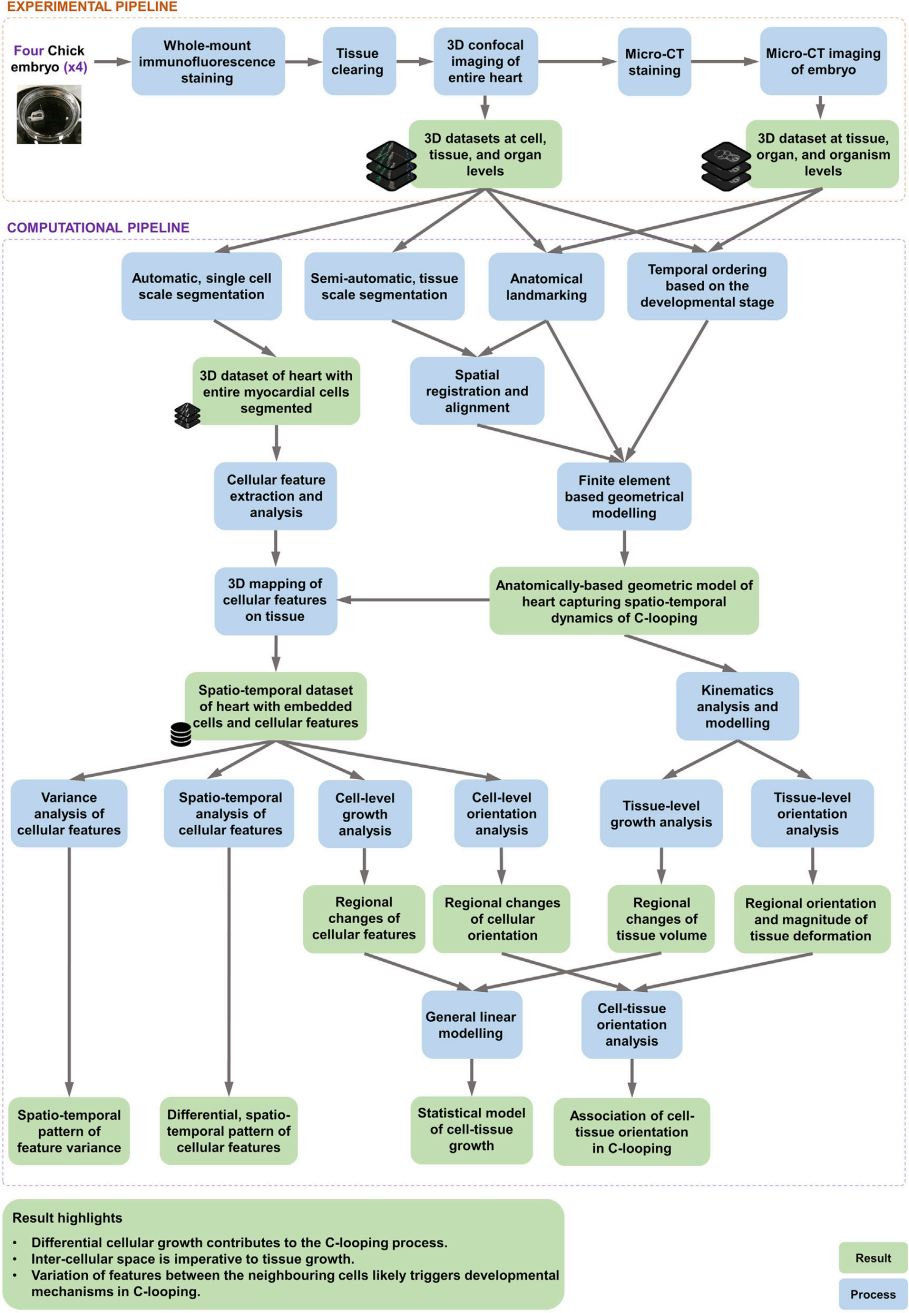
A comprehensive pipeline for 3D multi-scale study of C-looping This figure shows an overview of the pipeline used in this study. For a full, comprehensive detail on every aspect in the methods please see STAR Methods. The workflow is composed of two main parts, the experimental workflow and the computational workflow. In the experimental workflow, fertilized chicken embryos were harvested at HH10 and HH11 gestation age. Whole-mount fluorescent staining was performed to obtain a 3D image of the whole heart with individual cells labeled. Embryos were then serially incubated in Glycerol for optical clearing of the whole tissue. Using a confocal microscope, entire heart area imaged on custom made chamber for mounting whole embryo. A following 3D reconstruction of confocal images resulted in a super-image of the whole chicken heart that includes information from cell level through to the whole organ. Next, we were interested to obtain information on the tissue to organism levels from the same heart samples. Upon the completion of confocal imaging, tissue samples were washed and stained to be imaged with a micro-CT scanner at sub-micron resolution to acquire a 3D image stack of the chicken embryos, capturing information from tissue level to whole organ. Ultimately, this experimental workflow resulted in two sets of 3D dataset: (1) data at cell, tissue, and organ levels, and (2) data at tissue, organ, and organism levels. These datasets were subsequently used in the computational workflow. To begin the computational analysis and modeling, a shape representation of the heart was needed. To define an anatomically realistic shape, we segmented the geometry of the heart using a custom-made, semi-automatic pipeline, in Amira software. The segmentation resulted in labeled masks from which digitized data points were sampled to generate a 3D point cloud of the chicken heart. Using the Finite Element Method (FEM), a template mesh was constructed using high-order shape functions to mathematically represent the anatomy of hearts. We morphed the template mesh by using fitting techniques to fit and customise mesh to the 3D point cloud representation of the heart. Anatomical landmarking and temporal ordering helped to capture the spatial and temporal dynamics of C-looping. We also acquired information at cell level. We developed a fully automatic algorithm using deep learning techniques (convolutional neural networks) to segment single cells from the entire tissue. The result was a comprehensive, high-resolution single cell scale map of the myocardium. Once we had the dataset with both whole geometry and cell information, we spatially aligned all samples to a reference coordinate system to remove any confounding transformation effects. Next, in Amira software, a number of important cell features were extracted for the analysis of cell shape, volume, and orientation. This information was required to map all cell features as fields onto the constructed FE model of the heart. This resulted in a spatiotemporal dataset of heart with embedded cells and cellular feature. A comprehensive analysis of cell features and feature variance revealed differential growth patterns during C-looping. To understand how C-looping happens at the tissue level, we analyzed the growth mechanism using kinematics modeling by computing the deformation gradient tensor to describe the deformation of tissue material points from the initial time point to the next time point. Using this deformation tensor, we also obtained volume changes by computing the Jacobian from the deformation gradient. Furthermore, by performing a singular value decomposition on the deformation tensor, we obtained the tissue stretch and orientation information. The previously extracted cell features were used to measure changes of the myocardial cell number, size, and shape, and orientation throughout the course of C-looping. The resulting datasets from both the tissue- and cell-level analyses were combined to investigate their correlations during growth, and to examine whether their relationship changes spatially and temporally. The main idea for this analysis is to understand how cell-level features affect the volume and orientation changes observed at the tissue level.

**Figure 2:**
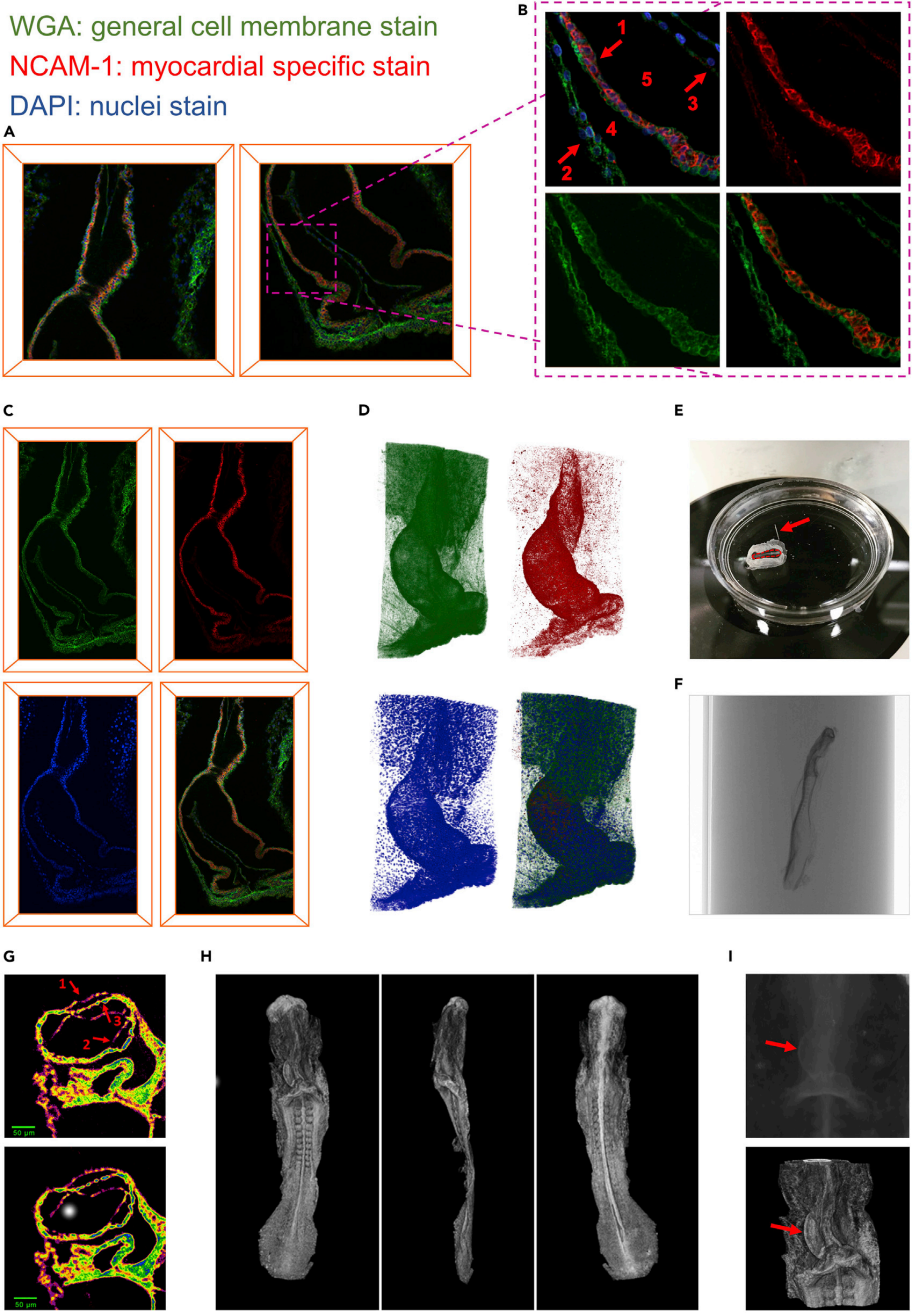
Multi-scale experimental pipeline (A–D) Whole-mount confocal staining and imaging resulted in a high-resolution 3D image of the embryonic heart and surrounding area in which cell boundaries and nuclei were labeled and the myocardial area was exclusively defined (red signal). (A) The entire chicken heart was imaged in two tiles with 15% overlap. (B) shows a zoomed area from with multi-channel signal (top left). The myocardial layer (arrow 1) is strongly stained with myocardial specific stain, NCAM-1 (red), whereas the splanchnopleure (arrow 2) and the endocardium (arrow 3) were lacking an NCAM-1 signal. Within a myocardial layer, NCAM-1 localised primarily to adjoining cell-cell boundaries and was not detectable at the myocardial cell surfaces facing the pericardial coelom and the cardiac jelly (areas are labeled 4 and 5, respectively) (top right). WGA (green), a general cell membrane stain, successfully stained the entire cell membrane within the whole sample (bottom left). In bottom right, WGA signals are co-localized with myocardial specific stain to provide a complementary and continuous signal around the cell boundaries whereas the myocardial layer is exclusively defined by NCAM-1 signal. (C) Stitching two 3D confocal image tiles with overlap resulted in the whole chicken heart image, which is shown in three channels separately and a multi-channel image. (D) shows 3D rendering of the ultimate result of the 3D confocal image stack of the entire chicken heart with the three separate channels and the multi-channel. Following confocal imaging, (E−I) a micro-CT scan on the same embryo provided tissue to organism level data. (E) A chicken embryo in a small Petri dish during C-looping. Arrow shows a micropin and dotted line shows embryonic sample for micro-CT. (F) Using cocoa butter as a mounting medium for micro-CT scanning provided an exceptionally smooth and uniform medium around the chicken embryo within a small straw (4 mm in diameter). (G) Mylar was used to reduce heat effect. In the image taken without Mylar (top), there is more collapse in the splanchnopleure layer (arrow 1), reduced sharpness (arrow 2), and weaker signal for the forming endocardium (arrow 3) in comparison with the same image taken with Mylar (bottom). (H) Micro-CT rendered image of the chicken embryo from anterior view (left), lateral view (middle), and posterior view (right). (I) Micro-CT results showed a collapse in the heart lumen and shrinkage in the whole sample.

We developed a pipeline to generate a series of characteristic 3D multi-scale datasets by combining two imaging modalities (Figures 1 and 2). The whole embryo was first used for 3D confocal imaging of the heart utilizing a clearing technique which provided cell to organ resolutions. The same embryo was then scanned by micro-CT to obtain a bigger picture of the heart in the context of the entire organism, which provided tissue to embryo resolution. This pipeline ultimately resulted in a comprehensive dataset in which data was obtained from a single sample at different biological scales. The 3D approach kept the sample’s architecture intact throughout the pipeline. Resolving the individual cell boundaries yet preserving the 3D structures of the tissue captured more localized information of cell features with respect to the large-scale spatial information of the organ, and provided a comprehensive understanding of cell organization within the tissue. To label individual myocardial cells within the developing heart, general cell membrane, myocardial-specific, and nuclei staining was carried out and resulted in a multi-channel image of the tissue (Figures 2A–2D). Myocardial-specific stains, NCAM-1 in chicken embryos, were used to identify the myocardial layer within the heart. In chicken embryos, the myocardial layer is strongly stained with NCAM-1 whereas the splanchnopleure and the endocardium are lacking an NCAM-1 signal. The outflow tract is also stained with NCAM-1. Within a myocardial layer, NCAM-1 localizes primarily to adjoining cell-cell boundaries and is not detectable at the outer free cell surfaces facing the pericardial coelom (Figure 2B). NCAM-1 also showed no signal at the cell surfaces of the inner layer of myocardial cells facing the cardiac jelly (Figure 2B). Therefore, continuously formed signals around myocardial cells are missing in most regions within the myocardial layer. Because NCAM-1 showed a non-continuous signal around cells, WGA staining was used as a complementary signal to be able to visualize the complete cell boundaries (Figure 2B). WGA globally stained all cell membranes within the tissue. Because WGA stains all cells within the tissue, a combination of the WGA and NCAM-1 channel was used for automated cell segmentation (more details later in this paper). DAPI was also used to stain nuclei. DAPI successfully passed through the cell membranes within the tissue and stained nuclei (Figure 2B). Overall, triple labeling allowed us to generate a high-resolution image of the embryonic heart and surrounding area in which cell boundaries and nuclei are labeled and the myocardial area was exclusively defined. The chicken heart was imaged in tiles with 15% overlap, and 2D and 3D images of the whole heart was successfully obtained through stitching and rendering of image tiles.

Micro-CT image stacks provided a dataset by which hearts could be examined in the context of the whole organism (see Video S1). Micro-CT scanning of embryonic samples has challenges. For instance, chick embryos are tiny in size, flat, too delicate to handle, and prone to drying out very fast (Figure 2E). We overcame these issues by using cocoa butter as a mounting medium. Cocoa butter provides an exceptionally smooth background around the sample (Figure 2F) and holds the chicken and rat embryos without any physical damage or pressure on the specimens. It also prevents the sample from drying out. Imaging at sub-micron resolution, as another example, results in increased heat delivered to the sample. Heating causes thermal drift which affects the quality of the image. To minimize this problem, layers of Mylar were used around the straw. Mylar helps to reduce the heat load by reflecting the radiated heat (Gerneke et al., 2017). Using the developed protocol, the image resolution was improved to a sub-micron level of about 0.5 microns. The result showed that all cardiac layers were stained, even the forming endocardium (Figure 2G). To validate this result, a histology staining was performed (Figure S1) and result confirmed that the single cell layer of the forming endocardium during early development of the heart was resolved in the micro-CT image. The chicken heart was imaged at 0.5 - 0.6 μm and the whole embryo was imaged at 1.4–1.6 μm to fit the entire specimen in one image stack. It was possible to image the whole embryo at 0.5 μm resolution using over-sized scanning where overlapping fields could be acquired and stitched together (see Video S1). However, taking an image of the entire organism was not necessary for this study. Reconstruction of the micro-CT image stack resulted in a 3D image of the whole embryo (Figure 2H). Micro-CT results showed a collapse in the heart lumen and shrinkage in the whole sample (Figure 2I). Therefore, the geometry of the heart was obtained from the confocal images.

Video S1. 3D MicroCT of chick embryo, related to Figure 2

To our knowledge, this is the most comprehensive, multi-scale 3D dataset available in this field, allowing for the examination of the differential growth and cell shape change hypotheses.

### Realistic computational models of four hearts captured the spatiotemporal dynamics of C-looping

**Figure 3:**
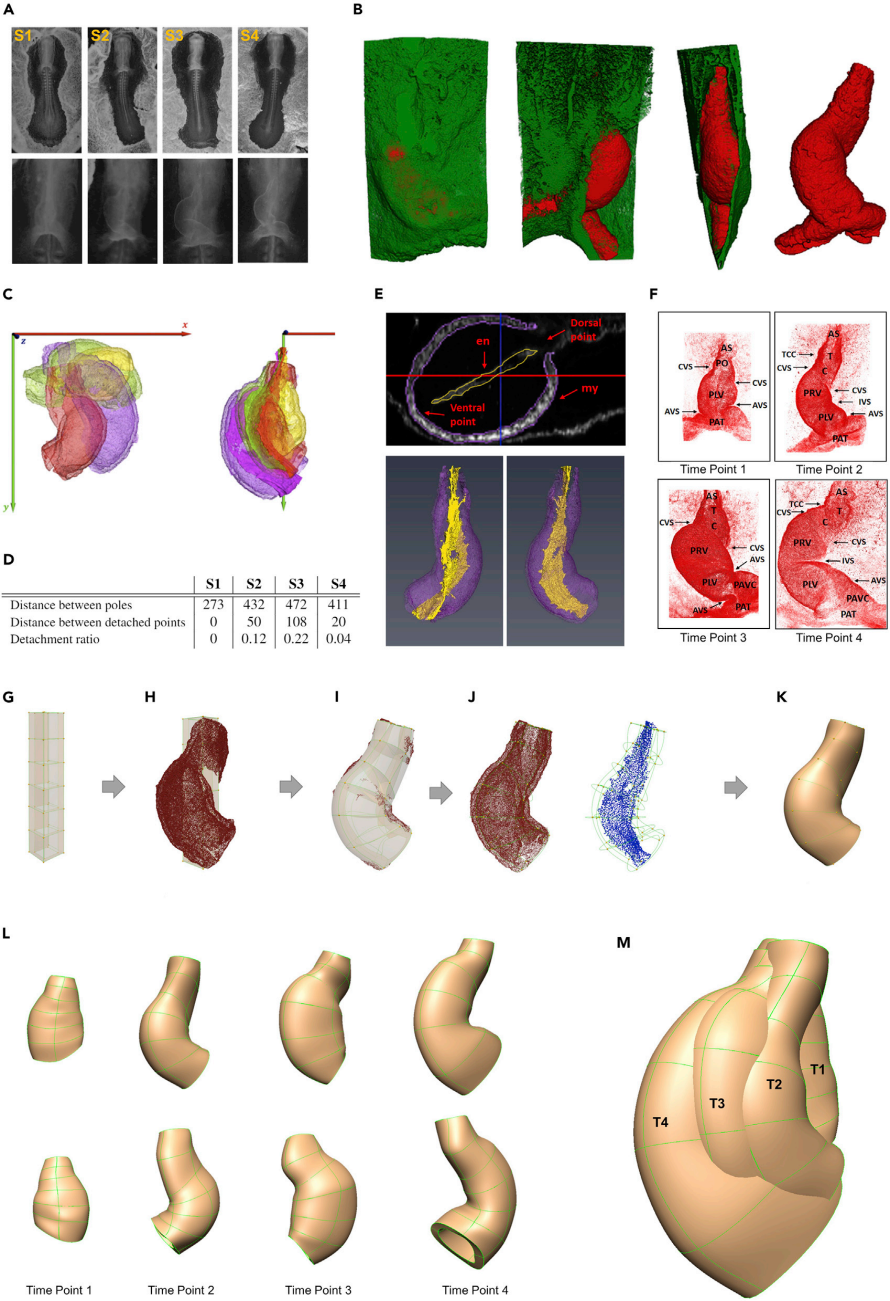
Realistic geometry FE model of four hearts captured the spatial and temporal dynamics of C-looping (see Video S2) (A) To add the temporal dimension, four chicken embryos within C-looping window, S1 to S4, were selected. The images of the whole embryos (top panel) and hearts (bottom panel) were taken using a dissecting microscope. The embryos are not temporally ordered in developmental stages. (B) shows a segmentation result in which heart is precisely segmented from the adjacent layers. Red shows the myocardial layer (NCAM signal) segmented out from the non-myocardial layer (green, WGA signal). (C–F) Capturing the spatial and temporal dynamics of the four independent harvested embryos. (c) Four hearts were aligned with no scaling with respect to the global axes: the figure shows original location of samples in the global coordinate system (left inset) and the myocardial and endocardial layers after alignment (right inset). It also shows overall rotation of hearts and endocardial layer with respect to the embryonic midline which was used for the temporal staging of the samples. Different colors are used for different samples for visualization purpose. Darker shades are used for endocardial layer in the right inset. (d) The level of dorsal detachment from the dorsal wall was measured from micro-CT data to order the embryos temporally in four sequential Time Points 1 to 4 (distances are expressed in μm). (e) The endocardium is used to identify the dorsal and ventral lines of the heart. Top panel show a cross-section of the confocal image in which ventral and dorsal points were defined with respect to the endocardium. Dorsal (bottom left) and ventral (bottom right) views of the 3D reconstruction of the endocardial layer (yellow) within the myocardial layer (purple) defined the dorsal and ventral lines of the heart. (f) Anatomical landmarking was performed and different regions were marked within the heart using lateral furrows (arrows) that divided the heart into the anatomical regions. (G–M) Shows an overview of fitting an FE mesh to the image. (G) A template FE mesh was generated in OpenCMISS-Zinc. (h) Heart data points were loaded onto the template mesh in OpenCMISS-Zinc. (I) An OpenCMISS geometric fitting and smoothing were performed to fit the mesh to the data. (J) Anatomical landmarks and endocardial layer were used to fix the control points at their position. (K) A smooth subject-specific FE mesh was generated after a few iterations. (L) Fitted volume meshes of heart subjects at four time points during looping were developed. Top panel shows the ventral view of the heart at Time Point 1 and the view of the outer curvature at the other time points. Bottom panel shows the dorsal view of the heart at Time Point 1 and the inner curvature view at the other time points. (m) Four overlaid meshes show the dynamic changes of the heart geometry with respect to each other at sequential temporal points from the straight heart tube to the C-looped heart. T1,…,T4: Time Point 1,…,Time Point 4. AO: Aortic Sac; AVS: atrioventricular sulcus; C: primitive conus; CVS: conoventricular sulcus; en: endocardium; my: myocardial layer; PAT: primitive atrium; PAVC: primitive atrioventricular; PLV: primitive left ventricle; PO: primitive outlet; PRV: primitive right ventricle; T: primitive truncus.

To investigate the spatiotemporal dynamics of C-looping, the Finite Element (FE) method was used to develop a realistic geometric model of C-looping (Figure 3G–3M). First, four looping hearts were selected at different time points of development (Figure 3A). Utilising a semi-automatic 3D segmentation approach, the geometry of four hearts were extracted where the myocardial layer is precisely segmented from the adjacent layers (Figure 3B) (see also STAR Methods, Figures S2 and S3 for details). Because the subjects were obtained from different embryos, different approaches were taken to capture the spatiotemporal dynamics of data. For spatial registration, the four hearts were aligned with respect to the global coordinate system and were overlaid using a rigid transformation with no scaling which resulted in capturing the spatial dynamic of the C-looping (see Spatial registration in STAR Methods). To find the temporal order of the samples, two approaches were used: (i) rotational degree of the endocardial level (Figure 3C), and (ii) detachment level of dorsal endocardium (Figure 3D). The samples are temporally ordered as follows: embryonic samples S1, S4, S2, S3, respectively refer to the Time Points 1 to 4 during C-looping. This temporal ordering is further confirmed by the total tissue volume and number of cells of the myocardial layer within each time point after tissue and cell segmentation were carried out (Figure S10). In addition, we performed anatomical landmarking to define the boundaries of the heart area and the corresponding points and regions at different time points (see Anatomical landmarking section in STAR Methods for details) (Figure 3F).

For FE modeling, an initial bicubic-linear tube mesh was generated which was then fitted to each subject’s heart data points. For this, we used the OpenCMISS software (http://opencmiss.org/about.html). Heart data points were accurately digitized and extracted using the myocardial layer from the 3D confocal images of the heart (Figures 3G, 3H,S4, and S5). Using OpenCMISS (Bradley et al., 2011) geometric fitting and smoothing were performed, and anatomical landmarks and the endocardial layer were used to fix the control points at their position (Figures 3I and 3J). A smooth subject-specific FE mesh was generated after a few iterations (Figure 3K). This approach ultimately resulted in a mathematical shape model of the realistic geometry of C-looping in which corresponding regions of the heart are traceable both spatially and temporally (see Figures 3L, 3M and Video S2). The model was optimized to obtain a reasonably good Root-Mean-Square (RMS) fitting error with a smooth uniform mesh which would be applicable to further mechanical and geometrical analyses. An RMS error of less than 3 μm was achieved for all subjects, indicative of accurate fitting (relative to the size of the heart i.e., more than 1 ×10^6^$\mu m^{3}$) (see STAR Methods for details on FE method). Please note that the dorsal opening was not integrated in the FE model. We noticed that this feature caused significant and unnecessary mathematical and computational complexities in the fitting procedures and kinematics analysis. Consequently, we decided to close up that region in the model but exclude that region in all of the computations and analyses. These geometries also provided the basis to link the cell, tissue, and organ levels for an anatomically based analysis of the C-looping process.

Video S2. From 3D confocal image to 3D geometric model, related to Figures 2 and 3

### Automatic 3D cell segmentation algorithm reconstructed the full heart with labeled single myocardial cells

**Figure 4:**
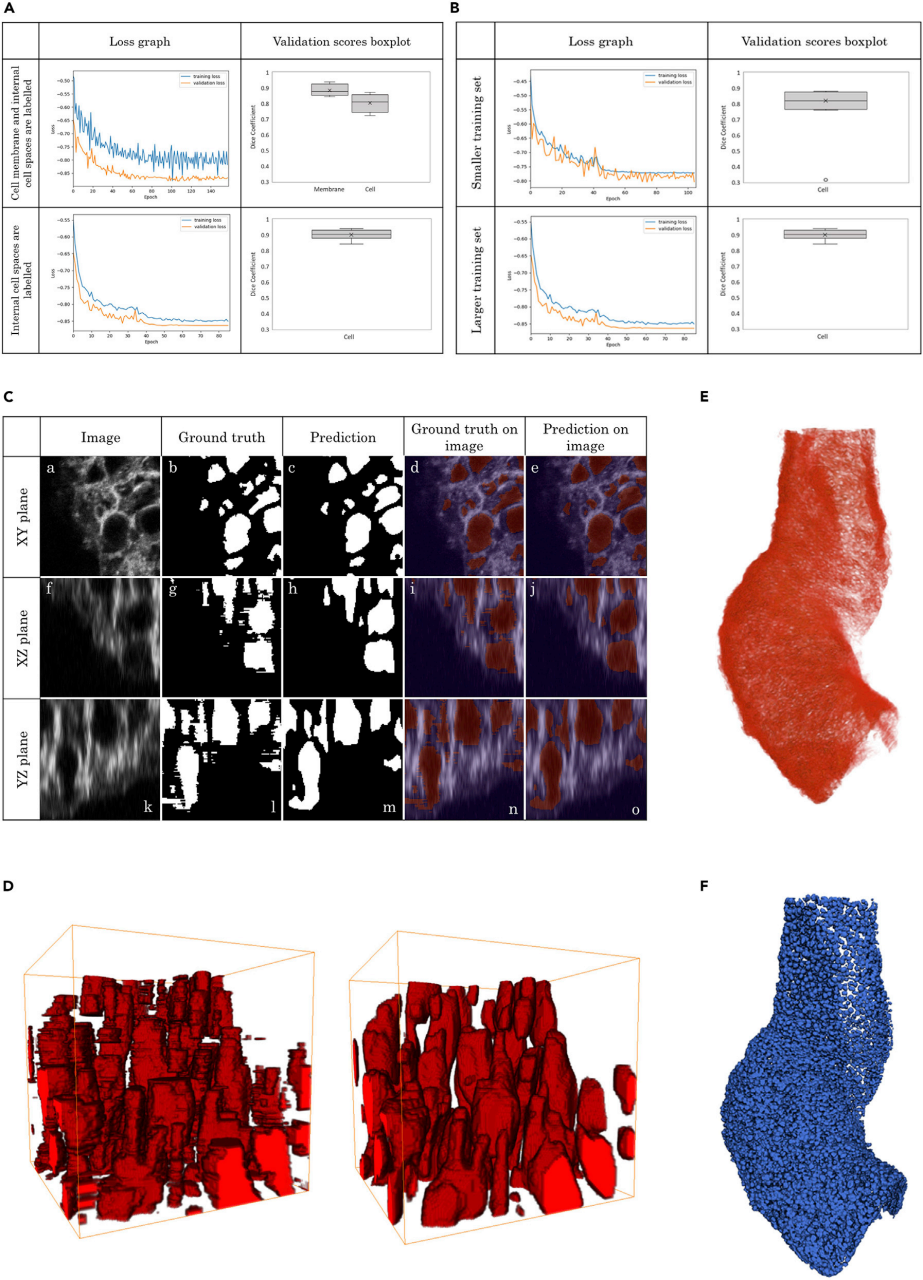
Automatic 3D cell segmentation algorithm resulted in a 3D dataset of heart with entire myocardial cells segmented (A and B) The performance and accuracy of each training protocol was assessed by analyzing the loss function graph and the dice similarity coefficient box-plot (see STAR Methods). (A) An assessment was performed to compare training results with different class labeling approaches. The first scenario involved labeling background, cells, and membranes as three different classes to segment cells and membranes. In a second scenario only the background and cells were labeled; (B) Deep learning models are known to be affected by the size of training data. Here an assessment was done with two separate training datasets: one with a small dataset (top) and another with a larger dataset (bottom). Behavior of the segmentation model with the larger dataset is greatly improved during training with the loss graph demonstrating a less noisy fitting and a faster learning. Furthermore, the evaluation dice coefficient is improved in the case of the larger dataset (median =0.92) in comparison to the smaller dataset (median =0.80). (C) Myocardial cell prediction for one confocal image viewed in three different planes. The channels (signals) represent complementary staining of myocardial cell membrane with WGA and NCAM-1. (D) A three-dimensional view of myocardial cells from manual segmentation (left) and our developed automatic method (right) revealed a smoother and more consistent extraction of cell features using the latter, as shown by the comparison between the prediction and ground-truth images. (E) 3D rendered image of confocal image stacks which was subjected to the cell segmentation (see Video S3). (F) Final cell segmentation result of the corresponding heart using the developed automated cell segmentation algorithm (see STAR Methods for full detail) resulted in a 3D reconstructed binary dataset of heart in which individual myocardial cells were segmented.

A 3D convolutional neural network was built to segment and extract individual cells from the 3D confocal image stacks (Figures 4 and S6). A large number of training protocols were tested to eventually derive the most accurate cell segmentation results. The performance and accuracy of each protocol was assessed by analyzing the loss function graph and the dice similarity coefficient box-plot (see STAR Methods). The overall mean dice coefficient between the method we developed in this study and the expert manual segmentation was 0.91 ± 0.1 (mean ±SD) for the entire test set (N = 25). (Figures 4A and 4B). An example of the segmentation prediction for one image slice is shown in Figure 4C. The time required for prediction of segmentation using the automated method was only a few seconds per approximately 100 cells whereas the manual segmentation by a trained expert takes approximately 8 h for 100 cells. The automated neural network pipeline produced consistent segmentations with improved regional details in all planar directions (Figure 4C). In addition, the model was able to generalize the prediction to generate smoother and more controlled cell segmentations with less noise (Figures 4D and Video S3). This is shown in Figure 4D where the three-dimensional view of the segmentation result of one image shows a more consistent segmentation with less noise using the U-net model. The resulting slices with cell segmentation for a given heart were merged into their original tiles. The confocal tiles were then stitched to reconstruct the full heart with individual segmented cells. Sub-Figures 4E and 4F shows an example of the a reconstructed heart from NCAM-1 channel and final, full reconstruction of a segmented heart.

Video S3. Deep learning 3D segmentation of myocardial cells, related to Figure 4

Taken together, the developed workflow in this section resulted in four datasets of 3D binary image stacks at different time points from a straight heart tube to a C-looped heart, in which myocardial cells within the entire heart were extracted. In addition, the designed approach (traceable slicing-merging algorithm - see STAR Methods for detail), which overcame the issue of feeding large image data into the computer memory, offers the possibility to run automatic large-scale image segmentation and analysis on much larger image sizes without losing image quality and information.

### Comprehensive 3D mapping of myocardial cells onto the geometric model exposed patterns of cell features during C-looping

**Figure 5:**
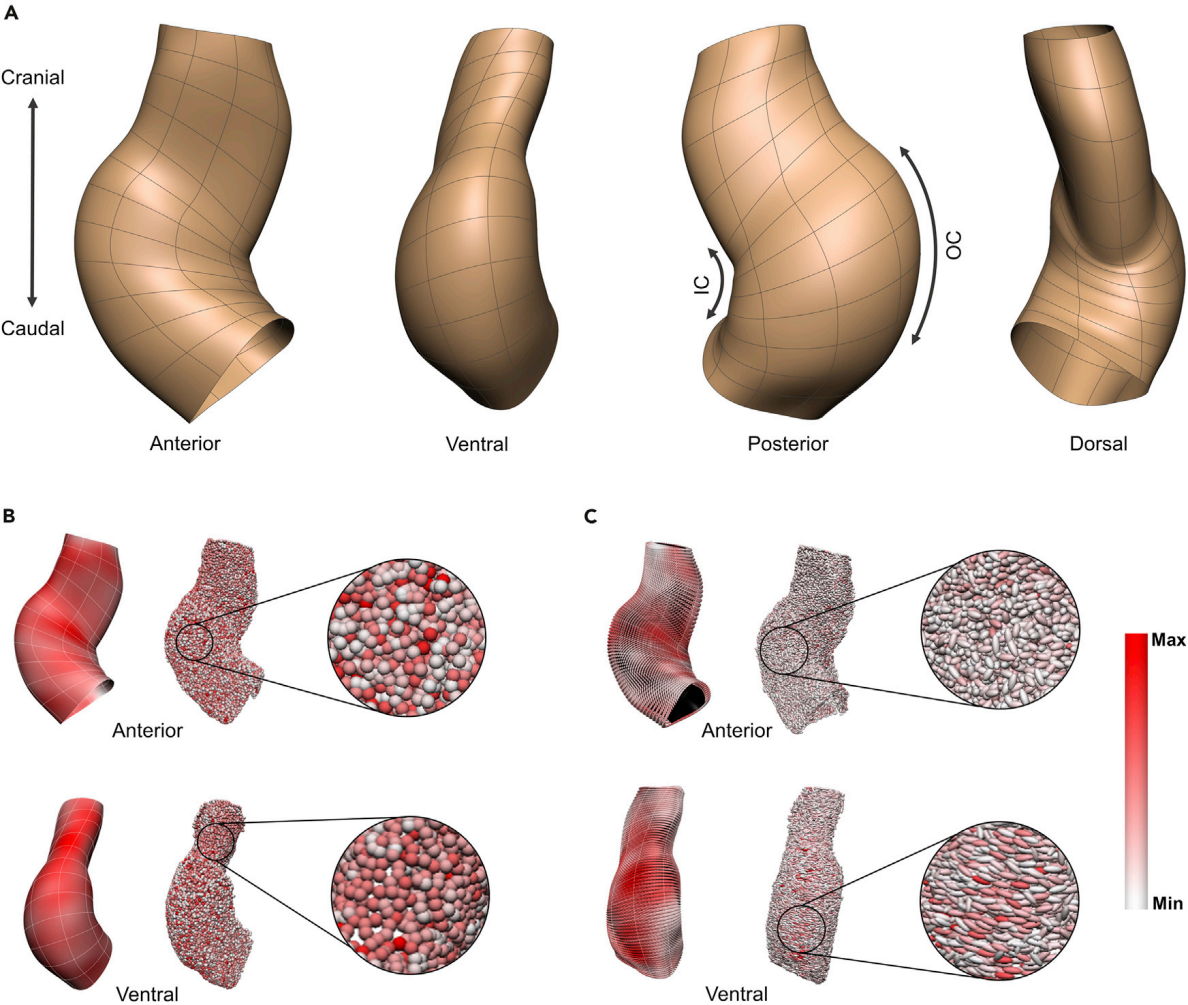
3D mapping of cellular features onto the geometric model (A) Shows anatomical views and terminologies used in this paper which are based on the position of the heart in space and not the embryo. (B and C) Different features of the individual segmented myocardial cells such as volume, anisotropy, and orientation were measured and quantified. The resulting data points from individual myocardial cells inheriting the measured values for the different cell properties were mapped to the surface of the corresponding realistic FE geometry of the given sample. Next, either a field fitting approach was employed which captured the variations in the mean values of cellular features at a larger scale, or individual cells with their raw values of measure were projected on the corresponding refined geometry mesh which exposed discrete patterns of cellular features spatially and temporally (see STAR Methods for details on the techniques, and Video S4). (B) Results of field fitting and projected cell data for cell volume for Time Point 2 are shown: the left column shows the result of fitting cell volume over the heart geometry as a scalar continuous field and the middle column shows individual cells in their projected coordinates as discrete points with their values for cell volume color-coded. The discrete cell level data shows, in general, a more variable pattern than a continuous field. Some regions were more heterogeneous (zoomed area in top right), and other regions were more homogeneous (zoomed area in bottom right), and therefore had a different mean value as can be seen in the field fitting result. A white to red spectrum represents the range for cell volume from Min (132 $\mu m^{3}$) to Max (355 $\mu m^{3}$) for fitted data, and from Min (4 $\mu m^{3}$) to Max (750 $\mu m^{3}$) for cell data. (C) Results of field fitting and projected cell data for cell orientation for Time Point 2: ellipsoids show the orientation of cells (using the principal eigenvector). The glyph shapes show cell sphericity using the cell measured anisotropy value. A white to red spectrum represents the long-axis lengthening from least elongated to the most elongated cells (using the principal eigenvalue). Note that the size of the glyphs is not scaled with the volume of the cells, therefore there is no correlation between anisotropy (glyph shape) and the long axis stretch (spectrum) in this figure. The fitted values of the three components of the principal eigenvector, principal eigenvalue, and anisotropy are used to align, color-code, and shape the glyphs on the surface (left). Note that glyphs do not represent individual cells, but rather are evenly distributed on the surface mesh. The fitted field result shows the pattern for the mean cell orientation and elongation. For example, cells were aligned circumferentially and elongated more in the outer curvature region (bottom left). The raw values of the measured covariance matrix components (principal eigenvector and eigenvalue) and anisotropy were used to align, color-code, and shape each cell ellipsoid (middle). Glyphs represent individual cells. In most regions, cells did show any favored directionality and showed a heterogeneous pattern for shape and sphericity (zoomed area in top right). In some regions, however, there was a preferred orientation (zoomed area in bottom right). For example, the majority of cells were aligned circumferentially and were mostly elongated in the outer curvature. The spectrum range for the eigenvalue is from Min (4.3 μm) to Max (11 μm) for fitted data (left), and from Min (0.4 μm) to Max (58 μm) for cell data (middle). OC: outer curvature; IC: inner curvature.

**Figure 6:**
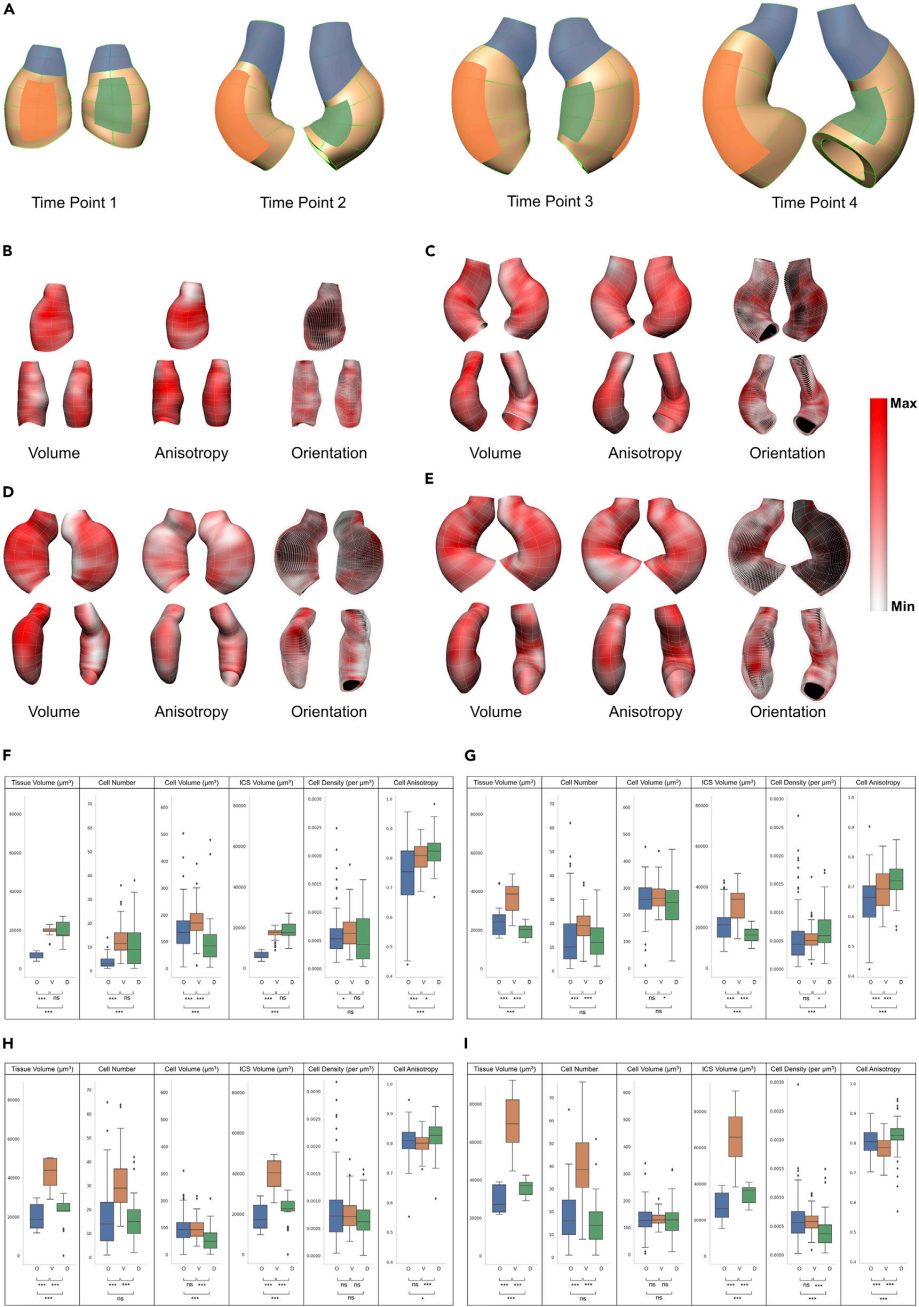
Regional spatial pattern from field fitted and projected cell data (A) Shows three defined regions including OFT (red), ventral (blue), and dorsal (green) overlaid on volume meshes of heart subjects at four time points from two views for visualization. Top panel shows ventral view of the heart at Time Point 1 and outer curvature at the other time points. Bottom panel shows the dorsal view of the heart at Time Point 1 and inner curvature at the other time points. (B–E) Shows spatial pattern from field fitting at four time points. Fitted values of cell volume, anisotropy, and orientation components (principal eigenvector and eigenvalue) are visualized over the heart geometry to examine spatial patterns within each time point. A white to red spectrum shows the minimum to maximum values in the adjusted range. Note that the dark black color in (b-e) appear as a result of the background color of the inner layer of the heart model when the glyphs are pointing perpendicular to the plane, leaving empty spaces between glyphs. (b) At Time Point 1, the cranial part of the ventral region had larger cells. Cells in the middle to the cranial part of the ventral region were more elongated. The OFT region contained smaller, more spherical cells. Lateral regions showed, in general, a patchy pattern. In terms of orientation, cells were mostly unaligned at this stage. The minimum to maximum values for cell volume: $29\mu m^{3}-240\mu m^{3}$; anisotropy: 0.65−0.87; and principal eigenvalue: 2μm−16.6μm. Top row: anterior view, Bottom: lateral views. (C) At Time Point 2, the OFT region and the cranial part of the outer curvature shows regions with larger cell volume. The middle part of the heart including outer and inner curvature regions, showed more elongation. Cells were oriented circumferentially in the outer and inner curvature regions and more longitudinally in lateral regions. The minimum to maximum values in the adjusted range for cell volume: $132\mu m^{3}-355\mu m^{3}$; anisotropy: 0.5−0.85; and principal eigenvalue: 4.3μm−11μm. Top-left: anterior view, Top-right: posterior view, Bottom-left: ventral view, and Bottom-right: dorsal view. (D) At Time Point 3, smaller cells were located along the dorsal line and caudal part of the heart. The anisotropy and principal eigenvalue pattern showed a few regions with more elongated cells in the outer and inner curvature regions. The orientation pattern revealed circumferentially oriented cells in the outer and inner curvature regions. In the anterior view, cells were oriented longitudinally in the lateral region. The minimum to maximum values in the adjusted range for cell volume: $7.6\mu m^{3}-190\mu m^{3}$; anisotropy: 0.74−0.92; and principal eigenvalue: 1.2μm−16μm. Top-left: anterior view, Top-right: posterior view, Bottom-left: ventral view, and Bottom-right: dorsal view. (E) Shows the C-looped heart at Time Point 4. Cell volume showed a patchy pattern, however, some regions exist with larger cells in the outer curvature region. From the anisotropy and principal eigenvalue patterns more elongated cells were seen in the outer and inner curvature regions and, also, along the ventral line in the OFT region. Cells were oriented circumferentially in the outer and inner curvature regions, and longitudinally in the lateral regions. The minimum to maximum values in the adjusted range for cell volume: $52\mu m^{3}-195\mu m^{3}$; anisotropy: 0.71−0.87; and principal eigenvalue: 4.2μm−14μm. Top row: anterior view, Bottom: lateral views. (F–I) Shows regional spatial comparisons from projected cell data at four time points. Averaged values in bins for different cellular features were compared between three regions, the OFT region (blue), the ventral region (orange), and the dorsal region (green). Different parameters were plotted and a Mann-Whitney U test was carried out for statistical significance. Since multiple two-way tests between the three regions were performed, a Bonferroni correction was applied to correct for these multiple comparisons. Boxplots show median, first, and third quartile (box), minimum and maximum values (whiskers), and the outliers (small diamonds). Statistical significance for differences between groups are provided below the bars: p≥.05 (ns), p<0.05 (∗), p<0.01 (∗∗), and p<0.001 (∗∗∗). Full test statistics are presented in Tables S9–S12. O, OFT; V, ventral; D, dorsal. (F) At Time Point 1, ventral and dorsal regions had greater values for tissue volume, cell number, and ICS volume and more elongated cells than the OFT region. There was no significant difference between the dorsal and ventral regions for these. The ventral region had significantly larger cells and cell density than both the OFT and dorsal regions. The OFT showed the most variation in cell shape. (G) At Time Point 2, the ventral region had the largest tissue volume with a significantly greater number of cells and a greater ICS volume than the OFT region and dorsal region. Cell volume did not have significant differences between regions. The OFT region had a larger ICS volume than the dorsal region but there was no difference in the number of cells between these two regions. The dorsal region showed the greatest density with most elongated cells. (H and I) At Time Point 3, the larger tissue volume with a significantly larger number of cells and a higher ICS volume were observed in the ventral region. The dorsal region had more elongated cells. There was no significant difference in the average cell volume and cell anisotropy between the OFT and ventral regions. Cell density did not show any significant difference between the three regions (I) Time Point 4 showed a similar pattern to Time Point 3 for the tissue volume, cell number and ICS volume with the ventral region had the greatest value for all these parameters. The OFT region had smaller tissue and ICS volumes than the dorsal region, but there was no difference in the number of cells between these two regions. There was no significant difference in the cell volume between all three regions. The dorsal region showed a significantly lower density of cells. The dorsal and ventral regions showed the largest and smallest values for anisotropy.

**Figure 7:**
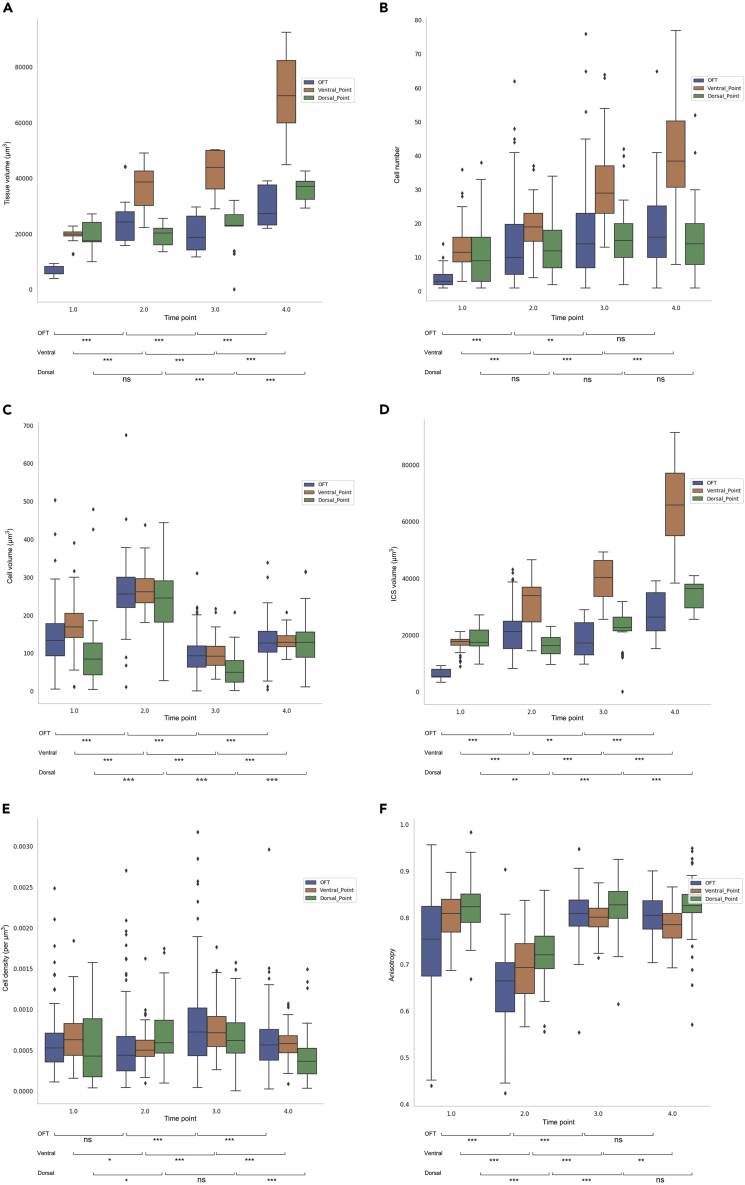
Regional temporal pattern of from projected cell data Different features were studied separately over the four time points to examine temporal changes in the OFT region (blue), the ventral region (orange), and the dorsal region (green). The values of the individual bins within the defined regions were plotted. Boxplots show median, first, and third quartile (box), minimum and maximum values (whiskers), and the outliers (small diamonds). The changes of a given parameter between two subsequent time points were tested using a Mann-Whitney U test. Statistical significance for differences between groups are provided below the bars: p≥.05 (ns), p<0.05 (∗), p<0.01 (∗∗), and p<0.001 (∗∗∗). Full test statistics are presented in Tables S13–S18. (A) The volume of bins in the OFT region increased from Time Point 1 to 2, then decreasd at Time Point 3, followed by an increase from Time Point 3 to 4. The ventral region showed a continuous increase from Time Point 1 to 4. In the dorsal region the volume of bins did not change between Time Point 1 and 2, then increased from Time Point 2 to 4. (B) The number of cells in the OFT region increased from Time Point 1 to 3, but there was no significant change from Time Point 3 to 4. There were significant increases in the ventral region in all time periods. In the dorsal region, on the other hand, there were no significant changes throughout the four time points. (C) The average volume of cells in all three regions increased from Time Point 1 to 2, then dropped at Time Point 3, followed by an increase from Time Point 3 to 4, however, did not reach the cell volume of Time Point 2. (D) The ICS volume at the OFT region increased from Time Point 1 to 2, then decreased from Time Point 2 to 3, followed by an increase at Time Point 4. In the ventral region, the ICS volume increased significantly over time. In the dorsal region, there was a decrease from Time Point 1 to 2, followed by increases at Time Points 3 and 4. (E) The density in the OFT region showed no change between Time Point 1 and 2, then increased at Time Point 3 and decreased at Time Point 4. In the ventral region, the density decreased between Time Point 1 and 2, then increased during the next time period which was followed by a decrease from Time Point 3 to 4. In the dorsal region, the density increased from Time Point 1 to 2, then remained unchanged between Time Point 2 and 3 and dropped significantly from Time Point 3 to 4. (F) Within the OFT region, cells changed toward more spherical shape from Time Point 1 to 2, then elongated again at Time point 3 and remained unchanged between Time Point 3 and 4. Cells in the ventral region were more spherical at Time Point 1 and 3 in comparison with Time Point 2 and 4. The dorsal region showed the same trend as the OFT region.

Different features of the individual segmented myocardial cells such as volume, anisotropy, and orientation were measured and quantified. The resulting data points from individual myocardial cells inheriting the measured values for the different cell properties were mapped to the surface of the corresponding realistic FE geometry of the given sample (see STAR Methods for full detail on the techniques, also Table S1, Figures S7, and S8). Two 3D mapping approaches enabled the study of cellular features from two different aspects and at two different scales (see STAR Methods for detail). Note that, for consistency and coherency of analysis, Figure 5A provides an overview of the terminologies used for the anatomical orientation of the heart embryo throughout this paper. A mapping approach used field fitting which captured patterns in the larger scale spatial variations. Projection of the cell data was used as second mapping approach resulted in a spatiotemporal dataset of discrete cellular features (Figures 5B and 5C). Although cellular features showed a heterogeneous pattern and, consequently, extraction and visualization of patterns proved difficult from the projected single cells, constructing a spatial dataset of the original data was important. First, the original data was always used to confirm the result of field fitting because in regions where fitting showed a pattern the cell feature was less variable (Figures 5B and 5C). Second, the quantitative analysis and statistical tests were performed using this dataset to increase the data resolution and analysis accuracy (Figures 6F–6I, 7, S15, and Tables S3–S18).

The 3D mapping of cells and cellular features onto the FE geometry of a given subject resulted, for the first time, in a dataset for each heart in which the individual myocardial cells have local coordinates and measured values (see Video S4). Because the FE models of the heart embedding this cellular information are also aligned, these datasets exposed the spatial and temporal patterns of various cellular features with regard to the heart geometry, qualitatively and quantitatively, during C-looping.

Video S4. 3D mapping of cellular data, related to Figure 5

### Region-based analysis showed differential pattern for growth-related features

We performed a comprehensive analysis on growth related parameters using the generated spatiotemporal datasets of geometric models with embedded cells and cellular features, for example cell volume (for size), cell anisotropy (for shape), and cell orientation (for directionality). Entire heart analyses were first carried out to study the overall changes of features between different time points (see Note S1, Figures S9, S10, Tables S3, and S4). For regional analysis, three regions of the OFT, ventral, and dorsal regions were compared (Figure 6A). These analyses revealed (i) the spatial pattern of features over the geometry within each time point (Figures 6B–6E, and S11–S14), (ii) the spatial changes of features from projected cell data within the four time points (Figures 6F–6I), (iii) the temporal changes of cellular features within the three regions of OFT, ventral and dorsal (Figure 7), (iv) overall spatial pattern with respect to the temporal changes (Figure S15), and (iv) overall temporal pattern with respect to the spatial changes (Figure S15). Presenting the full result of the study is beyond the word limit of this paper; therefore, the results from the spatial and temporal analysis of myocardial cell features during C-looping are fully presented in the Note S1. This section describes only a number of highlighted results from the spatiotemporal analysis. Our results showed a region-based differential pattern for cellular features. Interestingly, both spatially and also temporally.

During the first time period (Time Point 1 to 2), where the heart tube undergoes ventral bending, our results showed an increase in the total volume of the heart over time (see Note S1). Spatial comparison of the dorsal region with the ventral region showed that at Time Point 1 there was no difference in the tissue volume, the number of cells, the inter-cellular space (ICS) volume, and the density between the two regions (Figure 6F). At Time Point 1 only the cell volume was greater in the ventral region than the dorsal region, whereas after ventral bending at Time Point 2 the ventral region showed a greater value for all of the cellular features above (Figures 6G and S15). This clearly illustrates higher growth in the ventral region and provides quantitative data of differential growth between the ventral and dorsal regions with regards to growth-related features of myocardial cells. In terms of cell shape changes (anisotropy), all regions showed a similar pattern where cells changed their shape toward a more spherical shape. The spatial pattern at the two time points was also similar. The dorsal region had the most elongated cells and the OFT region had the most spherical cells (Figures 6F, 6H, and S15). Spatiotemporal analysis revealed that both the OFT region and the ventral region exhibited significant increases in tissue volume during this time period whereas the dorsal region remained unchanged (Figures 7A and S15). From a cellular growth point of view, the growth in the volume of both the OFT and ventral regions was accompanied by an increase in number and volume of cells and also the ICS volume in these two regions. It has been shown that the heart tube elongates by recruiting the cardiac progenitor cells from the adjacent second heart field during C-looping (Francou et al., 2013; Miquerol and Kelly, 2013; Soufan et al., 2006). It is also suggested that the heart tube elongates through the cell rearrangement (Kidokoro et al., 2018). In this study, however, we were unable to distinguish between cell proliferation, migration, and rearrangement. In the dorsal region, the number of cells remained unchanged. The increase in cell volume and decrease in ICS volume seemed to cancel each other out in this region (Figures 7B–7D). Furthermore, the cell density results showed a small increase in the dorsal region (Figure 7E). Altogether, it appeared that in the dorsal region a tissue remodeling (rather than tissue growth) was occurring.

During the second time period (Time Point 2 to 3) the rightward rotating heart underwent tissue growth but this was not as significant as during other time periods (see Note S1). At the cellular level, spatial and temporal changes were different from the previous time period. The OFT and ventral regions no longer showed a similar pattern of growth (Figure S15). In the OFT region the tissue volume decreased which was due to a decrease in the cellular and ICS volumes (Figures 7C and 7D). The number of cells in the OFT region increased slightly which resulted in an increase in cellular density (Figures 7B and 7E). The ventral region showed a significant increase in tissue volume and all cellular features except cell volume (Figures 7A–7E). The dorsal region showed growth at this time point and this was mainly due to ICS volume growth, as the number of cells did not change and the cell volume decreased (Figures 7B and 7E). Cell shape analysis showed that the three regions followed a similar pattern of change, where cells became more elongated (Figures 7F and S15). Overall, there was differential growth between the ventral and dorsal regions during this time period where the ventral region showed higher growth than the dorsal region in regard to the growth-related features of myocardial cells. The OFT and dorsal regions behaved differently when compared with the previous time period. The OFT region experienced more tissue remodeling and no tissue growth. The dorsal region, on the other hand, showed growth; however, not as a result of cellular growth (number and volume) but as a result of growth in the ICS volume. The growth pattern in the ventral region showed an increasing number of cells and ICS volume and a decreasing cell volume which could be because of more proliferation in this region and no hypertrophy of cells.

During the third time period (from Time Point 3 to 4) where the heart formed the C-looped shape, the three regions showed a similar temporal pattern (Figure S15). The tissue, cell, and ICS volumes increased in all regions but the number of cells increased only in the ventral region. The OFT and dorsal regions behaved in a similar manner with an increase in the cell and ICS volumes and a decrease in cell density (Figure 7). In the ventral region, however, significant growth at the tissue level occurred as a result of an increased number of cells (because of either proliferation or migration), cell volume (hypertrophy), and ICS volume. There was a region-based differential growth during this time period where the ventral region showed greater growth than the dorsal region. In terms of cell shape, the OFT and dorsal regions remained unchanged during this time period, whereas in the ventral region cells morphed toward more spherical shapes likely due to cell proliferation (Figure 7F).

### Inter-cellular space may have a key role in the differential growth during C-looping

Our results show that total growth is not just about cellular growth, and that growth in the inter-cellular spaces and hence the extracellular matrix has a role in tissue growth changes. Time Points 2 and 3, for instance, showed an interesting pattern where the total tissue volume did not change significantly. The number of cells and the cell density increased but the total volume of cells decreased (see Figure S10, per bin). Because the total myocardial layer consists of cells and spaces between cells, if there was an increase in total tissue volume and a smaller change in the total volume occupied by cells, then there should be an increase in the spaces between cells. From these observations, it was concluded that Time Point 2 had a denser population of bigger cells with less inter-cellular space whereas Time Point 3 had a bigger population of small cells and a larger inter-cellular space. At Time Point 4, the tissue volume increased significantly but the change in the number of cells was not significant. The density thus dropped as expected. The increase in the total cell volume was significant, but it did not reach the value seen at Time Point 2. There was a considerable increase in the volume of ICS which could explain the increase in the total tissue volume.

### Variance but not absolute measure of cellular features revealed a similar spatiotemporal pattern

**Figure 8:**
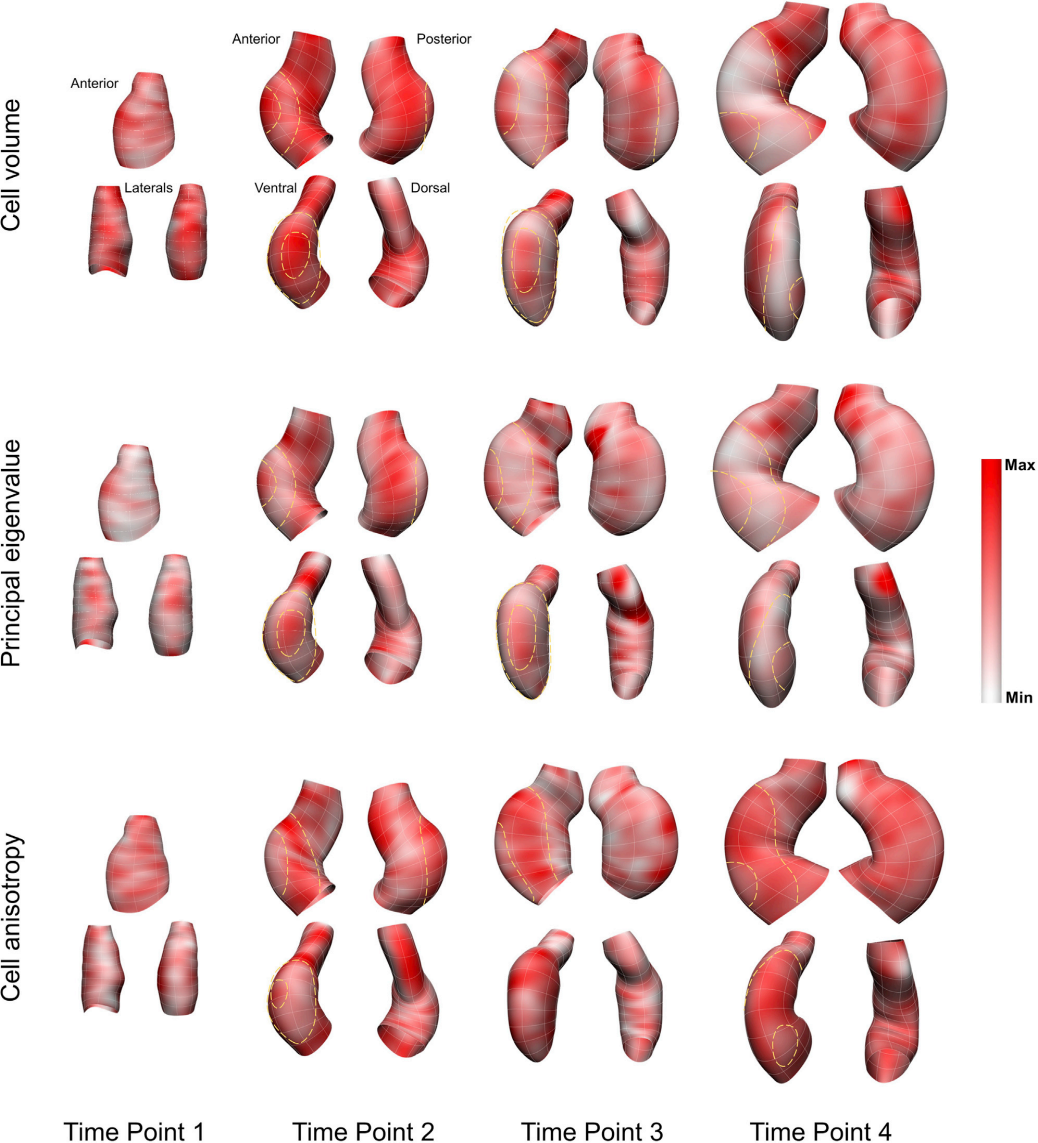
Variance analysis from field fitting showed similar pattern for different cell features Variance analysis revealed a similar pattern for the different parameters. In this figure the variance patterns of cell volume, principal eigenvalue, and anisotropy are shown. Time Point 1 showed a heterogeneous pattern for the three parameters. At Time Point 2 a ring shaped region around the outer curvature area was observed in which the cells showed a low level of variance for the cell volume and the principal eigenvalue. The ring shaped area is shown with yellow dashed lines when it is visible in a given view. Inside the ring in the outer curvature area, the cells showed a high variance for these two cellular parameters. The region with a high variance decreased in area and the ring shaped region around it grew over time. Comparison with the cell data (see Figure S16) confirmed this pattern. Between the white area and the inner curvature region there was a region with high variance. The variance for the anisotropy showed a similar but reversed pattern. The ring shaped area exhibited a high level of variance for cell anisotropy and inside the ring showed a low level of variance. The ring shaped region with high variance became larger over time. There was no clear pattern in the inner curvature region for all three parameters. The OFT region mostly had a patchy pattern at all times for all parameters. A white to red spectrum shows the minimum to maximum values in the adjusted range. Cell volume: Time Point 1: 9454$\mu m^{6}$ - 29,759 $\mu m^{6}$, Time Point 2: 17,417$\mu m^{6}$ - 44,068 $\mu m^{6}$, Time Point 3: 5876$\mu m^{6}$ - 24,070 $\mu m^{6}$, Time Point 4: 9923$\mu m^{6}$ - 27,630 $\mu m^{6}$ (units are squared because variance =$sd^{2}$); Cell principal eigenvalue: Time Point 1: 37$\mu m^{2}$ - 214 $\mu m^{2}$, Time Point 2: 9$\mu m^{2}$ - 53 $\mu m^{2}$, Time Point 3: 19$\mu m^{2}$ - 200 $\mu m^{2}$, Time Point 4: 29$\mu m^{2}$ - 218 $\mu m^{2}$; Cell anisotropy: Time Point 1: 0.009 - 0.018, Time Point 2: 0.012 - 0.022, Time Point 3: 0.010 - 0.017, Time Point 4: 0.008 - 0.021.

To investigate the spatiotemporal pattern of local changes in the cell parameters with respect to the neighboring values, a variance analysis was carried out (see cell-level analysis section in STAR Methods). Variance is defined here as difference in the parameter value of a cell with the values for its nine immediate neighboring cells. Overall, variance analysis generated a similar pattern for the different parameters. In Figure 8 the variance patterns of cell volume, principal eigenvalue, and anisotropy are shown. Time Point 1 shows a heterogeneous pattern for the three parameters. At Time Point 2 a ring-shaped region around the outer curvature area was observed in which the cells showed a low level of variance for the cell volume and the principal eigenvalue. Inside the ring in the outer curvature area, the cells showed a high variance for these two cellular parameters. The region with a high variance decreased in area and the ring shaped region around it grew over time. Between the white area and the inner curvature region there was a region with high variance. There was no clear pattern in the inner curvature region. The OFT region mostly had a patchy pattern. Temporally, Time Point 2 showed higher values of variance. The variance for the anisotropy showed, interestingly, a similar but reversed pattern. The ring shaped area exhibited a high level of variance for cell anisotropy and insidethe ring showed a low level of variance. For this parameter, the OFT region also showed a patchy pattern. No pattern was observed for the inner curvature region. The same pattern was exhibited for the cellular data (Figure S16).

### Tissue level deformation displayed a differential directional pattern during C-looping

**Figure 9:**
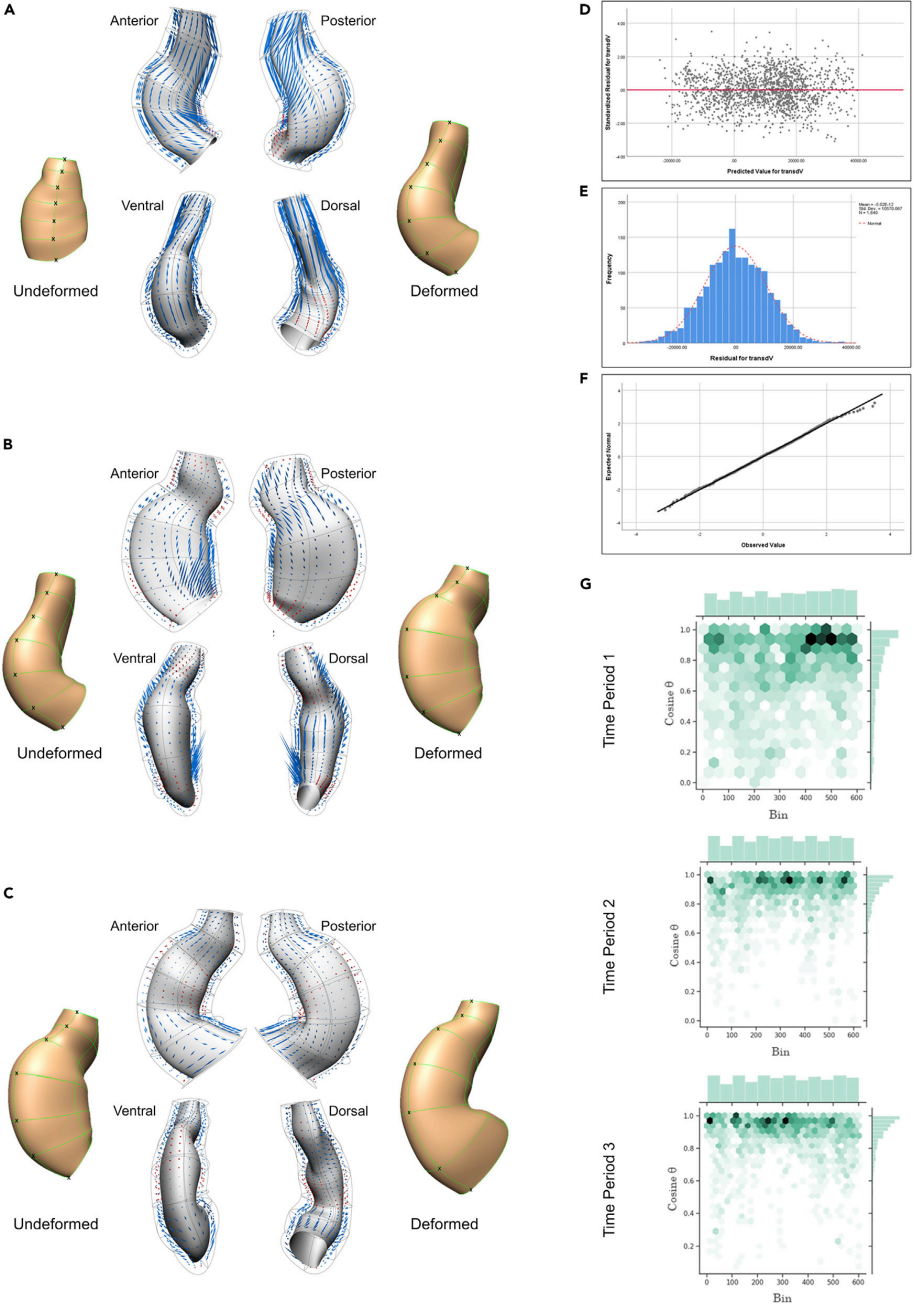
Differential spatiotemporal pattern of tissue is correlated with region-based cellular feature during C-looping (A–C) From a kinematic analysis, tissue deformation was quantified using subsequent temporal geometries. The FE meshes were used to calculate the deformation gradient tensor, F, from the *undeformed* and *deformed* heart states for each two subsequent time points. The magnitude (eigenvalues) and the orientation (eigenvectors) of the deformation calculated from the deformation gradient tensor, F, are displayed on the deformed geometries from Time Point 1 to 2, Time Point 2 to 3, and Time Point 3 to 4, respectively. Each eigenvector and eigenvalue pair is viewed with the mirror-cone glyphs distributed evenly. The length of the glyphs presents magnitude, the inward pointed glyphs in red show shortening and the outward pointed ones in blue indicate elongation. Note that only the eigenvectors associated with the largest eigenvalues are shown. The ventral line of the hearts is labeled by black crosses (see Video S5). (A) During the first time period, where the heart tube shows ventral bending, the entire heart except the dorsal region showed longitudinal elongation with differential magnitude within regions. Although the dorsal region mainly shortened at this time period, the center of bending showed a relatively large circumferential elongation in this region. (B) During the second time period, which accompanies rightward rotation, the OFT region did not show much elongation, and exhibited shortening on both the ventral and dorsal sides. A reversed pattern of deformation was observed in the outer and inner curvature regions compared to the previous time period. Also the anterior and posterior sides of the dorsal region exhibited a significantly different deformation pattern. (C) During the third time period, when the heart forms a C-looped shape, the OFT mostly displayed elongation but the ventral and dorsal regions showed locally diverse patterns of deformation throughout these regions which were different from the previous time period. (D–F) The validation graphs for the fitted General Linear Model, with (d) showing the residual plots for the difference between the observed response and the fitted response values. The plot showed a relatively random scatter pattern around the identity line. Therefore, the residual appears to meet the homogeneity assumption. Both (e) histogram of the residuals and (f) normal Q-Q plot of the residuals for $\text{Δ}V_{tissue}$ showed a normal distribution. (G) Association between changes in the orientation of the cells and the direction of tissue deformation was investigated by calculating the angles between the cell and tissue rotation vectors, and showed using joint plots of bivariate histograms and hexagonal bins. Three time periods are plotted separately. Plots display the histograms of the absolute values of the inner products of the cell and tissue orientation vectors (*y*axis) in the mesh bins (*x*-axis). The hexagon location and intensity illustrates the value and frequency, respectively. The darker shades represents higher frequency. The first time period showed a noticeable smaller correlation than the next two time periods, indicating orientation vectors at cell and tissue levels were not completely aligned. Time periods 2 and 3 show a similar level of alignment. During both time periods there was a relatively close correlation between the cell and tissue vectors with most correlations above the 0.8 level.

The FE meshes were used to calculate the deformation gradient tensor, **F**, from each two subsequent time points. Then, the magnitude (eigenvalues) and the orientation (eigenvectors) of the deformation were calculated from the deformation gradient tensor, **F**. In Figures 9A–9C the deformation is displayed on the deformed geometries from Time Point 1 to 2, Time Point 2 to 3, and Time Point 3 to 4, respectively (see STAR Methods for details and also Video S5). Kinematic analysis of the tissue deformation showed differential deformation pattern at different regions within each time period, but also an altered pattern of deformation at different time periods. From Time Point 1 to 2 (Figure 9A), the OFT region showed a longitudinal elongation with a higher level of stretch on the dorsal side. The ventral region (outer curvature) also showed longitudinal elongation. The magnitude of the stretch was larger in the middle portion of the outer curvature. The dorsal region (inner curvature) mostly showed shortening. However, from the anterior view, there was a region in the center of the bending that showed elongation with a relatively large magnitude in the circumferential direction. (Figure 9B shows deformation from Time Point 2 to 3. At this time period, the OFT region showed both elongation and shortening. The shortening was mainly seen at the conoventricular sulcus (CVS) on both the ventral and dorsal sides. The ventral region (outer curvature) showed no significant lengthening, but did display small shortening at the caudal end. The dorsal region (inner curvature) showed longitudinal elongation along the dorsal line at the cranial end (Figure 9B, dorsal view), and also a larger stretch in the region lateral to the dorsal line on the anterior side (Figure 9B, anterior view). There was a region that showed shortening in the caudal part of the dorsal region (inner curvature), lateral to the dorsal line on the posterior side. (c) Deformation from Time Point 3 to 4. The OFT region exhibited more elongation than shortening in this time period. The elongations were oriented longitudinally. Three distinct regions were observed along the ventral region (outer curvature) (Figure 9C, ventral view). There was a region in the cranial part of the outer curvature that exhibited shortening. In the caudal part there was a region that showed elongation. These two regions were separated by the middle region which showed neither significant elongation nor shortening. The inner curvature showed shortening in the caudal and middle parts, but noticeable longitudinal elongation in the caudal part (Figure 9C, dorsal view). The ventricular region (i.e., all regions in the mesh excluding the OFT region) showed, in general, differentially oriented stretches in the caudal region. In the caudal and middle regions, the ventricular region showed a small amount of shortening, mainly in the outer and inner curvatures, and small elongations mostly in the lateral parts.

Video S5. Tissue dynamics of C-looping, related to Figure 9

### Tissue level deformation is closely linked with changes of cellular features during C-looping

Fitting a General Linear Model revealed the association between tissue growth and cellular features by explaining the volume changes at the tissue level as a function of cellular features (Figures 9D–9F). The final model was:

$$\text{Δ}V_{tissue}\;∼\;Time\;+\;Region\;+\;\text{Δ}N_{cell}\;+\;\text{Δ}V_{cell}\;+\;\text{Δ}A_{cell}\;+\;\text{Δ}V_{ICS}\;+\;Time\;\times \;Region$$

where V,N,A are volume, number, and anisotropy, respectively.

The final model indicated that changes in number and volume of cells as well as in ICS volume and cell anisotropy affect the tissue growth. The introduction of Time and Region improved the fitting result of the model which was indicative of the spatial and temporal effect of the cellular feature on tissue growth. This model explained 70% of the total variation in the tissue volume changes. The details of the model parameter estimates are presented in the Table S19. The correlation analysis between changes in the orientation of the cells with the direction of tissue deformation showed a noticeable smaller correlation for the first time period than the next two time periods. During ventral bending (first time period) orientation vectors at cell and tissue levels were not completely aligned. During time periods 2 and 3 there was, on the other hand, a relatively close correlation between the cell and tissue vectors with most correlations above the 0.8 level (Figure 9G).

## Discussion

We developed an integrative, hybrid experimental and computational workflow to build a comprehensive 3D multi-scale pipeline to link cell level information through to tissue and organ levels in the context of the whole embryo. To test our pipeline, we used four chick embryonic hearts (one per time-point) as a proof-of-concept to perform an extensive region-based, spatiotemporal analysis, to investigate the differential growth hypothesis during C-looping. This study is presented mainly as a method paper to illustrate how these workflows can be integrated to study C-looping. The small number of specimens used here makes it difficult to draw any biological conclusions, and results presented in this paper should be considered as a step toward providing new insights for future studies with a larger number of samples. With differential growth, which has recently attracted interest in the field, C-looping is hypothesized to be controlled by differential growth characteristics of myocardial cells in different regions in the looping heart. We would like to emphasize that this study provides the methodological foundation of the pipeline with potential of extending its components in future studies to investigate the mechanisms involved in C-looping with a larger number of tissue samples.

The experimental workflow of our work generated a 3D, multi-scale dataset from four embryos at different time points during C-looping. This workflow involved a combination of confocal imaging and micro-CT scanning to obtain data from the same embryo at various biological scales. 3D imaging of cell information is crucial to have a comprehensive understanding of the cell organization within the tissue and hence capture more localized changes in cell features with respect to the large-scale alterations in organ shape through development. Therefore, whole-mount confocal imaging was performed. In order to label individual myocardial cells within the developing heart, general cell membrane (WGA), myocardial-specific (NCAM-1), and nuclei staining was carried out and resulted in a multi-channel image of the tissue (Figures 2A–2D). NCAM-1 specific staining of the myocardial layer, as well as no staining in the adjacent layers, including the endocardium, is in agreement with the literature (Crossin and Hoffman, 1991; Thiery et al., 1982). Thiery et al. found the presence of NCAM in the region of the splanchnopleure giving rise to the cardiac primordium and its absence in the endocardium. NCAM remained only in the growing myocardium when the epicardium was formed (Thiery et al., 1982). With the confocal image modality, we successfully extracted information from the cell to organ levels, and exclusively identified myocardial cells from other cells. The resulting images provided single cell scale boundary information while keeping the 3D structure of the heart intact. In addition, using the developed protocol we captured high-resolution data from tissue to the embryonic levels from the micro-CT scanning part of the workflow from the same sample to capture a bigger picture of the heart in the context of the entire organism. Reconstruction of the micro-CT stack resulted in a 3D image of the whole embryo with sub-micron resolution (about 0.5 μm) within the cardiac layer. 3D reconstruction enabled sectioning data in any desired orientation and allowed us to study any preferred slice regardless of angle. In this study, the heart was examined in the context of the whole embryo using this dataset. For example, the level of the rotation of the heart was determined by measuring the angle between the notochord and the heart midline for staging the chicken embryos. According to the literature (Pauwels et al., 2013; Buytaert et al., 2014) and our pilot examination it seemed that high osmolarity of the contrast agent solution seemed to be the cause of the collapse and shrinkage seen in the sample 2i). Because the geometry of the heart could be obtained from the confocal images, no immediate need was found to determine the cause. Embedding an embryo in a hydrogel, which forms stable cross-links with tissue elements, may preserve the heart shape intact and in turn result in less collapse and shrinkage (Wong et al., 2013). The main contribution of this workflow is that it combined two different imaging modalities to obtain data from the same embryo at various biological scales. Confocal imaging provided cell, tissue, and organ level information and micro-CT scanning provided organ and whole embryo level information. Because the two imaging modalities are applied to the same embryo and overlap at the organ level, one should be able to register the two datasets. Getting the organism level information from confocal imaging would be time and resource expensive. Registering the two datasets will provide a comprehensive picture to study the interaction between cellular level changes, heart looping, and whole embryo morphogenesis. We believe that such a dataset is required to gain a comprehensive understanding of the cellular organisation within the tissue, and to capture localized cellular information and large-scale tissue deformation throughout C-looping. To our knowledge, this dataset serves as the first 3D dataset of the C-looping heart that is freely available.

The use of different samples for the study of changes in the geometry limits us in capturing the true dynamics of the morphological changes. An alternative approach is using non-destructive imaging techniques such as optical coherence tomography and ultrasound imaging systems (Gregg and Butcher, 2012; Karunamuni et al., 2014). Because the aim of this study was to develop a pipeline to carry out a multi-biological scale study on the same sample, however, non-invasive imaging was not an option. Therefore, temporal assessment approaches were used. Landmarking within the embryo and heart is a crucial step in this pipeline for registration of the hearts in the space, temporal ordering of the subjects, and, last but not least, defining corresponding nodes and elements at different time points. C-looping has been studied for decades. Labeling experiments and morphological studies have defined landmarks and areas within the developing heart at straight tube and C-looped stages (De la Cruz and Markwald, 1998; Männer, 2000; Moorman and Christoffels, 2003; Okamoto et al., 2010; Rana et al., 2007). Inconsistency in interpretations and terminology, however, makes the landmarking task hard. More work is required to develop solid guidelines for landmarking throughout C-looping. Utilizing a larger number of samples would be advantageous to this part of the workflow. We were aware of the existing challenges and lack of a proper guidelines for staging throughout the C-looping, and therefore, we confirmed our result of the temporal ordering with overall increase in tissue volume and number of cells later from the tissue and cell segmentation approaches.

For the tissue level analysis, the use of the FE method resulted in a successful representation of the geometry of developing hearts. Anatomy is complex; thus the FE method provided a flexible approach to capture the intricacies of the developing heart tissue. The FE representation of the heart anatomy provides a specialized ”material” coordinate system in which both the temporal and spatial dynamics of the C-looping heart are captured. The term material is used because these coordinates effectively identify the positions of any material (i.e., tissue) particle and provide a framework into which the basis of the cell to organ levels linkage can be assembled. In addition, the framework at different time points can be used for a further mechanical and geometrical analysis of the C-looping process. A low RMS error of less than 3 μm was achieved for all subjects indicating a high fitting quality for representing the anatomical geometry of the growing heart.

For the cell level analysis, we successfully extracted and labeled every single myocardial cell from confocal image stacks by implementing a fully automated deep learning algorithm. This dataset provided a true 3D cellular information for the entire heart. The pipeline yielded highly accurate cell segmentation data, comparable in quality to expert manual annotation. Indeed, our method produced a smoother and a more consistent segmentation than a manual segmentation. To the best of our knowledge, this is the first study that has segmented every single myocardial cell in 3D within the whole C-looping heart, thus providing a unique dataset for detailed analysis. The unavoidable adverse effect of the lengthy experimental processes on the cellular features and organization must be acknowledged which may impact the findings; for example, the whole-mount staining of specimen is known to affect the tissue and cellular morphology.

Regional changes during C-looping have been addressed previously for cell shape (Auman et al., 2007; Manasek et al., 1972), differential growth (deBoer et al., 2012; Soufan et al., 2006; Auman et al., 2007), and the ballooning concept (Christoffels et al., 2000). Computational modeling has also revealed that a spatial pattern of cellular growth and shape change is crucial to drive looping (Shi et al., 2014b). Interestingly, a recently published study has suggested that differential growth may not be the main driver and, instead, introduced cell rearrangement as the main factor for looping (Kawahira et al., 2020). However, our region-based study on the OFT, ventral (outer curvature), and dorsal (inner curvature) regions provides evidence in support of the differential growth concept of C-looping. The ventral region consistently showed greater growth of tissue volume and related cellular features compared with the dorsal region at all times. Furthermore, our results provided more detail on the growth-related features of myocardial cells with a higher spatial resolution, and revealed new insights into other cellular mechanisms such as the ICS and feature variance. Importantly, our results also showed distinct patterns of differential cellular features for different time periods corresponding to the individual components of C-looping e.g., ventral bending and rightward rotation.

Our cell volume analysis contradicts the results reported by Kawahira et al. (2020). The source of this inconsistency could be because of different methodological approaches. Firstly, Kawahira et al. (2020)made ellipsoidal estimations for cell volume based on 2D measurements. Secondly, they used only a very small subset of myocardial cells (approximately ≤ 30 cells per region of interest per sample) for their region-based analysis. Lastly, they compared the left and right sides of the heart. In the current study, we made our quantification of the cell volume directly from 3D for all cells (over thousands of cells within the heart). In addition, from a series of extensive regional analyses, we found that the best candidate regions which showed a significant pattern of differential cellular features are the ventral, dorsal, lateral (left and right regions between ventral and dorsal), and OFT regions. In Soufan et al. (2006), it is reported that the ventral region has larger cells than the OFT and the dorsal regions during ventral bending. Similarly, our results also showed that the ventral region has larger cells than the OFT and dorsal regions at Time Point 1 (straight heart tube). At Time Point 2 (ventral bending) and 3 (rightward rotation), the ventral region had significantly larger cells than the dorsal region but not the OFT region. At Time Point 4, where the heart has a C-looped shape, there was no difference in cell size between the three regions. The small difference between the results in our study and those in Soufan et al. (2006) could be because of a difference in the method for measuring the cell volume. Authors in Soufan et al. (2006) calculated average cell volume by dividing tissue volume by the number of cells, and therefore the ICS was not considered separately. In our study, the inner space within cell boundaries and the ICS volume were measured separately. Indeed, including the ICS volume would result in a significant increase in the calculated cell volume. In Soufan et al. (2006), they estimated the maximum cell size to be around 5000 $\mu m^{3}$, whereas the maximum cell size measured in our study was around 400 $\mu m^{3}$ (with outliers at a maximum of 700 $\mu m^{3}$). In another study, the surface area of myocardial cells was measured from scanning electron microscopy images and a mean apical surface area of 50.6 $\mu m^{2}$ in the largest, flattened cells in the outer curvature region was reported (Manasek et al., 1972). If we assume that the cell’s shape is an isotropic cylinder (i.e., height = radius), and the apical surface area represents the area of one circular end of the cylinder, then the volume of these cells would be estimated to over 200 $\mu m^{3}$. This estimate of volume would clearly be larger for elongated cells (i.e., height ¿ radius). It is important to note that, although cell segmentation was performed with high accuracy, there still may be some errors in identifying the boundaries and extracting the cells. Different cell cycles across these regions may be another factor to consider for the observed heterogeneity in cell volumes between the regions. Nevertheless, our method in analyzing cell volume in different regions showed a generally consistent result with those findings in the literature.

One interesting finding in our analysis was the large volume of the ICS between myocardial cells and its notable region-based changes during C-looping. This result is in agreement with previous studies which indicated that the outer layer of myocardial bilayer is an epithelium consisting of tightly coupled cells whereas the inner layer contains significant extracellular space (Manasek, 1968). It should be noted that the measurements in this study are based on myocardial bilayers. We believe that the changes of the ICS volume during C-looping is an imperative factor for the differential tissue growth; however, our small number of samples cannot provide conclusive evidence and it should be experimentally validated in future. Nevertheless, we propose that the role of the extracellular matrix would be a promising subject of further investigation for the C-looping process. For instance, staining ICS with specific labels for extracellular matrix elements could enable further study into the role of the ICS in differential growth during C-looping.

In Manasek et al. (1972) it is reported from a 2D cell shape analysis of myocardial cells in images obtained using scanning electron microscopy that smaller cells were near-randomly distributed in the straight heart tube, and that cell shape showed no significant difference between the right and left side of the pre-looped heart (Manasek et al., 1972) which is consistent with our observation at Time Point 1 where cells did not have a preferred orientation. In the same study they showed that in the post-looped heart cells are larger, flattened, and almost randomly distributed along the right side (the outer curvature) of the heart and that cells are aligned circumferentially along the inner curvature (Manasek et al., 1972). Auman et al. studied myocardial cell shape in Zebrafish as the heart undergoes looping (Auman et al., 2007). They assessed cell shape two-dimensionally and showed that there are relatively small and round cells in the ventricular region of the straight heart tube which increase their surface area throughout looping. Cells in the outer curvature region became elongated and aligned circumferentially, whereas cells in the inner curvature region showed no elongation or orientation (Auman et al., 2007). Qualitatively, cell shape changes in our study were in agreement with the results of Manasek et al. (1972), where the cells were aligned circumferentially in the inner curvature, and also in agreement with the results of Auman et al. (2007), where the cells showed circumferential alignment in the outer curvature. Regional analysis based on statistical methods, however, did not demonstrate a significant pattern. This could be because of the difference between the 2D analyses performed only in a subset of cells locally in those studies and our 3D analysis on all cells in a given region.

Variance of the different cellular parameters, as shown in Figure 8), showed an unexpected and interesting result. Recall that variance is defined here as difference in the parameter value of a cell with the values for its immediate neighboring cells. Absolute values of cell volume, and orientation showed neither spatial nor temporal similarity (Figures 6B–6E). Their variance, however, showed a consistent pattern both within and between time points (Figure 8). A heterogeneous pattern was observed at Time Point 1. However, from Time Point 2 onwards, a ring shape region around the outer curvature area was observed in which cells showed a low level of variance. Inside the ring in the outer curvature, cells showed a region of high variance. This region got smaller over time as the white ring around it grew. This pattern was also visible in the cell level data (Figure S16). The variance pattern for anisotropy showed a reverse pattern i.e., low variance in the outer curvature and high variance in the surrounding ring. This behavior may suggest that, although there is a heterogeneous pattern for cell volume and orientation, cell shapes are more homogeneous in the outer curvature region. The pattern obtained from the variance may be in agreement with the pattern introduced in the ballooning model (Christoffels et al., 2000). According to the ballooning model, the heart tube is composed of primary myocardium that has an embryonic phenotype, including slow growth rate, conduction, and contraction (Christoffels et al., 2000, 2004; Moorman and Christoffels, 2003). At the onset of the looping, a secondary transcriptional program establishes in the outer curvature region to differentiate the primary myocardium into the chamber myocardium (Christoffels et al., 2000). The chamber myocardium, therefore, acquires distinct properties over the primary myocardium including faster proliferation, conduction and, in general, a further differentiation toward a cardiac phenotype (Christoffels et al., 2000, 2004). Christoffels et al. (2000), also showed that the ballooning pattern is maintained during looping, although the domain boundary is not precisely defined and it may evolve.

Now, a question of *how the variance in cellular parameters is related to cellular mechanisms* arises. Variance measures the level of homogeneity and heterogeneity in the cellular population. Higher regional variance (a red region in the outer curvature for cell volume, for instance) is indicative of a greater difference in cell volume in that region, whereas regions with low variance (’white ring’) contain cells that are more similar in size. During development, tissue growth is controlled by regulatory mechanisms which generate specialized cells in order to establish the function of the organ. This organized, highly reproducible, and well patterned process of morphogenesis at the tissue level has been shown to be regulated by stochastic behavior in the underlying cellular and molecular levels (Johnston and Desplan, 2010). How this stochasticity gives rise to reproducible tissue development remains unclear. Recent studies have found that noisy molecular and cellular processes are, indeed, required for tissue development. Stochastic behavior in the subcellular regulatory factors initiates differences between identical cells. Genetic and mechanical feedback loops can then amplify and stabilize these small differences to begin cell differentiation. These first few differentiating cells then trigger classical patterning mechanisms – lateral inhibition for instance – to further trigger cell differentiation and patterning which will then result in a proper and regular developmental pattern (Johnston and Desplan, 2010; Meyer and Roeder, 2014). For example, proliferative responses to neighbor-neighbor differences in Wg signaling (which will induce Yki activity) is suggested to be the primary means by which the Wg gradient drives growth (Zecca and Struhl, 2010). Whether or not this variance pattern is related to the cell differentiation and/or structural and functional patterning during C-looping requires further study.

In addition, we used our dataset to look at the tissue dynamics during the C-looping process. The tissue deformation pattern from kinematic analysis was consistent with the previous results in the literature. Furthermore, our dataset provides more spatial and temporal information on tissue level dynamics. During ventral bending (first time period), the OFT region showed a high level of elongation which is in agreement with the fact that the OFT increases in length during this time period (Männer, 2000). The observed differential pattern of deformation between the ventral region (outer curvature) and the dorsal region (inner curvature), with elongation in former and shortening in latter, is also in agreement with previous studies (Taber et al., 1995). During the second time period, the pattern of deformation changed in all regions. The OFT region, for instance, showed less elongation and instead exhibited shortening locally at both the ventral and dorsal sides. The ventral region (outer curvature) showed no lengthening, but the dorsal region (inner curvature) showed unexpected elongation. This reversed pattern of deformation in the outer and inner curvature regions compared to the previous time period can be explained by the fact that during this time period the heart shows more rotation than ventral bending, whereas, during the previous time point when the heart was bending, there was high level of elongation in the outer curvature region and shortening in the inner curvature region. The asymmetrical pattern of the significant stretch of the anterior side versus posterior side of the dorsal region could also be involved in the rightward rotation. This pattern is in line with a recently reported left/right asymmetry of the tissue deformation (Kawahira et al., 2020). However, our results reveal that there also exists a differential deformation pattern between the ventral and dorsal sides. During further rotation within the third time period, lengthening occurred in the ventral region (outer curvature) but elongation was observed only in the caudal part of the heart tube. The dorsal region (inner curvature) showed shortening in the middle and elongation in the caudal part of the tube. This was a reversal of the pattern observed in the inner curvature region during the previous time period. Altogether, our results provided quantitative region-based information on tissue deformation pattern with spatial and temporal resolution and revealed that different spatial patterns of deformation were observed during C-looping, both spatially within each time point and temporally between time points. It should be noted that the kinematics is not concerned with the cause of the deformation and movement, and does not involve any forces. Also, the deformation gradient tensor, **F**, is a product of both growth deformation and elastic deformation which were not individually distinguishable in this study.

Mapping growth-related cellular features onto the FE meshes from different time points allowed us to investigate the contribution of each feature to growth at the tissue level using a General Linear Model. The final model used all parameters i.e., changes in the number of cells, cell and ICS volumes, and cell anisotropy (please refer to back to the equation in the Results section). Except for the cell anisotropy, the parameters derived from the GLM model were somewhat expected i.e., based on our observations from the significant changes in the number of cells, cell volume, and ICS volume during the looping phase, it is reasonable to see the correlation between these parameters and tissue growth. Cell anisotropy is a measure of shape rather than growth (in terms of number or volume of cells), however, including this parameter improved the model fit. Although it is likely that the cell shape does not contribute to tissue growth *per se*, it may have an association with tissue growth through the relationship between cell shape and the different phases of the cell cycle or, put another way, with proliferation versus differentiation of cells. Including Time and Region in the model significantly improved the overall fit. This provides more evidence that the role of spatiotemporal patterning of cellular features during C-looping should be considered as an important subject of further investigation. Although the final model was able to explain 70% of the total variation in the tissue volume changes, the model did not have enough explanatory power to produce a predictive model as it was not able to explain a significant number of variations. This indicates that other significant factors may be required in the model. This might also be because of the small number of samples in this study. The relationship between changes in the orientation of cells and the tissue deformation direction was also analyzed in this study. The results showed a smaller relationship during the first time period where the straight heart tube bends ventrally. During the next two time periods, when the heart rotates rightward, a stronger association was observed. This difference indicated that cell orientation could likely contribute more to the rotational rather than bending component, which is in agreement with previous postulations that bending and rotational components of C-looping are regulated by different mechanisms (Shi et al., 2014a). It is perhaps worthwhile to mention that the correlations observed from the GLM analysis do not show any causality between the parameters and tissue growth. For example, heart tube deformation in response to other extrinsic factors may result in changes in cellular features because of tissue stress. The observations in this study, however, can provide a basis for future experimental studies to test various hypotheses about the relationship between these cell-level features and overall tissue dynamics. Please see Table S19 in the supplemental information for an example of the coefficient values of the final GLM model.

### Limitations of the study

The main limitation of this study was the number of samples at each time point. Adding more subjects into this study for each time point could potentially lead to a more comprehensive analysis and outcome, and provide stronger biologically relevant conclusions. The collapse of the heart and shrinkage of the sample during preparation for micro-10.13039/100004811CT should be further investigated and resolved by perhaps embedding the embryo within a block of hydrogel to help maintain the integrity of the sample by providing a more solid support. In this study the mechanical aspects of C-looping were not fully investigated. The generated meshes at different time points using FE methods can be used for a further mechanical and geometrical analysis of C-looping process. Also, in the cell-tissue correlation analysis, any elastic deformation was excluded from the deformation gradient tensor, **F**, and it was assumed that the tissue volume changes were a result of growth only. Including elastic deformation in the model will, indeed, improve the analysis accuracy and should be considered for future work. Last, but not least, the entire workflow presented in this study could be applied to other model organisms such as mouse and rat for a comparative study on the mechanism of C-looping.

## STAR★Methods

### Key resources table

**Table:** 

REAGENT or RESOURCE	SOURCE	IDENTIFIER
**Antibodies**		

Donkey serum	Sigma-Aldrich	Cat# D9663; RRID:AB_2810235
NCAM-1 antibody (5e)	DSHB	https://dshb.biology.uiowa.edu/5e
donkey anti-mouse biotin	Thermo Fisher Scientific	Cat# A16015; RRID:AB_2534689

**Chemicals, peptides, and recombinant proteins**		

Wheat Germ Agglutinin, Alexa Fluor® 488 conjugate	Thermo Fisher Scientific	Cat# W11261
4′,6-diamino-2-phenylindole (DAPI)	Thermo Fisher Scientific	Cat# D1306
Streptavidin, Alexa Fluor 568 conjugate	Invitrogen	Cat# S-11226
Phosphotungstic Acid (PTA)	Sigma-Aldrich	CAS# 12501-23-4

**Deposited data**		

A method for investigating spatiotemporal growth patterns at cell and tissue levels during C-looping in the embryonic chick heart, a dataset containing original image stacks, preprocessed images, segmented files, postprocessed images and data, spreadsheets, relevant run-able scripts and codes for producing the visualisations and figures.	Mendeley Data	https://doi.org/10.17632/jwj6m5yxct.1; https://doi.org/10.17632/hww6c4yvhp.1; https://doi.org/10.17632/fckszv3tp3.1; https://doi.org/10.17632/wc3yc9rh7h.1

**Experimental models: Organisms/strains**		

Fertilised Cobb Broiler brown eggs	Bromley Park Hatcheries LTD	https://www.bromley.co.nz/

**Software and algorithms**		

ZEISS ZEN software	Zeiss	RRID:SCR_013672
Fiji-imageJ	National Institute of Health	RRID:SCR_002285
InstaReconCBR software	Bruker Corporation	http://instarecon.com/cbr-product/
SkyScan DataViewer software	Bruker Corporation	https://www.bruker.com/en/products-and-solutions/preclinical-imaging/micro-ct/3d-suite-software.html
SkyScan CTVox software	Bruker Corporation	https://www.bruker.com/en/products-and-solutions/preclinical-imaging/micro-ct/3d-suite-software.html
Amira	Thermo Fisher Scientific	https://www.thermofisher.com/au/en/home/electron-microscopy/products/software-em-3d-vis/amira-software.html
OpenCMISS	ABI	http://opencmiss.org/
CMGUI	ABI	http://physiomeproject.org/software/opencmiss/cmgui/download
Python Programming Language	Python Software Foundation	RRID:SCR_008394
IBM SPSS Statistics	IBM Corp.	RRID:SCR_019096

### Resource availability

#### Lead contact

Further information and requests for resources and reagents should be directed to and will be fulfilled by the lead contact, Nazanin Ebrahimi: n.ebrahimi@auckland.ac.nz.

#### Materials availability

This study did not generate new unique reagents.

### Experimental model and subject details

This study used chicken as the model organism. Fertilised Cobb Broiler brown eggs were purchased from Bromley Park Hatcheries LTD (Tuakau, New Zealand). The eggs were stored in the fridge at $10^{\circ }$C and 50% humidity to pause the initiation of embryo development, before incubating them at $37^{\circ }$C and 48% humidity in a forced draft incubator. The eggs were incubated between 36 and 46 hours to harvest embryos between Hamburger-Hamilton stages 10 and 11 (Hamburger and Hamilton, 1951).

Upon removing an egg from the incubator, it was placed on a custom-made egg holder. A small hole was made in one end of an egg using a mini hand drill. Using a syringe and needle, 2ml of albumin were removed to lower the embryo, which resulted in the embryo and its extra-embryonic membranes getting detached from the top of the shell. Another small hole was drilled on top of the egg, and a drop of saline solution was placed on the hole to moisten the shell membrane and to wash shell dust away. A pair of forceps was used to cut a window (approximately 20−30mm in diameter) out of the shell. The egg on the egg holder was then placed under a dissecting microscope for harvesting.

A pair of micro-scissors was used to cut all around the ‘area opaca’. The embryo was transferred using a mini perforated spoon into a small Sylgard coated Petri dish containing the saline solution and pinned on Sylgard using micro pins. The saline was replaced with freshly made fixative (4% para-formaldehyde in 0.1M Phosphate Buffer (PFA), pH 7.4). The Petri dish was left in a ventilated fume hood at room temperature for the first 24h. The embryo was then transferred to glass bottles with fresh fixative, rocking at 4°C for approximately another 24 hours. The embryos were then washed in Phosphate Buffered Saline (PBS) 0.01M and stored in PBS with 0.01% sodium azide at $4^{\circ }$C. Fresh and fixed embryos were imaged using a dissecting microscope (Leica M205 FA; Leica) to backtrack the changes in the size and shape of embryos and hearts during preparation processes.

### Method details

#### Experimental workflow

This section provides the experimental methods developed in this study to generate a series of a characteristic 3D datasets from individual chicken embryos from cell level resolution to the whole organism level. The pipeline uses two imaging modalities: 3D confocal imaging and micro-CT scanning.

##### Whole-mount confocal imaging of chick embryonic heart

In order to get a 3D image of the whole C-looping heart in which individual cells are labelled, immunocytochemistry was performed and coupled with a streptavidin-biotin system for signal amplification. Fixed embryos were used for whole-mount triple labelling to stain the nuclei and cell membranes of all cells within the embryo, and also the cell membrane of myocardial cells within the heart. DAPI, a popular nuclear stain, was used to stain nuclei within the embryos. Wheat Germ Agglutinin (WGA) was used as a general cell membrane stain. WGA is a lectin that has a high affinity for N-acetylglucosamine residues on cell membrane glycoproteins. 5e (deposited to the Developmental Studies Hybridoma Bank by U. Rutishauer) was used to stain myocardial cell membrane in chicken embryos. 5e, which is a monoclonal antibody against the extracellular domain of neural cell adhesion molecule (NCAM), has been shown to be transiently expressed in the developing chicken heart (Crossin and Hoffman, 1991; Thiery et al., 1982). 5e will be referred to as NCAM-1 in this paper. Also, in order to amplify the signal quality we used the streptavidin-biotin system.

##### Whole-mount fluorescent staining in chick embryos

The fixed embryos were washed in PBS prior to blocking with blocking solution overnight at room temperature. The blocking solution was made of 10%normal heat-inactivated donkey serum (Sigma-Aldrich) and 0.05% Triton X100 in PBS with sodium azide. The embryos were then incubated with the primary antibody, NCAM-1 (1:5, 5e supernatant) in a primary diluting solution (5%normal heat-inactivated donkey serum and 0.05% Triton in PBS with sodium azide) for at least 24 hours at $4^{\circ }$C. After that, the samples were washed with PBS three times, for an hour and a half for each wash. The embryos were incubated with donkey anti-mouse biotin (1:200, Thermo Fisher Scientific) in a secondary diluting solution (5%normal heat-inactivated donkey serum in PBS) overnight, followed by three washes with PBS as before. The embryos were then incubated with streptavidin conjugated with Alexa*TM* 568 (1:1000, Invitrogen) overnight. Incubation with streptavidin and biotin was repeated after washing the samples in PBS (as before) in order to further amplify the signal. However, the incubation and washing times were halved this time. Staining with streptavidin-biotin and washing steps were performed at room temperature (RT) with agitation. The samples were then stained with Alexa Fluor*®* 488 conjugate of WGA (10 μg/ml, Thermo Fisher Scientific) for about two days at RT with agitation. DAPI (1 μg/ml, Thermo Fisher Scientific) was added to the samples after washing WGA three times with PBS for about one hour for each wash. The samples were left in DAPI solution for 3−4 hours followed by washing with PBS as before.

##### Optical clearing of whole embryo

After whole-mount staining, the embryos were serially incubated in a 25%, 50%, 75%, and 90% solution of Glycerol in PB/4% PFA, for 1−2 hours for each step. The degree of clarity was monitored under a dissecting microscope.

##### Mounting the whole embryo for confocal imaging

In order to mount the embryo for the confocal imaging, a chamber was made on regular microscope slides using polystyrene sheets with holes. The embryos were then transferred into the chamber and mounted in Citifluor with the heart facing up and covered with a microscope coverslip. The depth of the chamber was adjusted for each embryo in order to place the coverslip as close as possible to the heart with minimal space between the coverslip and the specimen. The mounted samples were imaged on the same day or next.

##### Confocal image acquisition

All images were obtained using a Zeiss LSM 710 inverted confocal microscope (The University of Auckland Biomedical Imaging Research Unit (BIRU).) with a 25× multi-immersion objective (Zeiss, LD LCI Plan-Apochromat, NA = 0.8, WD =600μm). Glycerol was used as an immersion solution to match the refractive index with the mounting solution. An argon laser (488nm) was used for visualising Alexa Fluor 488. The Alexa Fluor 488 conjugate of WGA exhibited the bright green fluorescence of the dye. DAPI was excited with a diode laser (405nm) and resulted in a blue fluorescence. Alexa Fluor 568 conjugated to the streptavidin was visualised with a diode-pumped solid state laser (561nm). Alexa Fluor 568 exhibited a bright orange fluorescence. The microscope software, ZEN, was used to control microscope components to acquire 3D image stacks with the following settings: 1 AU (airy unit) Pinhole; 0.7μs Pixel Dwell; 4656×4656 Frame Size; 0.12μm Pixel Size; 1.12μm Interval (Z-step Size); 4 times averaging; 0.6× zoom. The chicken embryo hearts could not fit into the field of view of the microscope with a 0.6× zoom. Therefore, to obtain an image that covers the entire heart, the tiling feature of the microscope was used. The hearts were imaged in tiles with 15% overlap.

##### Confocal image reconstruction

In order to generate a super-image of the whole chicken heart, image tiles were stitched together. Basic image preparation including resizing was performed in Fiji-imageJ (ImageJ 1.52i, https://imagej.nih.gov/ij/). Fiji is an open source image processing package based on ImageJ which bundles a number of plugins for image analysis. Stitching was done using the Grid/Collection Stitching Plugin in Fiji. The Grid/Collection Stitching Plugin allows several tiles placed in varying dimensions to be stitched together. Stitching resulted in the reconstruction of the 3D image of the chicken hearts.

##### Sample preparation, staining, and embedding for micro-CT imaging of whole embryo

In order to capture information on the tissue to organism levels from the same sample, the embryos scanned with confocal microscopy were used for micro-CT scanning. After confocal imaging, the embryos were taken out of the microscope slide and washed in PBS overnight with agitation at RT. To ensure tissue integrity, the embryos were transferred to the fixative solution (PFA) for another 24 hours before further preparation. The embryos can be stored in PBS at $4^{\circ }$C at this stage. The embryos were then stained with Phosphotungstic Acid (PTA, Sigma-Aldrich). PTA is a contrast agent which is widely used for embryo staining in micro-CT imaging. The PTA staining was carried out according to the published protocol with slight modifications (Metscher, 2011). Each embryo was immersed in an alcoholic PTA solution (0.3% PTA in 70% Ethanol) for about 15 hours with agitation at RT. The samples were then washed in PBS three times, each wash for about an hour, before embedding.

The embryos were embedded in cocoa butter within a 4mm straw mounted on the sample chuck. Three layers of Mylar were applied onto the outside of the straw. Mylar is an aluminised polyester film made from stretched polyethylene terephthalate (PET). It is shown that Mylar tape helps to reduce the radiated heat from the X-ray source which does result in sample movement and/or sample shape change. It also acts as a thin aluminium filter which modifies the detected X-ray energy (Gerneke et al., 2017).

##### Micro-CT sub-micron image acquisition

3D datasets were acquired for each embryo using a micro-CT scanner (Skyscan 1272, Bruker) (The Auckland Bioengineering Institute MicroCT and Imaging Centre). The specifications of this scanner have been fully described (https://www.bruker.com/products/microtomography/micro-ct-for-sample-scanning/skyscan-1272/overview.html). The instrument settings were as follows: 40- 60kV, 160- 250mA, and 4904×3280 camera pixels. No filter was used inside the instrument, however Mylar applied around the sample tube acted as a very thin filter with about 120μm thickness. The embryos were imaged twice at 0.5- 0.6μm and 1.4- 1.6μm to image hearts and the whole embryos, respectively. The whole chicken embryos were imaged at lower resolution due to the size of the embryo that could not fit in the field of view. The embryos were scanned with a 0.2 degree for rotation steps over 180 degrees, 2 for frame averaging and 4 pixels for random movement.

##### Micro-CT image reconstruction

The acquired 3D datasets were reconstructed using InstaRecon ®CBR software. Firstly, X/Y Alignment With a Reference Scan algorithm was used to minimise thermal drift of the sample which affects the actual image resolution. Then, the 3D volume dataset was reconstructed. For the reconstruction, values for smoothing, ring artefact reduction, and beam-hardening correction were adjusted for individual samples. After that, the reconstructed images were loaded in the SkyScan DataViewer software to view the data with respect to the overall image, reorient, crop, and resave the dataset in the desired orientation. The SkyScan CTVox software was used for volume rendering of the image stacks and making movies.

#### Computational workflow

This section provides methods used for analysis of the obtained experimental data. This analysis relies on a pipeline consisting of different steps for data preparation, processing and quantitative analysis and utilises a variety of software, tools, scripts, and standard statistical approaches.

##### Datasets

To study the C-looping heart, four chicken embryos were selected out of 90 harvested subjects (samples S1 to S4 in Figure 3A). The selection was based on the preliminary examination under a dissecting microscope. Defining the exact developmental stage of the hearts and choosing sequential time points was not possible under a dissecting microscope. C-looping is a fast, dynamic process, and harvesting several samples from such a narrow temporal window is hard. There are variations in growth rate, therefore incubation time cannot be used for staging. Thus, approximate staging was carried out using the gold-standard papers in the literature to choose those samples that were within the C-looping window by examination under a dissecting microscope for further analysis (Hamburger and Hamilton, 1951; Männer, 2000). The confocal and micro-CT imaging datasets were obtained for each sample using the pipeline developed in the experimentation part of this paper.

##### Geometric data

As with any other image analysis and modeling, the first step was to segment the heart region from other areas in the image. Confocal image stacks were used for heart segmentation because they provided a more appropriate and undistorted image of heart geometry compared with micro-CT images. A thorough analysis from all of the images throughout the experiments - from the harvesting stage all the way to the post-imaging stage - showed that some areas in the heart tissue were collapsed in the micro-CT images (see Figure 2I) whereas images from the confocal microscopy showed the hearts to be unchanged.

Segmentation was carried out using Amira™ Software (version 6.5.0, Thermo Fisher Scientific). In Amira, a semi-automatic workflow using a region growing algorithm, morphological operations, and manual segmentation was developed with accuracy to maintain the integrity of structural features and landmarks whereas continuing to produce smooth boundaries. More details on the workflow used in Amira are provided below.

##### Segmentation workflow in Amira

Amira is a powerful, multifaceted platform for 2D/3D visualization, image enhancement, segmentation and extensive analysis. It has a modular and object-oriented system where modules are used to visualize data as objects and/or to perform computational operations on them. The outcome of module computation is stored in a new file. Depending on the data object, Amira provides a large variety of advanced image processing and quantification algorithms and tools which can be applied to the existing data.

The general algorithm developed in Amira for segmentation of the heart area consisted of four main steps: (1) Extracting all of the regions including myocardial, endocardial, and splanchnopleure layers, and other surrounding tissues from the image stack in which WGA and NCAM-1 signals were combined. (2) Identifying and extracting the myocardial layer using the NCAM-1 channel. Note that the thickness of the myocardial layer from this channel was thinner than the actual thickness since NCAM-1 did not stain the outer surface of the cell membrane where cells were facing the pericardial coelom or the cardiac jelly (see Figure 2B). (3) Applying morphological operations to the segmented myocardial layer to fill in holes and dilate the layer. (4) Using the dilated myocardial layer as a mask to extract the myocardial region with realistic thickness from the result of step (1). The confocal 3D stack of stitched images of the heart was loaded in Amira and visualized as a multi-dimensional dataset. For heart region segmentation, channels 1 and 3 were used which represented WGA and NCAM-1 stains, respectively. Each channel was re-sampled using the *Resample* module to produce cubic voxels of $0.24\times 0.24\times 0.24\mu m^{3}$. Channels were then smoothed using a *Median Filter*. In order to have complementary signals for the cell membrane of myocardial cells, the *Add Image* module was used. This module performs a logical AND operation for each voxel between two images and stores the result as a new volume.

The resulting combined image was opened in the *Segmentation Editor* environment. The *Segmentation Editor* provides a user-friendly interface in which an interactive segmentation can be performed using different tools. The *Magic Wand* tool was used to perform a 3D region growing operation on the combined image stack. It was set to grow the region from a seed point, a manually placed site within the area with signal, and to include all voxels with values inside the user-defined values for the masking range. The *Segmentation Editor* provides a multi-viewer window, therefore, the signals can be viewed at all three orthogonal planes at the same time as the 3D rendered volume (Figure S2). This process resulted in a labeled image in whichall the layers in the image were segmented (WGA + NCAM-1 column in Figure S3). In order to segment the heart area, the same tool (*Magic Wand*) was used to segment signals from channel 3 which resulted in another labeled image with only the myocardial layer segmented in it (NCAM-1 column in Figure S3). This segmented layer was used as a clue to separate the myocardial layer from other parts. In the *Project* environment, the label file of myocardial segmentation from channel 3 further operated with morphological *Closing* and *Dilation* modules to fill in holes and dilate the label to be used as a mask. Then, segmentation from the combined image was masked with the dilated myocardial layer using the *Mask* module. This module provides a masking of the combined image with a binary mask of the dilated myocardial layer and stores the result as a new binary image of the segmented heart region.

Having a myocardial specific channel was extremely useful to distinguish the myocardial regions from surrounding areas. For example, in Figure S3, the top panel shows a section from the middle part of the heart. It may be possible to define the heart boundary if one is familiar with the anatomy and histology. However, without the NCAM-1 signal (WGA column) or a combined image of WGA and NACM-1 signals (WGA + NCAM-1 column), it is a difficult task to identify the heart boundaries in the bottom panel, which shows the top part of the heart. Masking (WGA + NCAM-1 column) with (NCAM-1 column) will result in a binary image of the myocardial layer mostly isolated from non-myocardial areas. Manual cleaning was performed in the *Segmentation Editor* using the *Lasso* and *Brush* tools to clean all non-myocardial areas; for example, in the areas where the epicardium was very close to the myocardial layer. The *Lasso* enables the user to generate a closed freehand contour around an area to select it. Using the *Brush*, one could select the region by brushing voxels in 2D. The selected areas by the *Lasso* or *Brush* could be deleted from or added to the label field image.

The final image represented a precise geometry of the heart for all of the subjects in a consistent, accurate, and reliable manner. Figure 3B shows one of the samples in which the heart is perfectly segmented from the adjacent layers.

##### Anatomical landmarking

To find the boundaries of the heart area and define the corresponding points and regions at different time points, anatomical landmarks had to be defined within each heart. Fate mapping and lineage studies (including labeling experiments and time-lapse imaging) in the looping heart have provided evidence on the origin and fate of the given areas within the heart (De la Cruz and Markwald, 1998; Moorman and Christoffels, 2003; Rana et al., 2007; Okamoto et al., 2010). However, there is inconsistency in the interpretation and resulting proposed models in the field (Männer, 2000). In addition, the considerable diversity in the use of the nomenclature (see (Arráez-Aybar et al., 2003)) of the looping heart results in more confusion when interpreting and using these models.

In this study, the corresponding areas were defined mainly based on the model reviewed and illustrated in Okamoto et al. (2010). The areas defined within each heart in this study were the aortic sac (AS), primitive outlet (PO) which divided into the truncus (T) and conus (C) at Time Point 2 when the conoventricular sulcus appeared, primitive right ventricle (PRV), primitive left ventricle (PLV), primitive atrium (PAT), and primitive atrioventricular canal (PAVC) which appeared at Time Point 3. The left and right lateral furrows were used to define the boundaries between these areas including trunco-conal curvature (TCC), conoventricular sulcus (CVS), interventricular sulcus (IVS), atrioventricular sulcus (AVS) (Figure 3F).

The ventral midline of the heart tube was marked by the points where the endocardium layer was in touch with the myocardium (Shi et al., 2014b). Indeed, the endocardium showed a flattened ellipsoidal lumen which was used as the reference for the ventral and dorsal line of the heart tube (Figure 3E)

##### Heart volume sampling

Once the myocardial layer was segmented, a 3D spatial representation of the heart was required for fitting. Because the middle region of the heart (ventricular region) is of interest in this project, other irrelevant regions including the inlets were excluded in order to keep the model simple. The inlet areas were deleted manually in Amira.

Next, the *Generate Surface* module in Amira was employed to render a digitized, volumetric geometry from segmented heart. From this, a 3D point cloud was generated using the *Point Cloud* module. The point cloud was further down-sampled in order to keep the data file reasonably small for analysis. However, as far as shape and geometry were concerned, this down-sampling should not impact the reconstruction error because these data were later used to fit a template finite element (FE) mesh to describe the heart shape.

##### Volume mesh construction

The 3D meshes used in this thesis to describe the heart shape are finite element (FE) volume meshes. The FE method uses interpolation to find a solution for field values at a number of points in a given domain of interest, and approximate the found solution between these points. These points are known as *nodes* which are connected through a subregion of the domain called an *element*. *Basis functions* provide an interpolating function in the FE method to map the field coordinate and global nodes onto the local parametric coordinates, ξ. These local element coordinates range from 0 to 1.

Volume meshes have nodes and elements not only on the surface of the geometry but contain nodes inside the geometry (i.e., an additional dimension). The shape function is derived from the tensor product of 1D interpolation functions. The 3D counterparts can then be inferred from 1D functions. The element type presented in this study is tricubic Hermite where all three directions are interpolated with cubic Hermite functions. The four 1D cubic Hermite basis functions are given by (Equation 1).

(Equation 1) $$\begin{array}{l} \psi_{1}^{0}\left(\xi \right)=1-3\xi^{2}+2\xi^{3}\\ \psi_{2}^{0}\left(\xi \right)=\xi^{2}\left(3-2\xi \right)\\ \psi_{1}^{1}\left(\xi \right)=\xi {\left(\xi -1\right)}^{2}\\ \psi_{2}^{1}\left(\xi \right)=\xi^{2}\left(\xi -1\right) \end{array}$$

where *ξ* is the local coordinate to each element. The interpolation of the global coordinates within each element is given by

(Equation 2) $$u\left(\xi \right)=\psi_{1}^{0}\left(\xi \right)u_{1}+\psi_{1}^{1}\left(\xi \right){\frac{du}{d\xi }|}_{1}+\psi_{2}^{0}\left(\xi \right)u_{2}+\psi_{2}^{1}\left(\xi \right){\frac{du}{d\xi }|}_{2}$$

where **u** is *x*, *y*, or *z* of a rectangular Cartesian (RC) coordinate system and $\frac{du}{d\xi }$ is the derivative of the global coordinate with respect to the local coordinate ξ. Subscripts refer to node numbers (node 1 and node 2) and superscripts refer to the derivatives (derivative 0 and derivative 1).

To provide $C^{1}$ continuity across the element boundaries in cubic Hermite elements, 24 parameters (degrees-of-freedom) are required to be defined for each node. These parameters are the nodal values (x, y, and z) as well as surface curvature information as the first-order, second-order, and third-order derivatives $\left(\frac{\partial n}{\partial \xi_{1}},\;\frac{\partial n}{\partial \xi_{2}},\;\frac{\partial^{2}n}{\partial \xi_{1}\partial \xi_{2}},\;\frac{\partial n}{\partial \xi_{3}},\;\frac{\partial^{2}n}{\partial \xi_{1}\partial \xi_{3}},\;\frac{\partial^{2}n}{\partial \xi_{2}\partial \xi_{3}},\;\frac{\partial^{3}n}{\partial \xi_{1}\partial \xi_{2}\partial \xi_{3}}\right)$ where *n* is *x*, *y*, or *z*, and ξ is the local element coordinate.

A problem arises when two adjacent elements have unequal lengths. Therefore, the derivative ${\frac{du}{d\xi }|}_{\alpha }$ at local node *α* which is dependent on the local coordinate *ξ* will be different and, in turn, discontinuous in the adjacent element. In order to achieve derivative continuity throughout the mesh, it is better to base derivative interpolation with respect to a physical property. The derivatives are, therefore, expressed based on the arc-length parameter $\frac{du}{ds}$, at nodes which is given by

(Equation 3) $${\frac{du}{d\xi }|}_{\alpha }=\frac{du^{\text{Δ}\left(\alpha ,e\right)}}{ds}{\left(\frac{ds}{d\xi }\right)}_{e}=\frac{du^{\text{Δ}\left(\alpha ,e\right)}}{ds}S^{e}$$

where $\frac{du}{ds}$ is the partial derivative with respect to arc-length, Δ(α,e) is the node number in the global coordinate for the local node *α* in element *e*, and ${\left(\frac{ds}{d\xi }\right)}_{e}$, denoted by $S^{e}$, is the element arc-length scale factor by which the partial derivative is scaled. This leads to partial derivatives, $\frac{du}{ds}$, to be relatively continuous across element boundaries. The cubic Hermite interpolation formula in (Equation 2) now becomes

(Equation 4) $$u\left(\xi \right)=\psi_{1}^{0}\left(\xi \right)u_{1}+\psi_{1}^{1}\left(\xi \right){\frac{du}{d\xi }|}_{1}S^{e}+\psi_{2}^{0}\left(\xi \right)u_{2}+\psi_{2}^{1}\left(\xi \right){\frac{du}{d\xi }|}_{2}S^{e}$$

In this new formula, the interpolation is based to be with respect to *s* rather *ξ*. However, the scale factor will affect changes of *ξ* with s. The parameter *s* is hence chosen to be the arc-length along the element line to have a nearly uniform change of *ξ*, with *s*. It is called *arc-length scaling*. The arc-length is defined by

(Equation 5) $$s=\int_{0}^{1}\sqrt{{\left(\frac{dx}{d\xi }\right)}^{2}+{\left(\frac{dy}{d\xi }\right)}^{2}+{\left(\frac{dz}{d\xi }\right)}^{2}}d\xi$$

To calculate the arc-length, (Equation 3) should be solved iteratively in conjunction with (Equation 4), since the interpolation function uses the arc-length to calculate the scale factor in (Equation 4), and this equation is a function of the scale factor. The arc-length equation above results in a unit magnitude arc-length derivative at each node *n* which ensures there is a uniform *ξ* change along the global arc-length, i.e.,

(Equation 6) $$\left||{\left(\frac{du}{ds}\right)}_{n}|\right|=1.$$

Finally, to ensure $C^{1}$ continuity, the *ξ* directions should be consistent in adjacent elements but also, the scale factor at a node must be the same for the node in another element. Therefore, the scale factors must be based nodally.

##### Initial template mesh

Because the heart resembles a tube that undergoes simple bending during the early stage of development, a cylindrical topology was used to construct the mesh template. The number of elements and nodes were kept to a minimum (i.e., a coarser mesh). This would help with speeding up the fitting process (explained later in this STAR Methods section) and keeping the geometry simple while still maintaining the accuracy of the fit. The mesh contained 28 external nodes, 28 internal nodes, and 24 elements. This resulted in a 56-node-24-element tube model for PO/OFT and primitive ventricular region of heart, formulated in a global RC coordinate system. Local coordinates in this tube model are defined so that $\xi_{1}$moves circumferentially, $\xi_{2}$ travels longitudinally, and $\xi_{3}$ goes through the heart. Because the tube mesh is open at both ends and there is no collapsing at any parts of the mesh, no nodal derivative versions were required to be introduced at the nodes.

##### Volume fitting of the heart geometry

The fitting method used in this study is a linear least squares optimization and is performed using the software package OpenCMISS (Bradley et al., 2011). The method has previously been demonstrated (Bradley et al., 1997; Fernandez et al., 2004). To begin the fitting process, data points must be projected onto the nearest surface of the initial mesh. The projection of a data point is obtained when the normal distance between the data point and its projection on the mesh is found (Figure S4). The global coordinates of the projected points are a function of the local element coordinates, $\xi_{1}$and $\xi_{2}$, and nodal parameters, $u_{n}$. A least-square distance function, *D*, between a data point in the spatial coordinate, $z_{d}$, and its projection onto the mesh is given by

(Equation 7) $$D\left(\xi_{1},\xi_{2}\right)={\left\|u\left(\xi_{1},\xi_{2}\right)-z_{d}\right\|}^{2},$$

where $u\left(\xi_{1},\xi_{2}\right)$ is interpolated using

(Equation 8) $$u\left(\xi_{1},\xi_{2}\right)=\psi_{1}^{0}\left(\xi_{1}\right)\psi_{1}^{0}\left(\xi_{2}\right)u_{1}+\psi_{2}^{0}\left(\xi_{1}\right)\psi_{1}^{0}\left(\xi_{2}\right)u_{2}+\;\psi_{1}^{0}\left(\xi_{1}\right)\psi_{2}^{0}\left(\xi_{2}\right)u_{3}+\psi_{2}^{0}\left(\xi_{1}\right)\psi_{2}^{0}\left(\xi_{2}\right)u_{4}+\psi_{1}^{1}\left(\xi_{1}\right)\psi_{1}^{0}\left(\xi_{2}\right){\left(\frac{\partial u}{\partial \xi_{1}}\right)}_{1}+\psi_{2}^{1}\left(\xi_{1}\right)\psi_{1}^{0}\left(\xi_{2}\right){\left(\frac{\partial u}{\partial \xi_{1}}\right)}_{2}+\psi_{1}^{1}\left(\xi_{1}\right)\psi_{2}^{0}\left(\xi_{2}\right){\left(\frac{\partial u}{\partial \xi_{1}}\right)}_{3}+\psi_{2}^{1}\left(\xi_{1}\right)\psi_{2}^{0}\left(\xi_{2}\right){\left(\frac{\partial u}{\partial \xi_{1}}\right)}_{4}+\psi_{1}^{0}\left(\xi_{1}\right)\psi_{1}^{1}\left(\xi_{2}\right){\left(\frac{\partial u}{\partial \xi_{2}}\right)}_{1}+\psi_{2}^{0}\left(\xi_{1}\right)\psi_{1}^{1}\left(\xi_{2}\right){\left(\frac{\partial u}{\partial \xi_{2}}\right)}_{2}+\;\psi_{1}^{0}\left(\xi_{1}\right)\psi_{2}^{1}\left(\xi_{2}\right){\left(\frac{\partial u}{\partial \xi_{2}}\right)}_{3}+\psi_{2}^{0}\left(\xi_{1}\right)\psi_{2}^{1}\left(\xi_{2}\right){\left(\frac{\partial u}{\partial \xi_{2}}\right)}_{4}+\psi_{1}^{1}\left(\xi_{1}\right)\psi_{1}^{1}\left(\xi_{2}\right){\left(\frac{\partial^{2}u}{\partial \xi_{1}\partial \xi_{2}}\right)}_{1}+\psi_{2}^{1}\left(\xi_{1}\right)\psi_{1}^{1}\left(\xi_{2}\right){\left(\frac{\partial^{2}u}{\partial \xi_{1}\partial \xi_{2}}\right)}_{2}+\;\psi_{1}^{1}\left(\xi_{1}\right)\psi_{2}^{1}\left(\xi_{2}\right){\left(\frac{\partial^{2}u}{\partial \xi_{1}\partial \xi_{2}}\right)}_{3}+\psi_{2}^{1}\left(\xi_{1}\right)\psi_{2}^{1}\left(\xi_{2}\right){\left(\frac{\partial^{2}u}{\partial \xi_{1}\partial \xi_{2}}\right)}_{4}$$

and the converted first-order and second-order derivatives with respect to *ξ* are given by the following Equations, 9 and 10, respectively:

(Equation 9) $$\left(\frac{\partial u}{\partial \xi_{\alpha }}\right)\;=\;\left(\frac{\partial u}{\partial s_{\alpha }}\right)\left(\frac{\partial s_{\alpha }}{\partial \xi_{\alpha }}\right)$$

(Equation 10) $$\left(\frac{\partial^{2}u}{\partial \xi_{\alpha }\partial \xi_{\beta }}\right)\;=\;\left(\frac{\partial^{2}u}{\partial s_{\alpha }\partial s_{\beta }}\right)\left(\frac{\partial s_{\alpha }}{\partial \xi_{\alpha }}\right)\left(\frac{\partial s_{\beta }}{\partial \xi_{\beta }}\right)$$

The non-linear fitting requires an objective function (or error function) for each element in the mesh. The element objective function is the sum of the square of distances between each data point and its projection. In this procedure, the nodal parameters are interpolated to find the projected points. Therefore, the parameters that minimize the objective function are determined which indeed reduce the data error in the mesh approximation. Then, the resulting solution for elements are assembled to give the global mesh equations. The objective functions to be minimized in the fit consist of two components, the data error and a smoothing constraint. The data error is the weighted sum of the square of the distances between each data point and its projection. The smoothing constraint is incorporated in the objective function as a penalty function using a non-linear Sobolev smoothing. The Sobolev function regularizes the deformation of the surface because can sometimes be noisy (Bradley et al., 1997; Terzopoulos, 1986). The complete data objective function, *F*, of a face with respect to the global shape parameters is given by

(Equation 11) $$F\left(u_{n}\right)=\overset{N}{\underset{d=1}{\sum }}\gamma_{d}{\left\|u\left(\xi_{1d},\xi_{2d}\right)-z_{d}\right\|}^{2}+F_{s}\left(u_{n}\right)$$

where u is a vector of mesh parameters, $\gamma_{d}$ is a weight factor for each point, z is geometric position which can be given by local element coordinate *ξ*, $z_{d}$ is the spatial coordinates of the data point. $F_{s}\left(u_{n}\right)$, the Sobolev function, for two *ξ* directions is given by

(Equation 12) $$F_{s}\left(u_{n}\right)=\underset{\text{Ω}}{\int }\left\{\alpha \left({\left||\frac{\partial u}{\partial \xi_{1}}|\right|}^{2}+{\left||\frac{\partial u}{\partial \xi_{2}}|\right|}^{2}\right)+\beta \left({\left||\frac{\partial^{2}u}{\partial \xi_{1}^{2}}|\right|}^{2}+{\left||\frac{\partial^{2}u}{\partial \xi_{2}^{2}}|\right|}^{2}+2{\left||\frac{\partial^{2}u}{\partial \xi_{1}\partial \xi_{2}}|\right|}^{2}\right)\right\}d\xi$$

where *α* and *β* are the Sobolev weights that control the tension of the surface and the degree of surface curvature, respectively.

Next, to solve the fitting, face equations are obtained by differentiating the objective function (Equation 11) with respect to each DoF which are then assembled into a linear system of equations. This system, which includes a global equation set from all the individual element matrices, governs the entire mesh. Since, the scale factors (arc-length) and data projections are held constant during the process of differentiation, the resultant mesh may not represent the best fit to the data. Therefore, the obtained linear system of equations is solved iteratively to update the mesh parameters, and scale factors are recalculated in order to obtain new optimum. The iterative fitting procedure is continued until the desired root mean square (RMS) is obtained and/or the RMS error does not show significant changes between iterations. The RMS error is defined as

(Equation 13) $$RMS=\sqrt{\frac{\sum_{d-1}^{N}{\left\|u\left(\xi_{d}\right)-z_{d}\right\|}^{2}}{N}}$$

where *N* is the total number of data points and $u\left(\xi_{d}\right)-z_{d}$ is the distance between the data point and its projection. Figure S5 shows how a *linear* elemental mesh is deformed into a smooth shape during a typical fitting procedure.

#### Model geometry of the C-looping heart

The subjects used in this study are obtained from different embryos. Therefore, to capture the spatial and temporal dynamic of the C-looping from these datasets, the heart subjects are required to be spatially aligned into the same coordinate system and be time-ordered based on their developmental stages.

##### Spatial registration

In Amira, 3D data objects are embedded in a virtual 3D space. This space has a unique global coordinate system. The heart datasets are placed in different locations with respect to the global axes in this space (Figure 3C). To capture the spatial dynamic of geometrical changes between time points, four hearts are needed to be overlaid. To align four heart data objects into the global coordinate system, a rigid transformation with no scaling was performed. The cranial and caudal attachment points of the hearts to the dorsal wall were used as landmarks for registration. This resulted in a geometric transformation matrix that consisted of translation and rotation (with no scaling) for each heart. Next, the geometric transformation was applied to both endocardial and myocardial surface datasets using *Transformation editor* to change the spatial position of the hearts with respect to the global axes (Figure 3C). The point cloud datasets were generated from the aligned surface for volume fitting.

##### Temporal staging of hearts

The confocal and micro-CT images were used to temporally order the subjects based on their developmental stage. This was not a straightforward task; two approaches were taken to stage each heart into their relative orders: (i) It is shown that during ventral bending and rightward rotation the heart progressively detaches from the dorsal wall (Miquerol and Kelly, 2013). Micro-CT data was used to study the heart with respect to the embryonic body. To measure the level of detachment of the heart from the dorsal wall, the distance between the arterial and venous poles was measured in the DataViewer software. In addition, the distance between the detachment points of the heart from the dorsal wall was measured. The ratio of the detachment distance to the distance of the poles was used as a factor of the level of the detachment. (ii) The heart rotates rightward during looping (Männer, 2000; Shi et al., 2014b). The degree of rotation was used for the temporal staging. To compare the degree of rotation between subjects, aligned datasets were used. The endocardial layer was used as a reference to the mid-plane of the heart, and the degree of the rotation of the endocardium was qualitatively used to compare the rotation of the hearts.

##### 3D single cell scale segmentation using deep learning

The most crucial, laborious, and time consuming part of network training is dataset preparation and pre-processing. Next, it is important to design a proper, suitable neural network model. The 3D confocal image stacks of four chicken embryo hearts, which were used to generate the geometric model of the hearts, were used in this section to segment individual myocardial cells within each heart. The dataset consists of 4 volumetric image stacks with 170 to 250 image slices of 2328×2328 pixels that contain slices from the entire embryonic heart and surrounding regions. Each image stack has three channels, one for the nuclei and two for the cell membranes. A DAPI stain was used to stain the cellular nuclei. Cell boundaries were stained with two stains: (i) NCAM-1 stains myocardial cell membranes specifically, and (ii) WGA marks cell membranes globally in the tissue (Figures S6A and S6B).

##### Data pre-processing

Some adjustments to the raw data were made to improve the quality of the images. Proper pre-processing makes the image more meaningful for an algorithm. To improve the signal-to-noise ratio, yet faithfully preserve the boundaries information that defines the shape of the cells, image intensity and colour processing and background correction were employed in Fiji-imageJ (ImageJ 1.52i, https://imagej.nih.gov/ij/). First, to correct for over- and under-saturated pixels in the images, the *Look UpTable*(LUT) plugin in the Fiji-imageJ was used. Next, a *rolling ball* algorithm was performed on the image stacks to correct for an uneven illuminated background. The algorithm works by averaging over a very large ball around every pixel to determine a local background value. This value is then subtracted from the original image to remove large spatial variations of the background intensities. Deconvolution was used to improve image quality. However, it did not improve the Z axis resolution which was probably because of the high interval in the Z axis and therefore lack of information in this axis.

##### Training dataset

A relatively good representation from one time point image data was chosen to build the training set. This sample included cell and background information, and the quality of the signal was checked for both the XZ andYZ planes. Two different datasets were prepared in this section to evaluate the effect of data size and training performance. The first, smaller dataset consisted of a region of 160×160×50 pixels (equivalent to approximately 38×38×14 $\mu m^{3}$) whereas the second prepared dataset was selected from a region of 392×392×350 pixels (equivalent to approximately 95×95×85 $\mu m^{3}$) in size. Both these data were later *augmented* to generate larger datasets (see Section Data augmentation below). Each dataset was later split into a *training dataset* and a *validation dataset* with a ratio of 8−to−2, respectively (i.e., 80% of the data assigned for training and 20% was used for validation).

##### Ground truth labelling

Ground truth labelling refers to segmenting and annotating specific regions in an image. Here, two separate labelling approaches were taken: 1) three distinct labels for background (labelled 0), cell membrane (labelled 1), and cell internal space (labelled 2); 2) background and cell membrane combined as one class (labelled 0) and cell internal space (labelled 1). The segmentation and labelling were manual procedures performed in the *Segmentation Editor* of Amira software. The two training sets were fed into the same neural network model separately to evaluate the prediction results. The second labelling approach produced far better results; thus this labelling approach was used for further training and prediction. Note that there are large gaps between cells and cell internal spaces are smaller. This has several explanations. For example, the cell membrane signal is thicker than the actual membrane because the confocal optical system introduces blurring. The region growing algorithm which was initially used for manual segmentation of cell internal space did not detect the exact cell membrane pixels (the edge where the algorithm would stop). This was in fact because of the blurring effect and the result is slight under-segmentation leading to larger space between cells. Trying to overcome this issue caused extreme over-segmentation of cells which was not desired because it would have caused multiple cell segmentation to fuse and detected as one cell. This is of course a limitation in the training dataset; however, this was still the best approach available at the time.

##### Data striding and slicing

In image-based Convolutional Neural Network (CNN) training, an issue arises when image size is too large to be fed into the computer memory. To the best of the author’s knowledge, the current state of research uses image downsampling techniques to overcome the memory issue by reducing the size of the images. Image downsampling approaches, however, may lead to the loss of useful information in the image and, in turn, result in poor training and prediction. Therefore, an approach was designed and developed in this thesis that overcomes the memory management issue while preserving image quality and information. This approach works by *striding* and *slicing* each image tile into smaller images with the same size. Each tile size is 2328×2328 pixels in the *X* and *Y* axes, and between 850 and 1250 pixels in the *Z* axis. In order to reduce the image size, unwanted regions where no myocardial signal was present were deleted using Amira. Also, the input image was cropped based on window size and stride to make sure that all the images of the striding have the same size with no offset. Furthermore, to preserve the information that might be lost during convolutions in CNN, an area of ’overlap’ was integrated during slicing. The schematic in Figure S6C shows the process of striding and slicing a three-dimensional image.

After trial and error, the optimal image size for the CNN architecture in this study was 128×128×128 pixels. Therefore, in the algorithm used to slice the confocal heart images, the slicing size was set to (128,128,128) doubt whereas 78 pixels were selected for striding in all directions, i.e., (78,78,78). This meant that a window of size 128×128×128 starts from the top left point of image and moves 78 pixels along the *Z*axis with same values for *X* and *Y*, and generates the next image. This process repeats until the window reaches the last value for *Z*. Next, the *Z* value resets to 1 but the *X* value stays the same while the window moves along the *Y* axis with 78 pixels for each striding until it reaches the last value for *Y*. Lastly, the window moves along the *X* axis until the entire image is covered. The outcome is sub-images of size 128×128×128 pixels with overlapping regions of 50 pixels in all three directions (i.e., overlapping = window size – stride value). This algorithm ensures that the images are properly subdivided into smaller grids with traceable indexes.

##### Data augmentation

A common method to increase the amount of data for training is augmentation by inducing minor alterations to the existing data. These minor alterations include translation, flip, rotation, and warping. This process also helps the CNN model to be invariant to common image transformations. These additional synthetically created/modified data from the existing confocal images account for different orientations, scales, and locations in order to generalize the model. Here, the confocal training dataset underwent an augmentation process by randomly flipping the image slices. This process was performed *on the fly* during training.

##### Network architecture

A variant of a CNN architecture called **U-net** was used in this study to segment the myocardial cells. U-net was specifically developed for semantic segmentation of biomedical images (Ronneberger et al., 2015). U-net is a robust network that involves the whole context of the images in the computation and directly produces the segmentation masks. Figure S6D shows the architecture of a U-net model. The architecture consists of *encoder* and *decoder* components with *skip connections* to pass activation maps between the components. The design of the network helps to combine less processed input images with more processed feature maps.

The network consists of an encoder (or contracting) path and a decoder (or expansive) path. The encoder path follows a typical CNN on which there is a repeated application of two 3×3×3 unpadded convolutions, each followed by ReLU activation function and a 2×2×2 max pooling operation with stride of 2 for downsampling. The decoder path, on the other hand, consists of an upsampling of the feature map followed by a 2×2×2 convolution (’up-convolution’) that halves the number of feature channels. Furthermore, the decoder includes a concatenation of the corresponding feature map from the encoder path and two 3×3×3 convolutions each followed by ReLU. The last step involves a 1×1×1 convolution layer to map each feature vector to the desired number of foreground (myocardial cells) and background (everything else) classes.

##### Training

All training and experiments were performed using the Tensorflow (v.1.14, https://www.tensorflow.org/) and Keras (v2.1.6, https://keras.io/) frameworks. The model is trained with randomly sampled patches of size 128×128×128 voxels and batch size 2 and a total of 300 epochs. Here, each epoch was defined as an iteration over 100 batches. An *Adam* optimiser was used to train the model with an initial learning rate of $lr_{init}=1\cdot 10^{4}$. The subsequent learning rate schedules were: $lr_{init}\cdot 0.985^{epoch}$ and a 10 weight decay of $10^{-3}$.

Because confocal images suffer from an extensive class imbalance (i.e., approximately >95% background v.s. <5% signals for myocardial tissue from which cells are labelled as a class), a multi-class Dice loss function approach was taken. To understand this function, a Dice Coefficient (Sørensen-Dice Score) must first be introduced. A Dice Coefficient is a similarity metric. First, the positive elements from a neural network output that are equal to the ground truth are found. Next, this result is divided by the total number of positives in both:

(Equation 14) $$Dice=\frac{2|A\cap B|}{\left|A|+|B\right|}=\frac{2\left(Positives\right)}{Positives+GroundTruthPositives}$$

Dice function is similar to Dice Coefficient where output from the softmax function is used to compute the dice coefficients so that the dice function becomes differentiable:

(Equation 15) $$DiceLoss=-\frac{2}{C}\overset{C}{\underset{i}{\sum }}\frac{\sum_{k}S_{i}^{k}L_{i}^{k}}{\sum_{k}S_{i}^{k}+\sum_{k}L_{i}^{k}}$$

where *C* is the number of classes and *k* is every pixel in the image. This function is currently one of the most used in most image segmentation studies. This formulation is differentiable and is known to be readily integrated into the network.

##### Myocardial cell prediction and evaluation

The main goal of this section was to be able to predict myocardial cells in the confocal images and segment them from the rest of the image. In order to evaluate the segmentation results of the model, a *k*-fold cross-validation was performed with k=5. The resulting five networks obtained by the corresponding cross-validation were used as an ensemble to predict the validation dataset created in Section Training dataset.

##### Data post-processing: masking

As previously discussed in Section Segmentation workflow in Amira, the myocardial layer was segmented semi-automatically in Amira. Here, the segmented binary image of the myocardial layer was used as a mask to isolate cells in the myocardium from other tissues in the image after the prediction step of the model.

##### Data post-processing: Slice merging

It was previously mentioned that to reduce image size into memory for training, each confocal image tile was sliced into smaller images using a striding and slicing method developed in this chapter. After the cell prediction step, these small images must be ‘merged’ back together in order to create the original confocal tile. A merging algorithm, which is almost the *reverse* slicing, was developed to generate the original 3D images, as shown in Figure S6E.

##### Data post-processing: Reconstructing the entire heart

Once all of the confocal image tiles of a given heart were merged and obtained, a stitching method was used in Amira to reconstruct the entire heart. The 3D binary image stacks of the datasets were downsized in size by a half in Fiji-ImageJ (ImageJ 1.52i, https://imagej.nih.gov/ij/) because the entire heart image was too large to be handled computationally. Next, the down-sized images were imported into Amira where more pre-processing was performed for further analysis.

#### Cell-level analysis

##### Spatial alignment in amira

In Amira, a data object in a 3D space is defined by both a global coordinate system and a local coordinate system. The local coordinate system is the point coordinates of an object in the bounding box of a 3D volume. At first, the four datasets are located at different locations in space. For each sample the global and local axes are aligned (Figure S7A), but the four hearts are not aligned with respect to each other. To be able to capture the dynamics of C-looping, geometric registration was performed (see Section Spatial registration). To compare the cell level information with the tissue geometry, the same transformation used for the heart geometry needs to be used. In Amira, a transformation can be copied from one dataset and applied to another. Therefore, the rigid geometric transformation of the myocardial surface of each heart was applied to its corresponding cell segmentation dataset. This transformation, however, is not applied to the local coordinates because the local coordinates remain unchanged by a geometric transformation. As a result, the four hearts are not aligned with respect to their local coordinates (Figure S7B). Because the volume datasets are going to be used for cell feature extraction (including orientation parameters), the initial local coordinates need to be transformed into the global coordinates. To align the local coordinates of all samples with the global coordinate system, the *Resample Transformed Image* module was used which reorients cell level volume data along the given reference by sampling the dataset on a lattice parallel to the reference. Figure S7C shows the final result in which global and local axes for all samples are aligned. Note that the axes alignments, which are not necessarily overlapping, are required for orientation analysis. Using these aligned datasets, the cell features could be compared within and between time points and with respect to the tissue level geometry.

##### Cell feature extraction in amira

Aligned cell datasets were used to quantify different cellular parameters. First, the *Separate Objects* module was used to separate connected cells if there was any connection between two separate cells. This module utilizes a high-level combination of watershed, distance transform, and numerical reconstruction algorithms to compute watershed lines on a distance map of a binary image. Trials were made on a cropped dataset to tune settings in order to make sure the cells were not over-separated. Then, the *Labelling* module was applied to index all of the individual cells within the entire heart. The *Labelling* module performs a connectivity analysis of individual objects in the entire 3D binary stack to index all of the disconnected objects. After that, the *Label Analysis* module was applied. This module computes a set of measures on each cell (connected component) of the input image stack. There is a comprehensive list of measures in the port *measure* of the *Label Analysis* module. Various options are available for 3D analysis of an object’s size and shape. In Amira the principal *Moments of Inertia* is used to compute global characteristics of an object’s shape in which the centroid (centre of mass), and the major and minor inertia axes of an object are defined. The covariance matrix is then extracted from this analysis and used to define shape and orientation measures. The desired measures can be selected to be applied to objects. The label analysis generates a spreadsheet with the result values for each individual cell along with its coordinate information.

The measures selected in *Label Analysis* for this study are listed in Table S1 with their Amira definitions. Volume3d was used for cell size analysis, anisotropy was used to measure a cell’s deviation from a spherical shape, and eigenvectors and eigenvalues were used to analyse the orientation and the stretch magnitude of the cell ellipsoids.

Figure S8 shows Time Point 2 in which the individual cells within the entire heart are indexed and coloured in Amira (Figure S8C). Figure S8D shows a snapshot of a *Label Analysis* result in Amira which was exported as a CSV file. All the indexed cells are listed in the CSV file with their coordinates and their measured values. To the author’s knowledge it is the first complete 3D dataset in the field of individual cells with measured cellular features of an entire heart.

##### Mapping cell data onto a geometric finite element model

An exported CSV file from a label analysis in Amira includes the raw data of the location of the individual cells in the global coordinate system (*X*, *Y*, *Z*) and their corresponding values for the measurements. An OpenCMISS data file was created for each parameter from a CSV file using a Python script in whichdata points (individual cells) are defined with their field (given measure value).

These data points were then projected onto the surface of the corresponding FE mesh to quantify which region of the heart (in fact, what element of the mesh) the cells (data points) were located in. This projection then allows for a spatial analysis within each heart and a temporal analysis of the same region (corresponding mesh elements) between stages. The data projection was carried out using the method described for geometric fitting in Section Volume fitting of the heart geometry, with slightly different settings. The data points were fitted to a volume mesh in the geometry fitting, whereas here, the data points were projected onto the outer surface of the geometrically fitted meshes. This was done as observing the spatial pattern through the thickness of the myocardial wall was neither helpful nor informative for spatial analysis, especially as the myocardium is composed of only two layers of cells.

##### Analysis of mapped cell data

The data projection resulted in an element-wise corresponding dataset. To perform a spatial analysis quantitatively within, and between, time points, each element of the original FE meshes (i.e., coarse meshes) was divided into 5×5 bins in order to improve spatial data resolution. This resulted in a bin-wise corresponding dataset. It should be noted that the size of the elements, and in turn, the bins are different within and between hearts. However, the density of cells would correct for that and would therefore normalize the changes. Next, a large data frame was constructed in which raw values for the cellular parameters of the projected cells were averaged in each bin of the geometric meshes. Various data frames were then constructed from the main data frame, including time-wise and region-wise data frames. The time-wise data frames were constructed according to the developmental stages, i.e., Time Points 1, 2, 3, and 4. For region-wise data frames, three regions were defined as precisely as possible using element bins, image data, and the visualized patterns of the four hearts to define the outflow tract (OFT), ventral (outer curvature), and dorsal (inner curvature) regions (Figure 6A). However, defining the exact border of these regions was difficult because there are no anatomical boundaries between regions. OpenCMISS-Zinc (http://opencmiss.org/about.html) was used for visualization purposes. Here, each cell had two *fields*: 1) the global Cartesian coordinates, and 2) the raw value for a given cell feature (a scalar or a vector).

##### Analysis of mapping field

In order to parametrize the spatial and temporal variation of cellular features, field fitting was used. Each individual cell feature data was fitted using bicubic Hermite interpolation functions to produce the fitted field for the feature. This resulted in a smoothly continuous variation in feature values. Here, a refined version of the geometric meshes was used in which each element was equally divided into 2 ×2 sub-elements. A refined mesh was used instead of a coarse mesh to increase the data variation that could be captured by the field. For these field fittings, the smoothing parameters were adjusted to ensure a smooth fit.

The cell feature field resulting from field fitting was imported into OpenCMISS-Zinc to qualitatively visualize and explore the spatial and temporal patterns of various cell features. Cell features were described as a *field* on the surface geometry.

##### Analysis of cell variance

In addition to the parameter measured in Amira, to study the local changes in cell parameters with respect to neighbouring values, a variance analysis was carried out. To calculate the variance value for individual cells within the heart, the nearest neighbour and variance algorithms from Scikit-learn and numpy libraries in Python were used to compute the variance between each cell with nine nearest cells for the raw values of a given parameter. Variance was also treated as a field (as with measured cellular features) and used with field fitting to explore the spatiotemporal pattern.

#### Tissue-level analysis of growth

##### Deformation

The FE meshes of two subsequent time points were used as the reference (undeformed) and current (deformed) geometries to calculate the deformation gradient tensor. From a continuum mechanics standpoint, the *deformation gradient tensor*, F, is one of the most fundamental measures of deformation (Gurtin, 1982). This measure is a second order tensor that maps line elements in the reference state onto line elements in the deformed state. Consider a line element dX within the reference state moving from position X to become the line elements dx at position x within the deformed state:

(Equation 16) dx=X(X+dX)−X(X)=GradXdX

where Grad denotes the material deformation gradient ∂X/∂X (Grad to represents a gradient with respect to the material coordinates whereas grad represents a gradient with respect to the spatial coordinates). The mapping function, X, is function of the variable Xwhich can be conveniently written as x=x(X,t) to denote x as the value of X at X so that:

(Equation 17) $$F=\frac{\partial x}{\partial X}=\text{Grad}\;x$$

with

(Equation 18) dx=FdX

Thus, the deformation gradient tensor, F describes the deformation in the neighbourhood of a point, mapping the line element dX moving from X in the reference state to the line element dx emanating from x in the deformed state.

Recall that a coarse mesh was divided into 5×5 bins to increase the spatial resolution for cellular analysis. The cellular values were then averaged within each bin. To be consistent with the cellular level analysis, 5×5 subregions were defined along $\xi_{1}$ and $\xi_{2}$ directions for each element at regular intervals. No subregion along $\xi_{3}$ was defined. The deformation gradient tensor, F, was calculated at the centre of the subregions using (Equation 17), where dX was the material particles in the reference, undeformed subregion (e.g., Time Point 1) and dx was the spatial particles in the corresponding region of the deformed geometry (e.g., Time Point 2).

##### Volume changes

To obtain the volume change in the corresponding subregions of elements from the reference myocardium to the deformed tissue configuration, the Jacobian of the deformation gradient was computed. Because the cellular analysis used ΔV (described later in next section), *J* and dV can be used to compute ΔV:

(Equation 19) ΔV=dv−dV=JdV−dV=dV(J−1)

where J=detF.

##### Stretch and orientation changes

A singular value decomposition (SVD) of the deformation gradient tensor, F, was performed to extract information about the three principal stretch ratios $\lambda_{1},\lambda_{2},$ and $\lambda_{3}$ as well as the tissue orientation:

(Equation 20) $$\hat{F}=U\text{Σ}V^{T}$$

where $\hat{F}$ is the polar SVD of the deformation gradient, F. Here, this equation is defined such that the rotation tensor R is given by UV and the stretch tensor is given by Σ.

#### Cell-level analysis of growth

Cell features for each individual bin were measured and the bin mean value for each cellular feature was stored. For each bin for each time point the extracted cell features as well as the computed tissue deformation gradient, volume change, and orientation were stored.

##### Cell number, size, and shape changes

To measure changes of the myocardial cell features between two subsequent time points (e.g., the reference and deformed configurations), the difference between the values for the corresponding bins at each time point were calculated. This resulted in three separate deformation datasets: (i) from Time Point 1 to Time Point 2 ($\text{Δ}T_{1}$), (ii) Time Point 2 to Time Point 3 ($\text{Δ}T_{2}$), and (iii) Time Point 3 to Time Point 4 ($\text{Δ}T_{3}$) for measurements of changes in the number of cells ($\text{Δ}N_{cell}$), cell volume ($\text{Δ}V_{cell}$), ICS volume ($\text{Δ}V_{ICS}$), cell density ($\text{Δ}\rho_{cell}$), and cell anisotropy ($\text{Δ}A_{cell}$).

##### Cell orientation changes

To study the changes of cell orientation between two time points, principal eigenvectors ($v_{t}^{i},i=1,\ldots ,3$ and t=1,…,4) for two corresponding bins of two time points were used to compute the three principal rotation matrices, $R_{\text{Δ}T_{j}}^{i}$ (j=1,…,3), between the two bins. First the axis unit vectors, a, of the two corresponding eigenvectors were computed:

(Equation 21) $$a_{T_{j}}^{i}=\frac{v_{t}^{i}\times v_{t+1}^{i}}{\left\|v_{t}^{i}\times v_{t+1}^{i}\right\|}$$

where a is perpendicular to both vectors, $v_{t}^{i}$ and $v_{t+1}^{i}$. Next the angles between these vectors were obtained from:

(Equation 22) $$\begin{array}{l} \sin\;\theta_{\text{Δ}T_{j}}^{i}=\frac{v_{t}^{i}\times v_{t+1}^{i}}{\left\|v_{t}^{i}\right\|\;\left\|v_{t+1}^{i}\right\|}\\ \cos\;\theta_{\text{Δ}T_{j}}^{i}=\frac{v_{t}^{i}\cdot v_{t+1}^{i}}{\left\|v_{t}^{i}\right\|\;\left\|v_{t+1}^{i}\right\|} \end{array}$$

The rotation matrix was then constructed using the two above equations:

(Equation 23) $$R_{\text{Δ}T_{j}}^{i}=\left(\text{cos}\theta_{\text{Δ}T_{j}}^{i}\right)\;I+\left(\text{sin}\theta_{\text{Δ}T_{j}}^{i}\right)\;a_{\text{Δ}T_{j}}^{i}+\left(1-\text{cos}\theta_{\text{Δ}T_{j}}^{i}\right)\;\left(a_{\text{Δ}T_{j}}^{i}⊗a_{\text{Δ}T_{j}}^{i}\right)$$

where⊗ is the outer product.

#### Integrative cell-tissue growth analysis

The two datasets generated at the tissue level and cell level were used hereafter to study the correlation between tissue level growth and cell level features.

##### Analysis of cell features and tissue volume

To explore what cellular features affect the volume changes at tissue level ($\text{Δ}V_{tissue}$) a General Linear Model (GLM) was fitted in SPSS. The explanatory variables measured were changes in the number of cells ($\text{Δ}N_{cell}$), cell volume ($\text{Δ}V_{cell}$), ICS Volume ($\text{Δ}V_{ICS}$), cell density ($\text{Δ}\rho_{cell}$), and cell anisotropy ($\text{Δ}A_{cell}$).

Also, to study whether the relationship between cellular features and tissue growth differs spatially and/or temporally, Region and Time variables were used as fixed factors in the model fitting. For the regional analysis, different regional segmentations were defined within the hearts and examined for the best model fit. The different region segmentations examined were a three region segmentation (the OFT, dorsal and ventral regions), a four region segmentation (the OFT, dorsal, ventral, and lateral regions between the dorsal and ventral regions), and a five region segmentation (the OFT, dorsal, ventral, lateral region and the caudal part of the heart). The four region segmentation resulted in the best mode fit and is used for region analysis in the model.

A data exploration and distribution analysis was performed for all variables in SPSS. The response and explanatory variables revealed different distribution patterns and no variable showed normal (Gaussian) distribution (see Table S2). Therefore, all variables were transformed to normal distributions in order to satisfy the GLM assumptions and to improve the predictive ability of the model by making a better fit to the data; hence making the relationship between variables more meaningful and clear (Abbott, 2014). For this purpose, Templeton’s two-step transformation was used (Templeton, 2011). This method relies on two steps: firstly, data was converted to a uniformly distributed probability distribution using percentile ranking and, secondly, the inverse-normal transformation was applied resulting in a normally distributed data (Templeton, 2011). The result was satisfactory and all transformed variables revealed a normal distribution and passed normality tests. This transformation also corrected for the outliers.

The pairwise correlations analysis between all explanatory variables showed that there was a linear correlation between the change in the number of cells and the change in the cell density (which was to be expected as the cell density depends on the number of cells). Therefore, only one of the change in the number of cells and the change in the cell density was included in the model to avoid a multicollinearity issue.

Because there were multiple explanatory variables, and all variables including the response variable were successfully transformed to a normal distribution, a multiple linear regression model was fitted to the data. To understand which variables should be included in the model, a step-up strategy was used for building the model. The model started with no explanatory variables which resulted in an intercept only model in which the intercept coefficient was the mean of the response variable. The explanatory variables were then added one at a time to see whether, and by how much, the added variable affected the model. Model goodness of fit was performed using both numerical and graphical methods. For the numerical analysis, the *Akaike’s Information Criteria* (AIC) was used to cross check the alternative models through the step-up process. The variable added to the model was kept in the model if the AIC decreased and was removed from the model if the AIC was increased (Bozdogan, 1987). The *R Squared* value and the significance level of the variable was also taken into account. For the graphical analysis, residual plots were used. Graphical methods are commonly used for model goodness of fit checks because they readily illustrate a broad range of the complex aspects of the relationship between the model and the data.

##### Analysis of cell and tissue orientation

To investigate any relationships in changes in the orientation between the cellular level and the tissue level, the orientation values for each corresponding bin at a given ΔT were analysed. The cosine of the angle between each column of the tissue rotation matrix $R_{tissue}$ and the cell rotation matrix $R_{cell}$ was computed by calculating the inner product of the vectors:

(Equation 24) $$\text{cos}\theta_{\text{Δ}T_{j}}^{ki}=c_{\text{Δ}T_{j_{tissue}}}^{ki}\cdot \;c_{\text{Δ}T_{j_{cell}}}^{ki}$$

where $\text{cos}\theta_{\text{Δ}T_{j}}^{ki}$ is the cosine of the angle, *θ*, between the $k^{th}$ column vector c of the $i^{th}$ principal rotation matrix of the corresponding tissue and cell bins for $\text{Δ}T_{j}$. This analysis, therefore, resulted in values ranging from cosθ=−1 to 1 where 0 indicates that the orientation change of the corresponding tissue and cell bins are perpendicular (i.e., $\theta =\pi /2=90^{\circ }$) and −1 and 1 indicates that they are exactly aligned. Because the direction of the vectors is not of interest, the absolute value of cosθ was used for this analysis.

#### Software tools

A variety of software tools were utilized in different parts of this study. The majority of the data preparation, analysis, and plotting was performed using the Python programming environment (versions 2.7 and 3.6) and a number of different library packages. For data frame construction, *Pandas* and built-in CSV libraries in Python were used. Data visualization and plotting were performed using Python scripting with the *Matplotlib*, and *Seaborn* libraries. Geometric and field fitting was performed using our in-house software package OpenCMISS (Bradley et al., 2011), and the graphical rendering and visualisation of the heart volume geometries and their associated deformation and orientation fields were done using another in-house open-source software OpenCMISS-Zinc. All standard statistical techniques, including data transformation, normalization, Generzlised Linear Modeling, the Mann-Whitney U test, and Bonferroni correction were performed in SPSS (IBM Corp. Released 2017. IBM SPSS Statistics for Windows, Version 25.0.Armonk, NY: IBM Corp) and Python using *Numpy*, *Scipy*, *Statsmodels*, and *Scikit-learn* libraries. Standard image processing techniques were employed using Amira™ Software (version 6.5.0, Thermo Fisher Scientific) as well as the open-source software ImageJ-Fiji (version 1.52i). The acquired 3D micro-CT datasets were reconstructed and processed using InstaRecon CBR, SkyScan DataViewer, and SkyScan CTVox softwares. For confocal imaging, a Zeiss LSM 710 inverted confocal microscope and its software, ZEN, were used.

### Quantification and statistical analysis

A comprehensive data exploration and distribution tests for all variables were carried out in SPSS before and after data transformation. Statistical analysis were required to quantitatively analyse the temporal and spatial pattern. Because our datasets from the cell feature analysis did not pass a normality test, the Mann-Whitney U test was used. The Mann-Whitney U test is a popular non-parametric test of the null hypothesis that two samples come from the same population. This test is appropriate when comparing two samples with non-normal distributions. Indeed, the Mann-Whitney U test can be used in place of a t-test when the outcome is not normally distributed. A Bonferroni correction was used to correct for the problem of multiple comparisons where needed, for example when three regions were compared pairwise within each time point. For Integrative cell-tissue growth analysis, a GLM statistical modelling was performed in SPSS. Full details on each statistical test are presented in appropriate sections in the paper.

## Data Availability

The authors declare that all the data supporting the findings of this study are available within the article and its supplemental information files or from the corresponding author upon reasonable request. All data presented in this study, including raw, processed and segmented image stacks, have been deposited in a reserved Mendeley Data server at the following addresses:• Mendeley Data: https://doi.org/10.17632/jwj6m5yxct.1 • Mendeley Data: https://doi.org/10.17632/hww6c4yvhp.1 • Mendeley Data: https://doi.org/10.17632/fckszv3tp3.1 • Mendeley Data: https://doi.org/10.17632/wc3yc9rh7h.1 and are publicly available as of the date of publication. DOIs are listed in the key resources table. Geometric and field fitting of the hearts with the 3D mapping data was performed with the OpenCMISS platform developed at the ABI and is available at: http://opencmiss.org/. Development of the 3D cell segmentation was achieved using the Python programming environment. The original code has been deposited at Github and is publicly available as of the date of publication. DOIs are listed in the key resources table. Any additional information required to re-analyse the data reported in this paper is available from the lead contact upon request.

## Footnotes

Supplemental information can be found online at https://doi.org/10.1016/j.isci.2022.104600.
