# Supplementary material for: A method for investigating spatiotemporal growth patterns at cell and tissue levels during C-looping in the embryonic chick heart
Source: iScience. 2022 Jun 14;25(7):104600. doi: 10.1016/j.isci.2022.104600 (PMC9253367; doi:10.1016/j.isci.2022.104600)
Supplement: Document S1. Figures S1–S16 and Table S1–S19 [file mmc1.pdf]

iScience, Volume *25*

Supplemental information

A method for investigating spatiotemporal growth patterns at cell and tissue levels during C-looping in the embryonic chick heart

Nazanin Ebrahimi, Mahyar Osanlouy, Chris P. Bradley, M. Fabiana Kubke, Dane A. Gerneke, and Peter J. Hunter

Supplementary Note 1. Related to Figures 6 and 7.

This section will attempt to carefully and extensively present the results from the spatio-temporal analysis of myocardial cell features during C-looping.

Entire heart analysis

To study heart growth during looping, growth related features were first compared between hearts at different time points to look at the overall changes.

Temporal patterns from fitted field

Results from field fitting were used to examine the temporal changes of the mean cell volume (size) and cell anisotropy (shape) fields over four time points. Inter-subject comparison was done by manually arranging the spectrum range between the minimum and maximum of values over the four samples (Supplementary Figure 9). This means that the local patterns within each time point can be hard to see. Therefore, intra-subject patterns are presented in a separate section below (see Region spatial patterns from field fitting).

Cell volume Supplementary Figure 9 (top row) shows the fitted value of mean cell volume in each bin over the mesh geometry. A white to red spectrum represents the smallest to largest cells in size. Time Point 2 showed an increase in the mean volume of cells in comparison with the three other time points. Time Point 3 shows the lightest colour and thus the smallest cells during the looping process.

Cell anisotropy Cell anisotropy shows a cell's deviation from a spherical shape. A white to red spectrum is used to show cells from a spherical shape (white) to a more elongated shape (red) (Supplementary Figure 9, bottom row). Time Point 2 shows a lighter shade of red in comparison with the three other time points which is representative of more spherical cells. Time Point 1 shows light red pattern locally. This will be discussed further in the section on spatial analysis (see Region spatial patterns from field fitting). No major difference is observable between Time Points 3 and 4.

Temporal patterns from projected cell data

Supplementary Figure 10 shows a quantification of cellular values both within each time point in the top row (Per heart), and their values in each bin of the geometric mesh in the bottom (Per bin). For bin-wise plotting, the projected data points were used to calculate the parameters within each bin. A Mann-Whitney U test was carried out for statistical significance. The significance levels are presented in the figure. Descriptive and test statistics are presented in Supplementary Tables 3 and 4.

Tissue volume The total tissue volume was measured in Amira using the label analysis module to measure the total volume of the segmented myocardial layer (Per heart). To measure the volume of tissue in each bin, projection into the geometric finite elements of the meshes were used. There was a total increase in the volume of the myocardial layer over time during C-looping. The bin-wise analysis shows the same pattern with significant changes, however, the increase between Time Point 2 and 3 showed less change (p-value=.020) (Supplementary Figure 10, "Tissue volume" column).

Cell number The total cell number is the sum of the number of cells projected within each heart and each bin for the "Per heart" and "Per bin" analysis, respectively. In total, the number of cells increased over time. However, the bin-wise analysis shows that there was no significant change in the number of cells from Time Points 3 to 4 (p-value=.088) (Supplementary Figure 10, "Cell number" column).

Cell volume The total cell volume is the summation of the volume of cells in the entire myocardial tissue (Per heart). Per bin plots show the average cell volume in the individual bins within each heart. "Per heart" and "Per bin" plots showed the same trend for cell volume changes. There was a dramatic increase in the volume of the cells at Time Point 2 (both the total volume occupied by cells in "Per heart" and the average volume of cells in bins in "Per bin"). It was followed by a drop in volume at Time Point 3. Total and average cell volume increased at Time Point 4 but did not reach the values of Time Point 2. All the changes showed statistically significant differences (Supplementary Figure 10, "Cell volume" column). The bin-wise pattern is in agreement with the results from field fitting cell volume (see Temporal patterns from fitted field) the top row in Supplementary Figure 9.

Inter-cellular space volume The Inter-Cellular Space (ICS) volume of each bin is extracted by subtracting the total volume occupied by the cells from the bin volume. The ICS volume showed an increasing trend over time. Changes were statistically significant for all time points (Supplementary Figure 10, "ICS volume" column).

Cell density Cell density was calculated from the total number of cells divided by the tissue volume. Cell density increased from Time Point 1 to 3. Time Point 4 showed the lowest cell density of both the heart-wise and bin-wise plots. All the changes in the bin-wise analysis were statistically significant (Supplementary Figure 10, "Density" column).

Region-based analysis

In addition to temporal change, spatial changes are also important during C-looping. For example, the differential growth between the ventral (outer curvature) and dorsal (inner curvature) regions in terms of cell proliferation and hypertrophy has been reported. For regional analysis, three defined regions (the OFT, ventral, and dorsal regions) were compared using two approaches, field fitting and cellular data analysis.

Regional spatial patterns from field fitting

A field fitting approach was used to examine the spatial pattern of different features within each time point (Figure 6b-e). Three parameters were explored for each time point, namely cell volume (for size), cell anisotropy (for shape), and cell orientation (for directionality). A white to red spectrum from the minimum and maximum values was used to visualise the spatial pattern. For the cell volume, white to red presents smaller to larger cells in size. For the cell anisotropy analysis, white to red shows a more spherical cell towards a more elongated cell. For cell orientation, sphere glyphs are used instead of a coloured field where the orientation of glyphs show the orientation of the principal eigenvector and the white to red spectrum represents the magnitude of the principal eigenvalue from the minimum to the maximum value.

Time Point 1 Figure 6b shows the straight heart tube at Time Point 1. The patterns from the different parameters were similar but did not completely overlap. The cranial part of the ventral region had larger cells in comparison with the caudal part of the ventral region and also with the OFT region. Lateral regions showed a patchy pattern. Cells in the middle to the cranial part of the ventral region were more elongated in comparison with the caudal part of the ventral region and with the OFT region. The OFT region, in general, contained smaller, more spherical cells. Right and left lateral regions exhibited a patchy pattern, however, more red shades (more elongated) than white (more spherical) could be seen. In terms of orientation, cells were mostly unaligned at this stage when the fitted field was compared to cellular data (Supplementary Figure 11). The principal eigenvalue showed a larger value in the middle part of the ventral region and a lower value in the OFT region which was consistent with the anisotropy result.

Time Point 2 Figure 6c shows Time Point 2 in which the heart showed some ventral bending. Fitting a cell volume field resulted in regions with larger cells along the dorsal wall in the OFT region and the cranial part of the outer curvature. There were also two sites in the middle part of the heart, along the inner curvature and lateral regions, with a darker shade of red representing larger cells. The rest of the heart showed a patchy pattern for the volume of cells. In terms of the shape of cells, the middle part of the heart including outer and inner curvature regions, showed more elongation in comparison with the OFT region and the caudal part of the heart. Cell orientation showed that cells were oriented circumferentially in the outer and inner curvature regions and more longitudinally in lateral regions. The magnitude of the principal eigenvalue of the cell ellipsoids (the colour spectrum for orientation) showed a pattern consistent with the cell anisotropy pattern. There were higher values for the outer and inner curvature regions and lower values for the OFT and lateral regions, especially the caudal part of the lateral regions. For the cell data (Supplementary Figure 12), the circumferential orientation in the outer and inner curvature regions can be seen, however, other parts showed a mixture of a more spherical shape with a random pattern for orientation which was consistent with the anisotropy and eigenvalue magnitude pattern.

Time Point 3 Time Point 3, in which the heart showed ventral bending and a more rightward rotation than Time Point 2, is shown in Figure 6d. The fitting result for cell volume showed smaller cells along the dorsal line and caudal part of the heart in comparison with other regions. The anisotropy pattern showed a few regions with more elongated cells in the outer and inner curvature regions. More spherical cells were located in the lateral regions, however, all regions looked patchy. The orientation pattern was similar to Time Point 2 with circumferentially oriented cells in the outer and inner curvature regions. In the anterior view, cells were oriented longitudinally in the lateral region. Comparison with the cell data (Supplementary Figure 13) confirmed the orientation pattern in the outer and inner curvature regions. The pattern for the principal eigenvalue of the cell ellipsoids represented by a white to red spectrum showed similarity with the cell anisotropy pattern.

Time Point 4 Figure 6e shows the C-looped heart at Time Point 4. Cell volume showed a patchy pattern. There were, however, some regions with larger cells in the outer curvature region. The cell anisotropy pattern showed regions with more elongated cells in the outer and inner curvature regions and, also, along the ventral line in the OFT region. Cells were oriented circumferentially in the outer and inner curvature regions, and longitudinally in the lateral regions. Comparison with cellular data (Supplementary Figure 14) confirmed the circumferential pattern along the ventral line of the outer curvature region. The magnitude of elongations showed higher values in the outer curvature, inner curvature, and a region at the ventral line of the OFT region. This was similar to the pattern for anisotropy.

Regional spatial patterns from projected cell data

To quantitatively examine whether the ventral (outer curvature), dorsal (inner curvature), and OFT regions showed any different features (growth), bins in these three regions were grouped. Different parameters were plotted and a Mann-Whitney U test was carried out for statistical significance. Since multiple two-way tests between the three regions were performed, a Bonferroni correction was applied to correct for these multiple comparisons. The significance levels are presented in Figure 6f-i. Descriptive statistics and test statistics are presented in Supplementary Tables 5 - 8, and 9 - 12, respectively.

Time Point 1 (See Figure 6f and Supplementary Table 9) Tissue volume, cell number, and ICS volume all showed a similar pattern at this time point. Ventral and dorsal regions had greater values for these parameters than the OFT region. However, there was no significant difference between the dorsal and ventral regions for these parameters (p-values of 1.00, .062, and .387 for the tissue volume, cell number, and ICS volume, respectively). The ventral region had significantly larger cells than both the OFT and dorsal regions (p-value<.001 for both comparisons). The dorsal region contained the smallest cells. The cell density was significantly different between the OFT region and ventral region (p-value=.023) with the ventral region having a slightly larger cell density.

In terms of the shape of cells, the dorsal and ventral regions had more elongated cells than the OFT. The dorsal region showed more elongation but the result was only marginally significant (p-value=.047). The OFT showed the most variation in cell shape.

Time Point 2 (See Figure 6g and Supplementary Table 10) Tissue volume was significantly different between all three regions (p-value<.001). The ventral region had the largest volume and dorsal region had the smallest volume of tissue. The ventral region had a significantly greater number of cells and a greater ICS volume than the OFT region and dorsal region (p-value<.001 for all comparisons). The OFT region had a larger ICS volume than the dorsal region (p-value<.001) but there was no difference in the number of cells between the OFT and dorsal region (p-value=.666). Cell volume did not have any significant differences between regions at this time point (p-values of .921 and .239 for comparisons between the OFT region vs the ventral region and the OFT region vs the dorsal region). The ventral region had slightly larger cells than the dorsal region (p-value=.039). The dorsal region showed the greatest density. No significant difference in density was found between the OFT region and ventral region (p-value=.112). Cells showed the most elongation in the dorsal region and the most sphericity in the OFT region (p-value<.001 for all comparisons).

Time Point 3 (See Figure 6h and Supplementary Table 11) The ventral region had the largest tissue volume by a significant amount (p-value<.001 for all comparisons). The larger tissue volume in the ventral region was accompanied by a significantly larger number of cells and a higher ICS volume than the OFT region and dorsal region (p-value<.001 for all comparisons). The OFT region had a smaller tissue volume and ICS volume than the dorsal region (p-value<.001 for the two comparisons), but there was no difference in the number of cells between these two regions (p-value=1.00). The OFT region and the ventral region contained larger cells than the dorsal region (p-value<.001 for the two comparisons). There was no significant difference in the average cell volume between the OFT and ventral regions (p-value=1.00). Cell density did not show any significant difference between the three regions (p-values of 1.00 (OFT vs ventral), .372 (OFT vs dorsal), and .187 (dorsal vs ventral)).

Similarly to the first two time points, the dorsal region had more elongated cells than the ventral region (p-value<.001) and the OFT region (p-value=.018). There was no significant difference in cell anisotropy between the OFT region and the ventral region (p-value=.060).

Time Point 4 (See Figure 6i and Supplementary Table 12) This time point showed a similar pattern to Time Point 3 for the tissue volume, cell number and ICS volume. The ventral region had the greatest value for all these parameters (p-value<.001 for all comparisons). The OFT region had smaller tissue and ICS volumes than the dorsal region (p-value<.001 for the two comparisons), but there was no difference in the number of cells (p-value=.06) between these two regions. There was no significant difference in the cell volume between all three regions (p-values=1.00 for all comparisons). The dorsal region showed a significantly lower density of cells in comparison with both the OFT region and ventral region (p-value<.001 for the two comparisons). The OFT region and the ventral region showed no difference in cell density (p-value=1.000).

There were significant differences in anisotropy between the three regions (p-value<.001 for the two comparisons). The dorsal and ventral regions showed the largest and smallest values for anisotropy.

Regional temporal pattern from cell data

In this section, parameters were studied separately over the four time points. Temporal changes within the three regions of the OFT, ventral and dorsal regions were studied. For each parameter a box plot is presented in which the OFT, ventral and dorsal regions of all four time points are shown. The changes of a given parameter between two subsequent time points were tested using a Mann-Whitney U test. The significance levels are presented in Figure 7. Descriptive statistics and test statistics are provided in Supplementary Tables 5 - 8 and 13 - 18, respectively.

Tissue volume (See Figure 7a and Supplementary Table 13) Tissue volume was measured as the volume of the individual bins within defined regions at the four time points. The volume of bins in the OFT region increased from Time Point 1 to 2, followed by a decrease at Time Point 3. The tissue volume showed an increase from Time Point 3 to 4 (p-values<.001 for all changes). The ventral region showed a continuous increase from Time Point 1 to 4. All these changes in the ventral region were statistically significant (all p-values<.0001). In the dorsal region there was no significant difference in the volume of bins between Time Point 1 and 2 (p-value=.573). The tissue volume then increased from Time Point 2 to 3 and also from Time Point 3 to 4 (all p-values<.001).

Cell number (see Figure 7b and Supplementary Table 14) The number of cells embedded in the individual bins within the defined regions was plotted to study the temporal changes over the four time points. The number of cells in the OFT region increased significantly from Time Point 1 to 2 (p-value<.0001). There was a smaller increase from Time Point 2 to 3 (p-value=.008). There was no significant change from Time Point 3 to 4 (p-value=.089). There were significant increases in the ventral region in all time periods from Time Point 1 to 4 (all p-values<.0001). In the dorsal region, on the other hand, there were no significant changes in number of cells throughout the four time points (p-values of .065, .122, and 0.343 from Time Point 1 to 4, respectively).

Cell volume (see Figure 7c and Supplementary Table 15) The average volume of cells in the bins within the defined regions was used to study the regional changes of volume over the four time points. All three regions showed the same temporal pattern. The average volume of cells increased from Time Point 1 to 2 followed by a drop at Time Point 3. From Time Point 3 to 4, the cell volume increased again but did not reach the cell volume of Time Point 2. These changes in the three regions and between the four time points were all statistically significant (all p-values<.0001).

Inter-cellular space volume (See Figure 7d and Supplementary Table 16) The volume of the inter-cellular space (ICS) in the bins within the defined regions was plotted for the four time points. The ICS volume at the OFT region increased from Time Point 1 to 2 (p-value<.0001). It then decreased from Time Point 2 to 3 (p-value=.004) followed by an increase at Time Point 4 (p-value<.0001). In the ventral region, the ICS volume increased significantly over time (all p-values<.0001). The volume of the ICS, in the dorsal region, decreased from Time Point 1 to 2 followed by increases at Time Points 3 and 4. All changes in the dorsal region were statistically significant, however, the significance level was lower between Time Points 1 and 2.

Cell density (see Figure 7e and Supplementary Table 17) Density of cells in the individual bins within the defined regions was plotted at the four time points. The density in the OFT region did not change between Time Point 1 and 2 (p-value=.148). It then increased at Time Point 3 and decreased at Time Point 4 (both p-values<.0001). The ventral region showed a decrease in the density between Time Point 1 and 2 (p-value=.008). The density increased significantly during the next time period (p-value<.001) followed by a decrease from Time Point 3 to 4 (p-value<.001). In the dorsal region, the density increased from Time Point 1 to 2 (p-value=.022). The density did not change significantly between Time 2 and 3 (p-values=.793) at the dorsal region. It then dropped significantly from Time Point 3 to 4 (p-value<.001).

Cell anisotropy (see Figure 7f and Supplementary Table 18) The values of cell anisotropy in the bins of the defined regions at the four time points were plotted. Within the OFT region, cells were more elongated at Time Points 1 and 3 than at Time Point 2 (all p-values<.001). There was no significant difference in cell anisotropy between Time Point 3 and 4 (p-value=.488). Cells in the ventral region were more spherical at Time Point 1 and 3 in comparison with Time Point 2 (both p-values<.001). At Time Point 4 cells were more elongated that Time Point 3. The dorsal region showed the same trend as the OFT region. Time Points 1 and 3 had more elongated cells than Time Point 2 (both p-values<.001) and there was no significant difference between Time Point 3 and 4 (p-value=.563).

Regional spatio-temporal pattern from the cell data

The overall observed spatial and temporal patterns are summarised in Figure S15. In this figure, the significance level is colour coded. White is for the changes that were not statistically significant. Changes coloured with all shades of red were significantly different. Red colour shows highly significant changes with a p-value<.001. The other two shades indicate lower levels of significance. In the spatial part, the result of the pair-wise comparisons between three regions are presented for all cellular features. For the temporal column, changes of all cellular features between two subsequent time points are shown at different regions.

Overall spatial pattern with respect to the temporal changes

Time Point 1 to 2 The ventral region exhibited a greater value than the OFT region for all parameters, except for the cell volume and density at Time Point 2. At Time Point 2, there was no difference in cell volume and density, although the cell volume decreased and density increased from Time Point 1 to Time Point 2 in both the OFT and ventral regions. Interestingly, the pattern between the ventral and dorsal regions changed considerably. At Time Point 1, the ventral region only differed from

the dorsal region by a greater cell volume and a smaller cell anisotropy. At Time Point 2, the ventral region showed larger tissue, cell, and ICS volumes, and a greater number of cells than the dorsal region. However, the difference in cell volume between the ventral and dorsal regions at Time Point 2 was not as large as Time Point 1. These two regions showed a temporal decrease in cell volume. All these changes resulted in higher cell density in the dorsal region at Time Point 2. The cell shape pattern, in which the dorsal region showed more cell elongation, remained unchanged between these two regions. There was also a considerable change in all patterns between the dorsal region and the OFT region at this time period. At Time Point 1, the dorsal region had a larger tissue volume with a greater number of cells and a greater value for the ICS volume. The cell volume was larger in the OFT. There was a difference in cell density between these two regions. At Time Point 2, the OFT region had greater value for the tissue and ICS volumes. From the temporal changes, these relational changes were due to increasing trends for tissue and ICS volume in the OFT region and a constant pattern and a decreasing trend in the dorsal region for the tissue and ICS volumes, respectively. The number of cells and cell volume at Time Point 1 were larger in the OFT and the dorsal regions, respectively. At Time Point 2 these regions showed no difference in these features. All these changes resulted in different patterns for cell density at the two time points where there was no difference in cell density at Time Point 1 and a greater value for the dorsal region at Time Point 2.

Time Point 2 to 3 The pattern between the OFT region and the ventral region remained almost unchanged between these two time points, where the ventral region had larger tissue and ICS volumes, and a larger number of cells. Also, there was no difference in cell volume and density between the two regions. In terms of cell shape, however, the pattern changed. At Time Point 2, the ventral region had more elongated cells, whereas at Time Point 3 there was no difference in anisotropy. As both regions showed an increase in anisotropy temporally, this could be due to the greater rate of elongation in the OFT region. The pattern between the ventral and dorsal regions remained almost unchanged over this time period. The ventral region had a larger volume of tissue, cell, and ICS, also a larger number of cells. The dorsal region had more elongated cells at the two time points. The only difference in pattern was in the cell density, where the dorsal region had a larger density than the ventral region at Time Point 2 but not at Time Point 3 (when there was no difference between the two regions). The patterns in the OFT region and the dorsal region showed changes between these time points. The dorsal region had a greater tissue and ICS volumes than the OFT region at Time Point 3 which was a reverse from the pattern at Time Point 2. The OFT region had a greater value for cell volume than the dorsal region at Time Point 3, although the cell volume decreased between Time Point 2 and Time Point 3 in both regions. The pattern of cell shape did not change between the two time points.

Time Point 3 to 4 The ventral region and OFT region comparison showed a similar pattern for all parameters except the cell anisotropy. At Time Point 3, the two regions showed no difference in cell shape, whereas at Time Point 4, the OFT region had more elongated cells. Considering the temporal changes, this was due to the decrease of anisotropy in the ventral region. The pattern between the ventral and dorsal regions showed changes in the cell volume and density. The ventral region showed a greater value for the cell volume than the dorsal region at Time Point 3 but there was no difference with the dorsal region at Time Point 4. This may have been related to changes in the density, where there was no difference in density at Time Point 3, but a greater density in the ventral region at Time Point 4. The pattern changes between the OFT region and the dorsal region was the same as the pattern changes between the ventral and dorsal regions. All parameters showed the same patterns except for the cell volume and density. The OFT region showed a larger value for the cell volume and no difference in density at Time Point 3. At Time Point 4, there was no difference in cell volume between these two regions, but the density was higher in the OFT region.

Overall temporal pattern with respect to the spatial changes

OFT The OFT showed an increase in the tissue volume from Time Point 1 to 2. This increase was accompanied by an increase in number and volume of cells and also in the volume of the ICS. The cell shape changed towards increased elongation. From Time Point 2 to Time Point 3, the OFT region showed a decrease in the tissue, cell and ICS volumes. There was a slight increase in the number of cells. Cell density increased in this region and the cells became more spherical in shape. From Time Point 3 to 4 the tissue volume, cell volume and ICS volume increased. The number of cells did not change. The cell density decreased slightly. There was no significant change in cell shape.

Ventral The ventral region had a more consistent spatio-temporal pattern in comparison with the OFT and dorsal regions. Growth seemed to be consistent over time in this region, except from Time Point 2 to 3 in which the average volume of cells decreased. Constant increases in tissue volume between time points were always accompanied by an increase in the number of cells and volume of both the cells and ICS. The pattern of cell shape change was similar to the OFT region, with a slight change from Time Point 3 to 4 to a more spherical shape.

Dorsal In general, smaller changes were observed in the dorsal region. The number of cells did not change in this region. Tissue volume did not change from Time Point 1 to 2. This was accompanied by an increase in the cell volume and a decrease in the ICS volume during this time period. An increase in the tissue volume from Time Point 2 to 3 could be due to an increase

in the ICS volume. From Time Point 3 to 4 the cell volume, as well as the ICS volume, increased and resulted in an increase in the tissue volume and a decrease in the cell density. The cell shape change showed the same pattern of changes as the changes in the OFT region.

Supplementary Figures

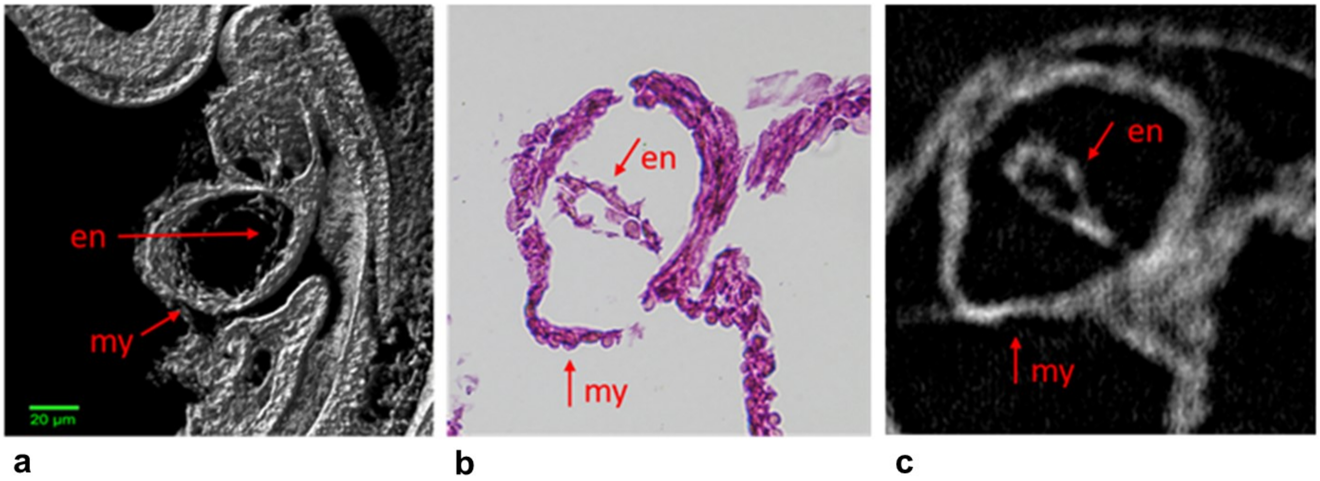

Figure S1 – **Conventional histology confirms single cell membrane of forming endocardium, Related to Figure 2.** (a) 3D micro-CT image of the heart showing endocardium (b) Histology slide (c) Micro-CT corresponding to the area of the histology slide. en: endocardium; my: myocardium.

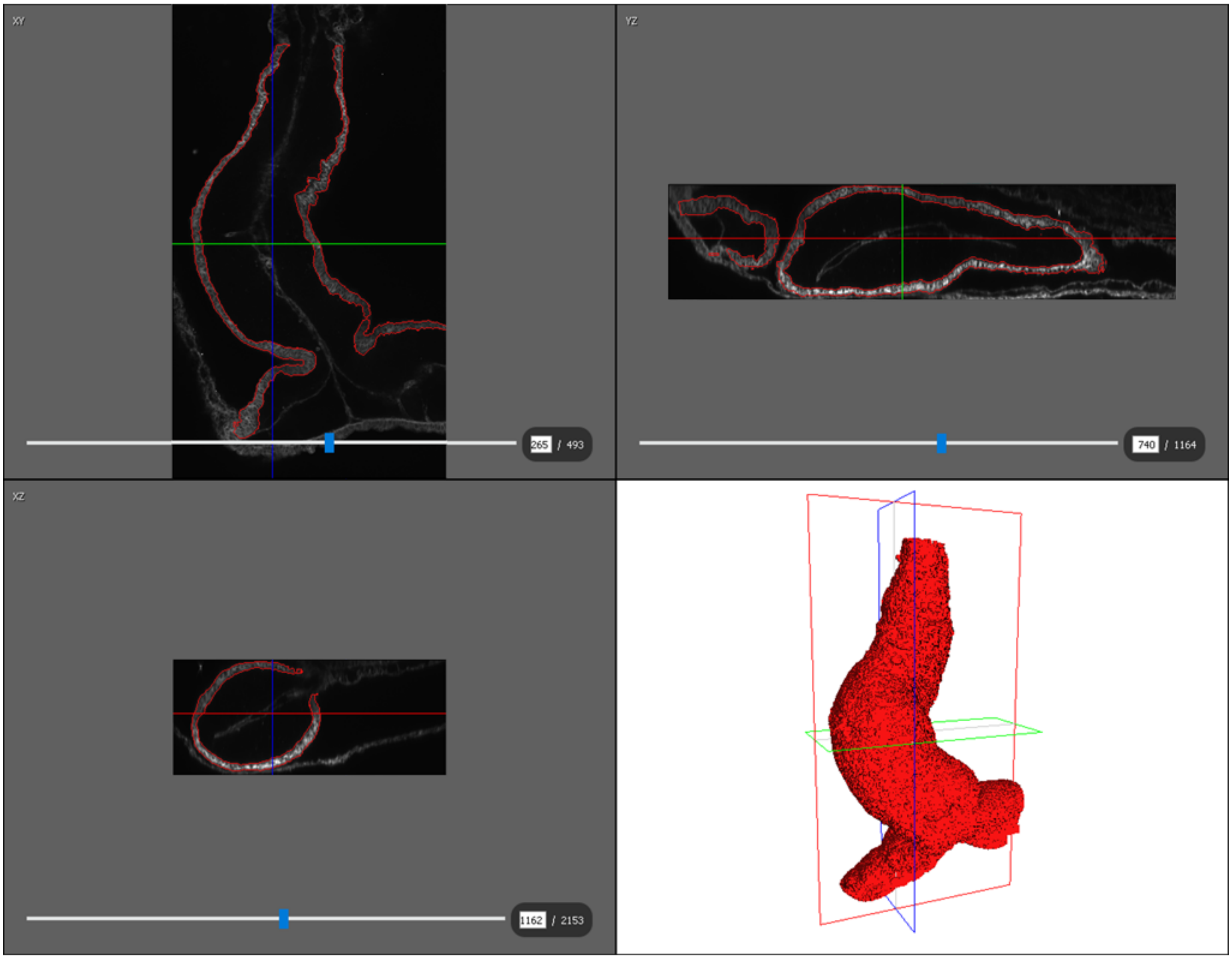

Figure S2 – **3D segmentation of the heart, Related to Figure 3.** 3D confocal image stack of the heart is visualized in three orthogonal planes in Amira's *Segmentation Editor.* The heart area is segmented and viewed from different views resulting in a rendered 3D volume of the heart.

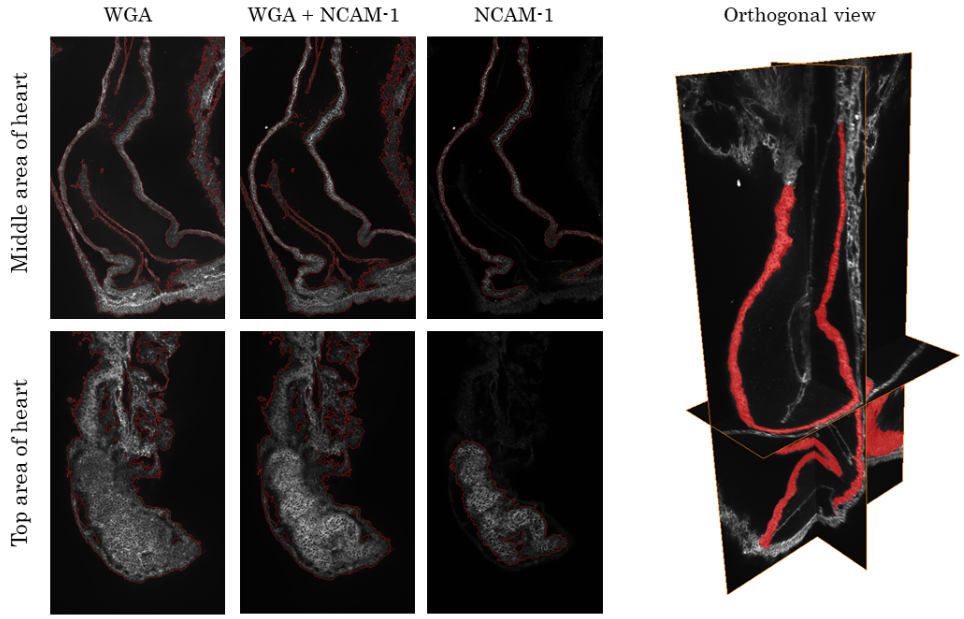

Figure S3 – **Heart area segmentation using NCAM, Related to Figure 3.** Heart area segmentation using a global cell membrane stain (WGA) and a myocardial specific stain (NCAM-1).

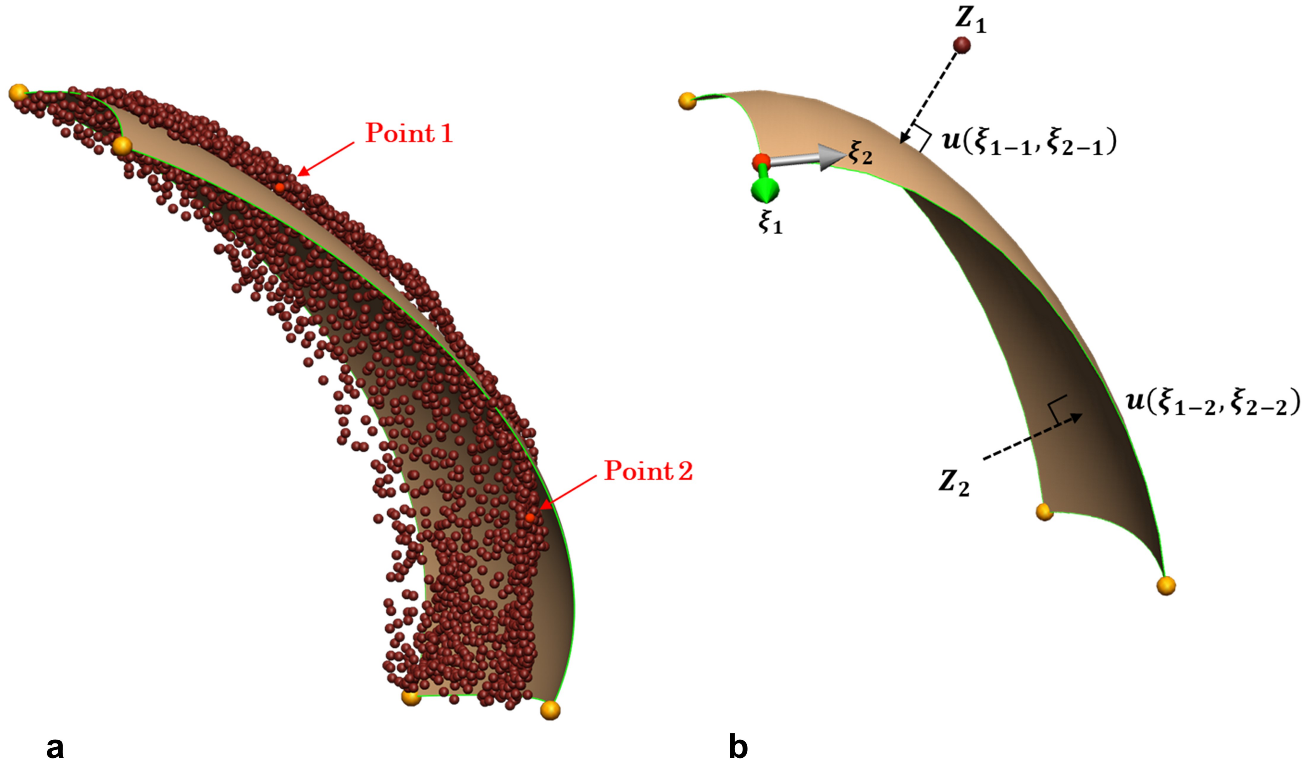

Figure S4 – **Data points projection on the element face, Related to Figure 3.** Projection of the data points on the surface: (a) Data points scattered on the either sides of the surface are projected to the nearest face. (b) Projections of two data points from outer and inner surface (red arrows in (a) as examples) with $\mathbf{z}_d$ coordinates onto the closest element face and resulting nearest point $\mathbf{u}(\xi_{1d}, \xi_{2d})$.

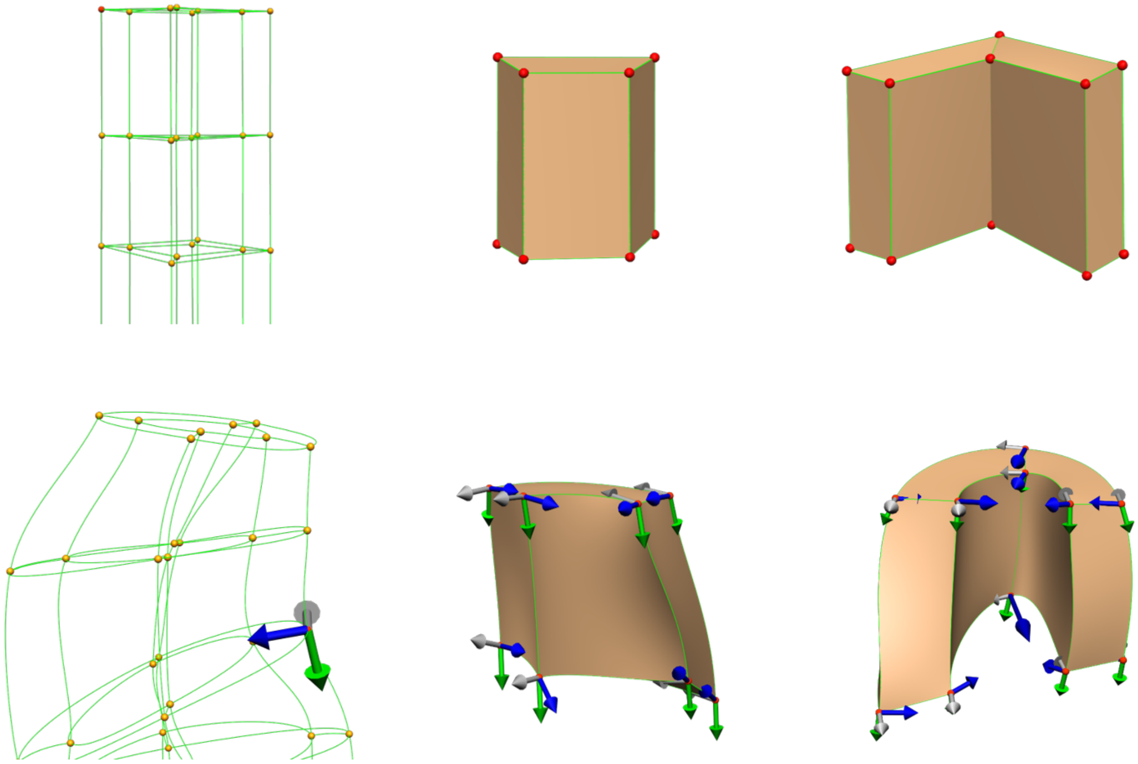

Figure S5 – **An example of mesh deformation in fitting, Related to Figure 3.** An example of how fitting procedure results in a deformation of a linear mesh (above row) into a smoothly shaped mesh (bottom row). The arrows in the smooth elements depict nodal derivatives with respect to the local element ξ coordinates.

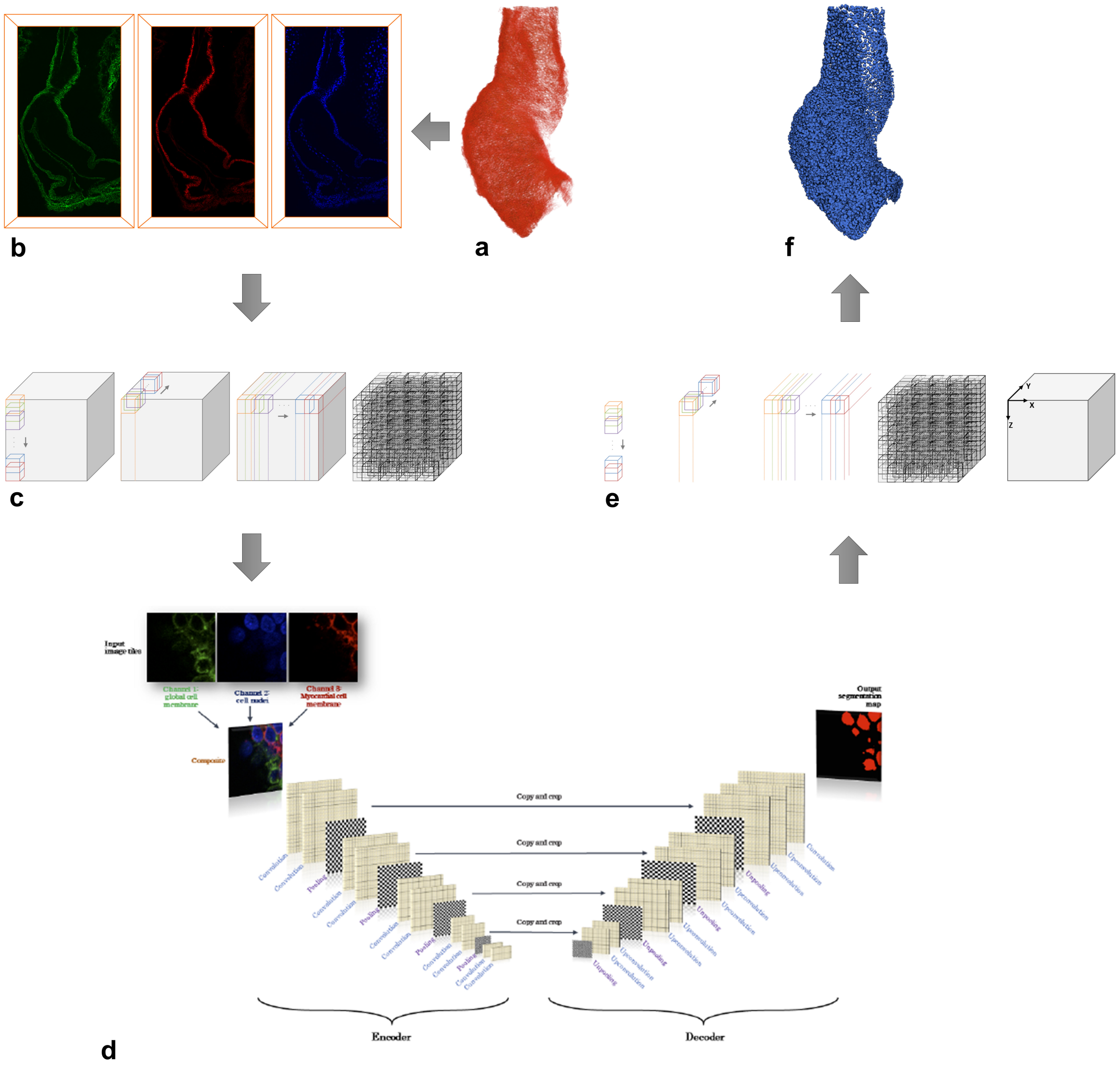

Figure S6 – Automatic segmentation of myocardial cells within entire heart using a Convolutional Neural Netwrok, Related to Figure 4. (a) A 3D reconstructed heart from NCAM-1 channel of confocal image stack. (b) Multichannel images of an entire heart with triple labelling including WGA and NCAM-1 for global and myocardial-specific cell membranes staining (green and red signals, respectively) and DAPI for nuclei staining (blue). (c) The 3D image is uniformly sliced along (from left to right) Z direction, Y direction and then X direction resulting in small slices of the image. (d) The network architecture of a modified U-net model used in this study. (e) The predicted segments are merged in the same order as slicing along (from left to right) Z, Y, and X directions to stack all small segments together with the same overlapping value as slicing, resulting in a $3D$ image tile with the original shape and size. (f) 3D reconstruction of cell segmentation result with individual myocardial cells segmented.

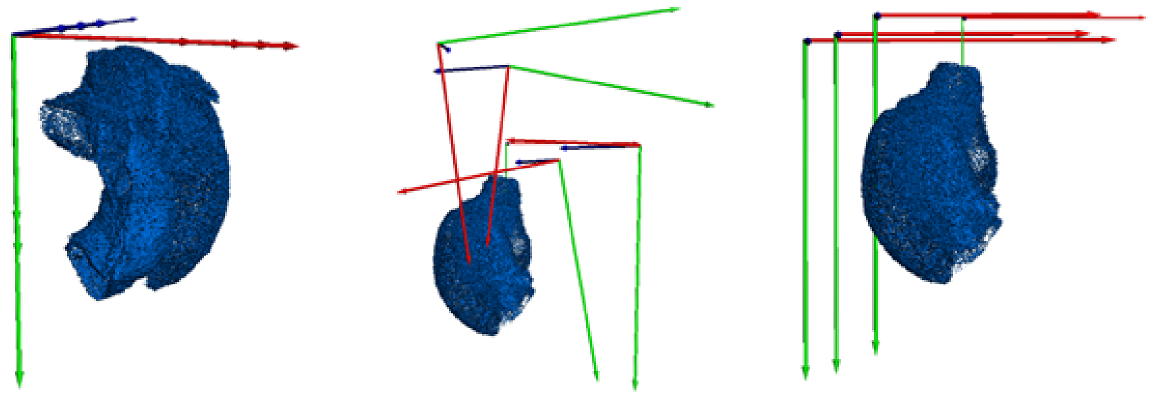

a b c

Figure S7 – **Cell level spatial alignment, Related to Figure 5.** Cell level spatial alignment: (a) At first, the global and local coordinates are aligned for each heart, however the four hearts are not aligned with respect to each other. (b) A rigid geometric transformation was applied to align the four hearts into the global coordinate system, however the local coordinates of the hearts remained unchanged. (c) Alignment of the local coordinates of each heart to the global coordinate resulted in an aligned dataset in which the hearts were spatially aligned at both the tissue and cell level.

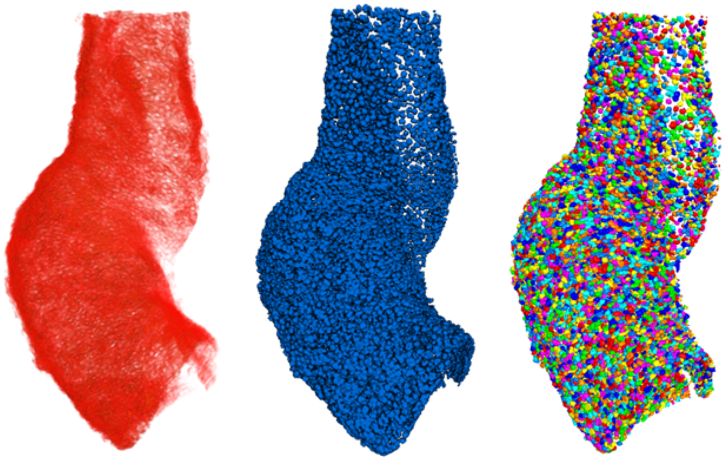

a b c

Volume3d	BaryCenterX	BaryCenterY	BaryCenterZ	Elongation	Anisotropy	index
399.944	409.033	513.36	9.00301	0.242078	0.804579	1
91.9039	424.44	513.066	2.07438	0.655094	0.707017	2
36.5999	435.962	513.652	1.45313	0.826497	0.419159	3
58.6522	429.901	514.732	1.61514	0.888463	0.288654	4
576.362	425.869	520.831	5.01335	0.534631	0.631374	5
36.2535	413.092	520.272	1.9524	0.491271	0.679153	6
173.994	407.913	524.552	2.10285	0.500679	0.690383	7
465.754	411.356	536.216	5.92521	0.642406	0.645523	8
194.43	411.561	544.307	1.71238	0.506051	0.799231	9
309.31	426.098	545.932	2.87673	0.673177	0.775472	10
244.769	368.447	547.629	2.19903	0.313916	0.735562	11
13.6239	361.13	560.709	0.618989	0.79888	0.646456	12
60.8459	363.216	573.191	1.67425	0.348741	0.699458	13
176.072	356.763	591.987	1.54862	0.852276	0.730865	14
297.995	415.887	592.93	6.75072	0.611733	0.585137	15
636.515	404.849	596.314	5.74239	0.695034	0.423032	16
44.7974	377.658	594.745	1.35163	0.651919	0.482998	17
21.3596	334.629	595.53	0.894913	0.610391	0.614221	18
255.738	326.673	599.349	2.48855	0.408278	0.782714	19
426.499	391.974	602.242	4.43372	0.789527	0.400077	20
541.609	371.315	604.962	8.69678	0.853083	0.757785	21
100.448	337.904	606.652	2.07928	0.487093	0.571973	22
35.6763	347.029	609.516	0.499545	0.761934	0.89799	23
178.381	400.986	611.603	2.28498	0.475268	0.654119	24
488.268	378.655	609.631	4.15365	0.840789	0.315453	25
43.4119	394.249	610.414	1.42455	0.482794	0.678887	26
460.328	391.922	621.484	5.69486	0.243773	0.810435	27

d

Figure S8 – 3D single cell analysis within the entire heart, Related to Figure 5. 3D single cell analysis within the entire heart: (a) A 3D confocal image of the heart with labelled myocardial membrane as shown from an frontal view. (b) Segmentation of the individual myocardial cells using the convolutional neural networks method results in a 3D binary image with segmented cells. (c) The cell segmentation result is used for single cell analysis in Amira in which individual cells are labelled (and coloured in image), and measured for different parameters. (d) Label analysis results in a data file listing individual cells with their indexes, coordinates, and their values for measured features.

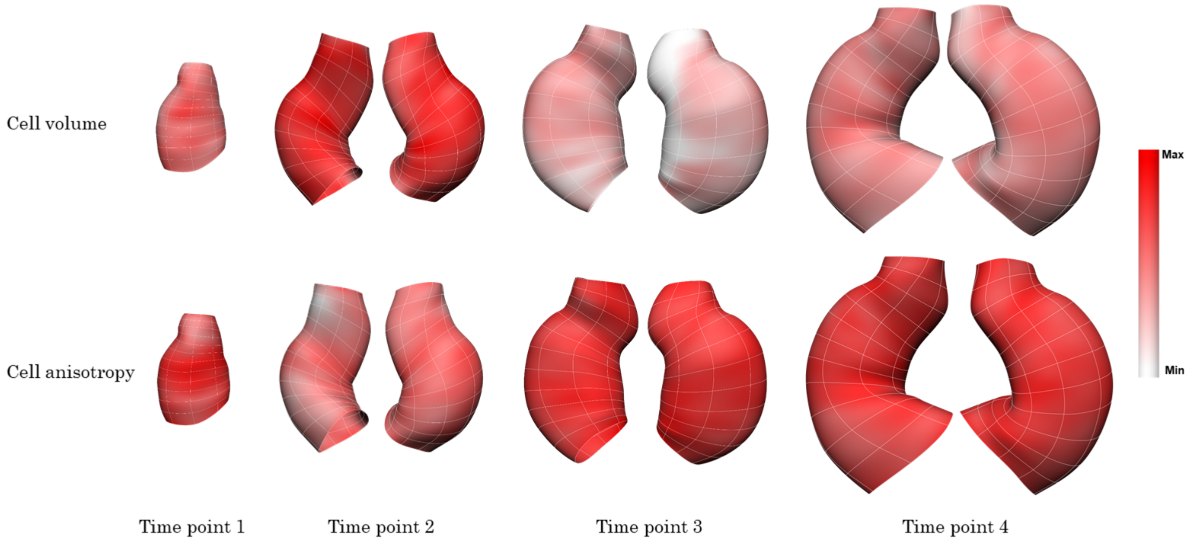

Figure S9 – **Temporal changes in the fitted field, Related to Figure 6.** Temporal changes in the fitted field: The spectrum range is set between the minimum and maximum of values over all samples to visualise temporal changes. (Top row) The temporal pattern from fitting cell volume over the mesh geometries is shown. The white to red spectrum represents the smallest ($7.6\,\mu m^3$) to the largest cells ($355\,\mu m^3$). Time Points 2 and 3 showed the greatest and lowest value for the mean volume of cells, respectively. (Bottom row) Figures show the temporal fitted pattern for the cell anisotropy changes. The white to red shades show cells from a more spherical shape (Min=0.5) to a more elongated shape (Max=0.92). Time Point 2 seemed to, in general, contain more spherical cells than the other three time points. Anatomical view for each time point: anterior view (left) and posterior view (right), except Time Point 1 with an anterior view only.

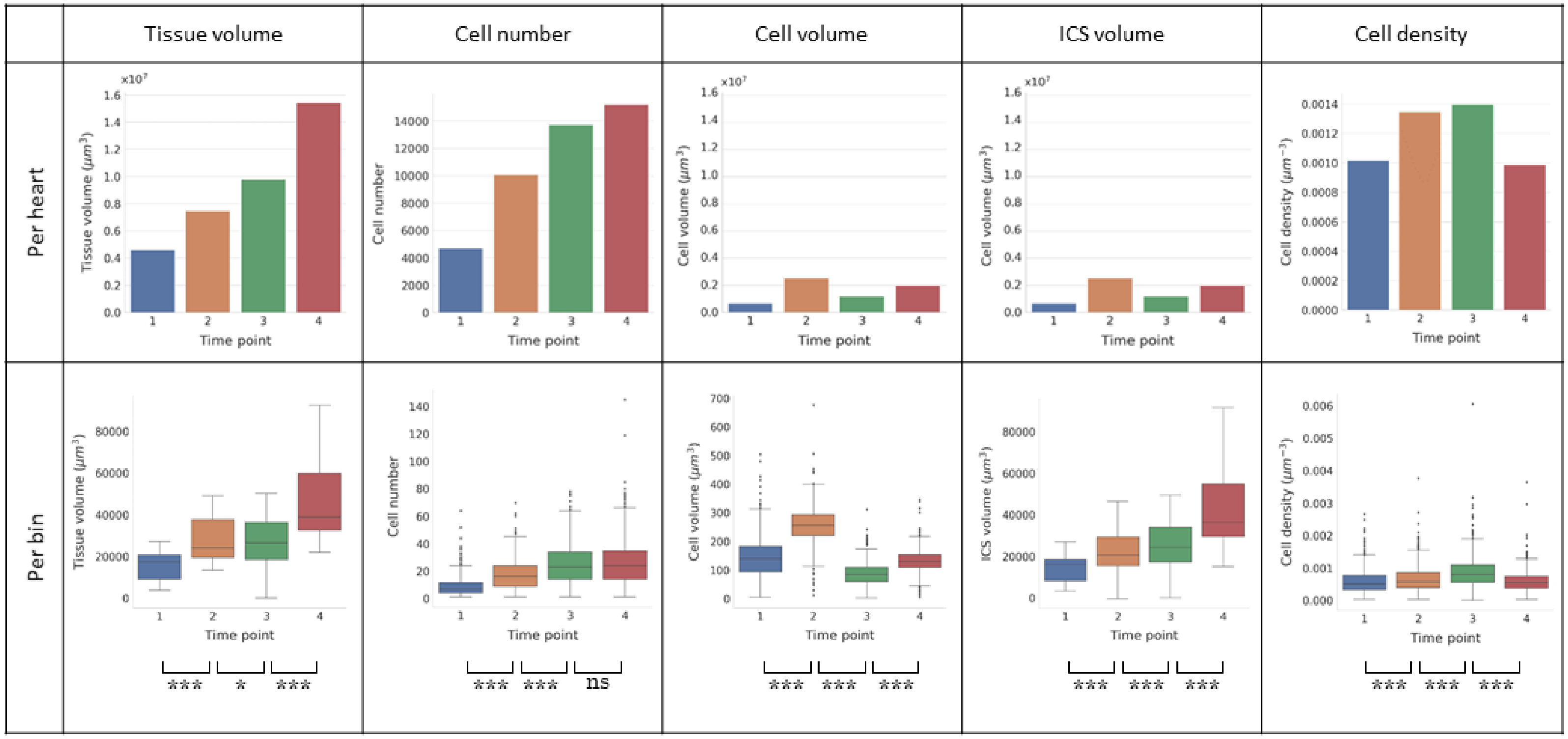

Figure S10 – **Temporal patterns from projected cell data, Related to Figure 6.** Temporal patterns from projected cell data: Cellular values within the entire heart, and in each bin of each time point are plotted. (Top row) Displays bar plots showing total cellular values within each time point. Different cell parameters are presented in columns. (Bottom row) shows box and whisker plots of the cellular values in each bin of the given time points. Distributions are plotted as their median, first, and third quartile (box) and minimum and maximum values (whiskers). The small diamonds show the outliers. Statistical significance for differences between groups are provided below the bars: $p \geq .05$ (ns), $p < .05$ (*), $p < .01$ (**), $p < .001$ (***),. Blue, Time Point 1; orange, Time Point 2; green, Time Point 3; Red, Time Point 4.

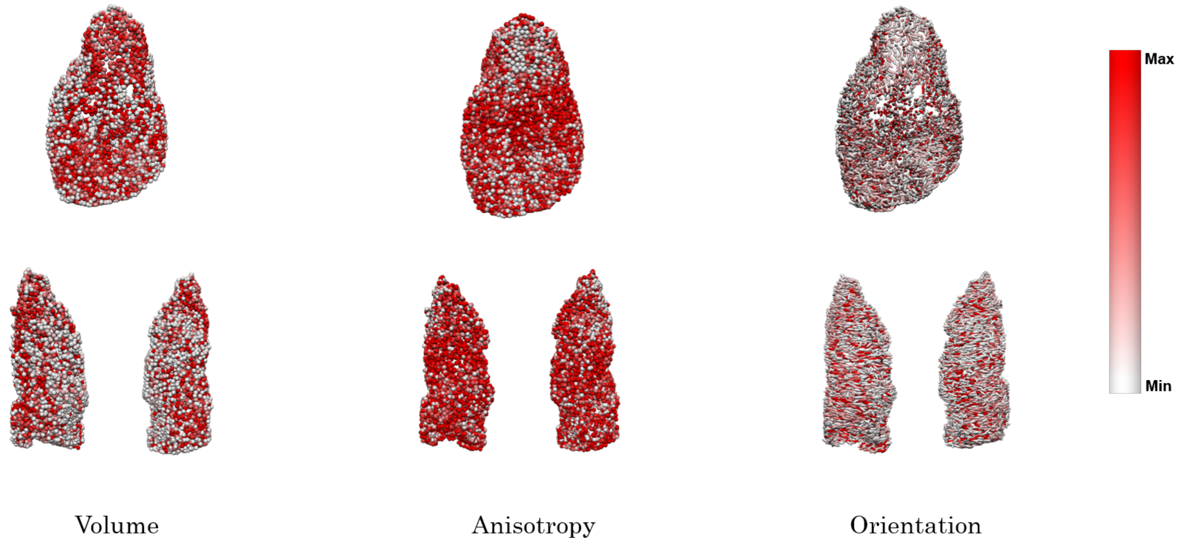

Figure S11 – **Projected cell data at Time Point 1, Related to Figure 6.** Projected cell data at Time Point 1: The values of cell volume, anisotropy, and orientation components (principal eigenvector and eigenvalue) are visualized over the heart geometry. A white to red spectrum shows the minimum to maximum values in the adjusted range for cell volume $(29\,\mu m^3 - 240\,\mu m^3)$, anisotropy $(0.65 - 0.87)$, and principal eigenvalue $(2\,\mu m - 16.6\,\mu m)$. Note that in this figure the spectrum range is set the same as those in Figure 6b for comparison. The variability is much higher and thus the range of values is broader. Top row: anterior view, Bottom: lateral views.

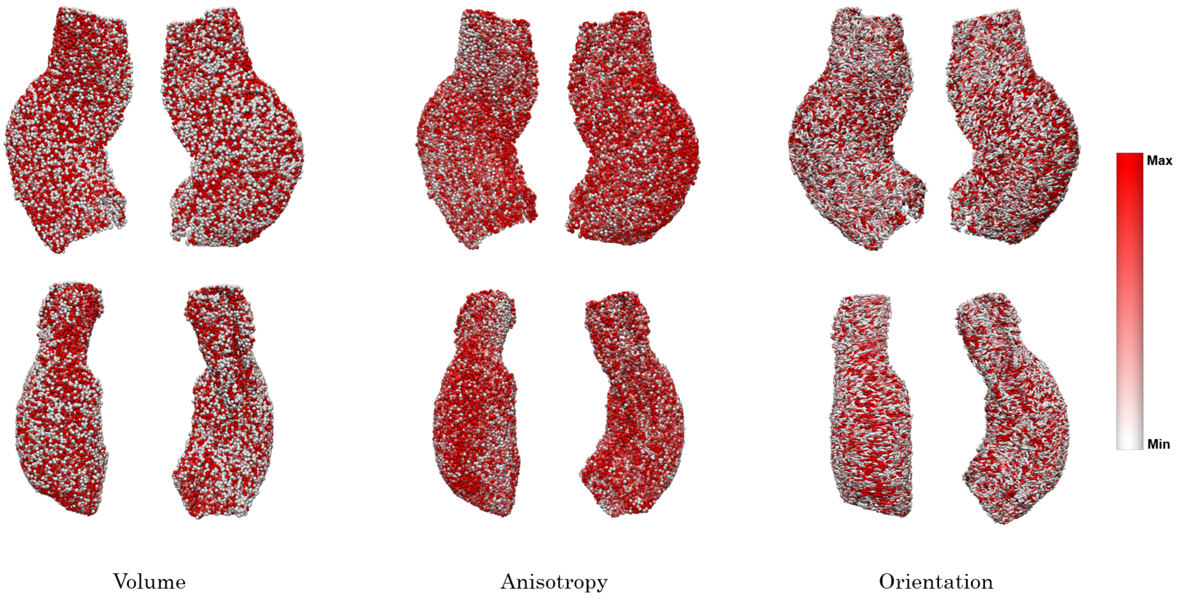

Figure S12 – Projected cell data at Time Point 2, Related to Figure 6. Projected cell data at Time Point 2: The values of cell volume, anisotropy, and orientation components (principal eigenvector and eigenvalue) are visualized over the heart geometry. A white to red spectrum shows the minimum to maximum values in the adjusted range for cell volume $(132\,\mu m^3 - 355\,\mu m^3)$, anisotropy $(0.5 - 0.85)$, and principal eigenvalue $(4.3\,\mu m - 11\,\mu m)$. Note that in this figure the spectrum range is set the same as those in Figure 6c for comparison. The variability is much higher and thus the range of values is broader. Top-left: anterior view, Top-right: posterior view, Bottom-left: ventral view, and Bottom-right: dorsal view.

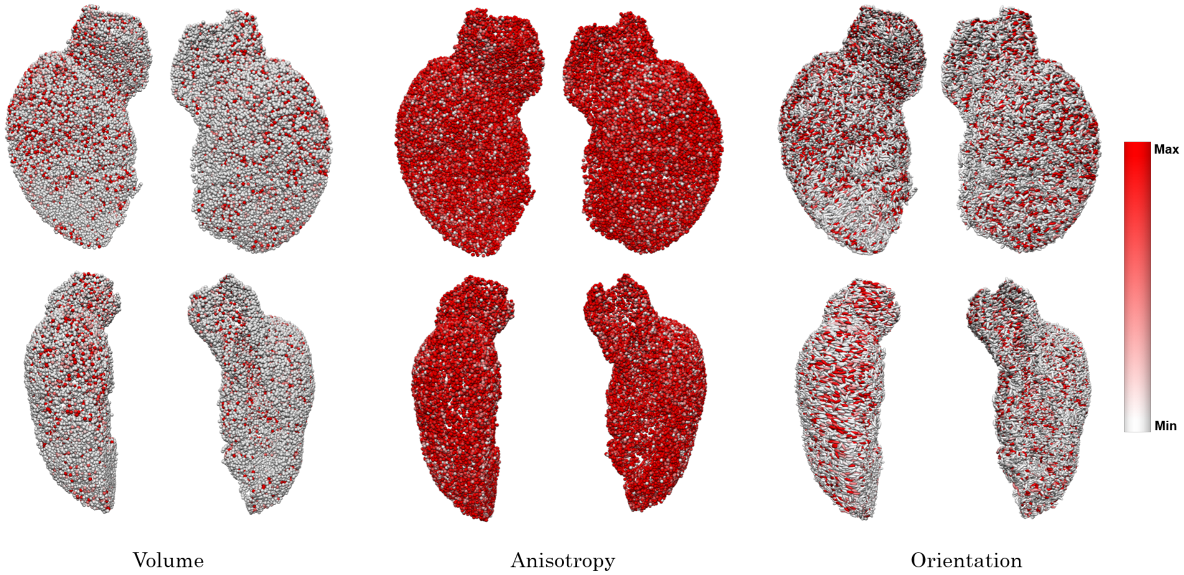

Figure S13 – **Projected cell data at Time Point 3 , Related to Figure 6.** Projected cell data at Time Point 3: The values of cell volume, anisotropy, and orientation components (principal eigenvector and eigenvalue) are visualized over the heart geometry. A white to red spectrum shows the minimum to maximum values in the adjusted range for cell volume $(7.6\,\mu m^3 - 190\,\mu m^3)$, anisotropy $(0.74 - 0.92)$, and principal eigenvalue $(1.2\,\mu m - 16\,\mu m)$. Note that in this figure the spectrum range is set the same as those in Figure 6d for comparison. The variability is much higher and thus the range of values is broader. Top-left: anterior view, Top-right: posterior view, Bottom-left: ventral view, and Bottom-right: dorsal view.

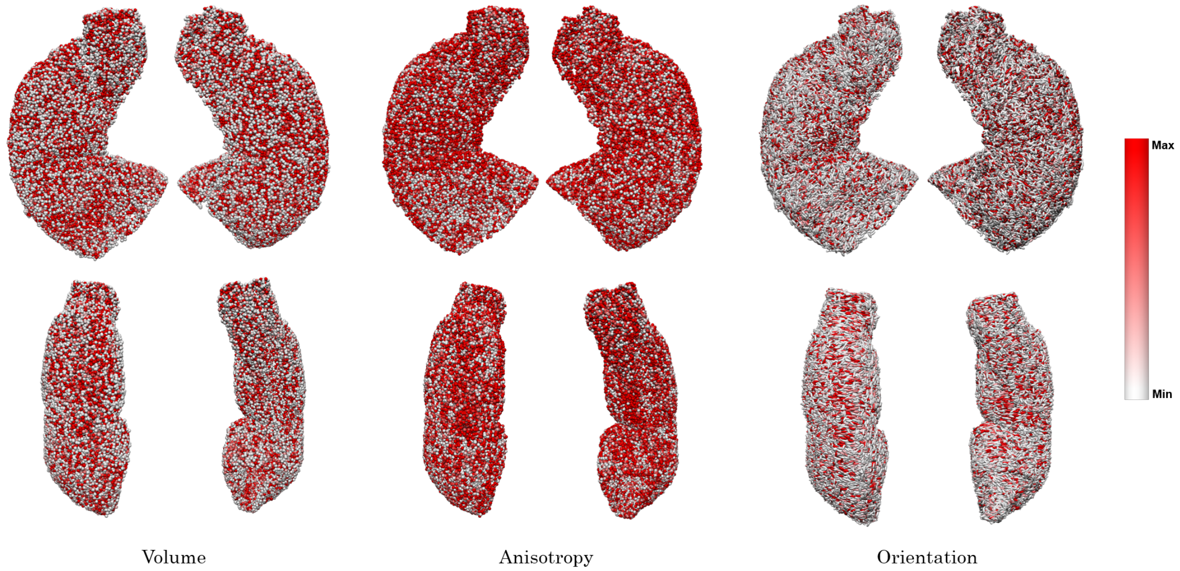

Figure S14 – **Projected cell data at Time Point 4, Related to Figure 6.** Projected cell data at Time Point 4: The values of cell volume, anisotropy, and orientation components (principal eigenvector and eigenvalue) are visualized over the heart geometry. A white to red spectrum shows the minimum to maximum values in the adjusted range for cell volume $(52\,\mu m^3 - 195\,\mu m^3)$, anisotropy $(0.71 - 0.87)$, and principal eigenvalue $(4.2\,\mu m - 14\,\mu m)$. Note that in this figure the spectrum range is set the same as those in Figure 6e for comparison. The variability is much higher and thus the range of values is broader. Top-left: anterior view, Top-right: posterior view, Bottom-left: ventral view, and Bottom-right: dorsal view.

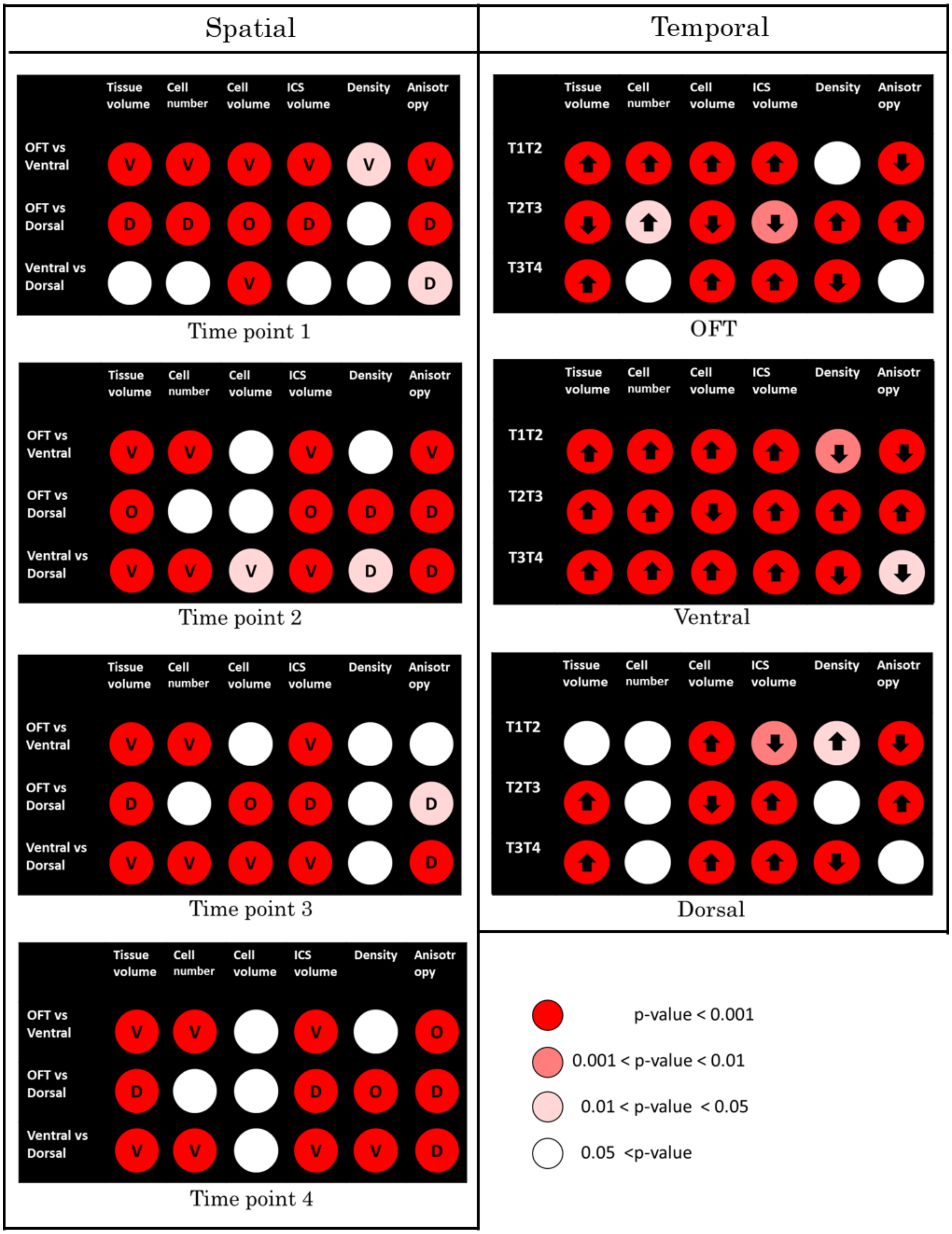

Figure S15 – Regional spatio-temporal pattern from the cell data, Related to Figures 6 and 7. Regional spatio-temporal pattern from the cell data are summarised: The significance level is colour coded. In the spatial column, the comparison result between each two regions at a given time point is shown in the circle using the region initial representing which region had a greater value for the given cell feature. In the temporal column, arrows in the circle indicate whether the change between a given time period for the given parameter is increasing or decreasing. O: OFT region, V: ventral region, D: dorsal region. ↑: increasing , ↓: decreasing.

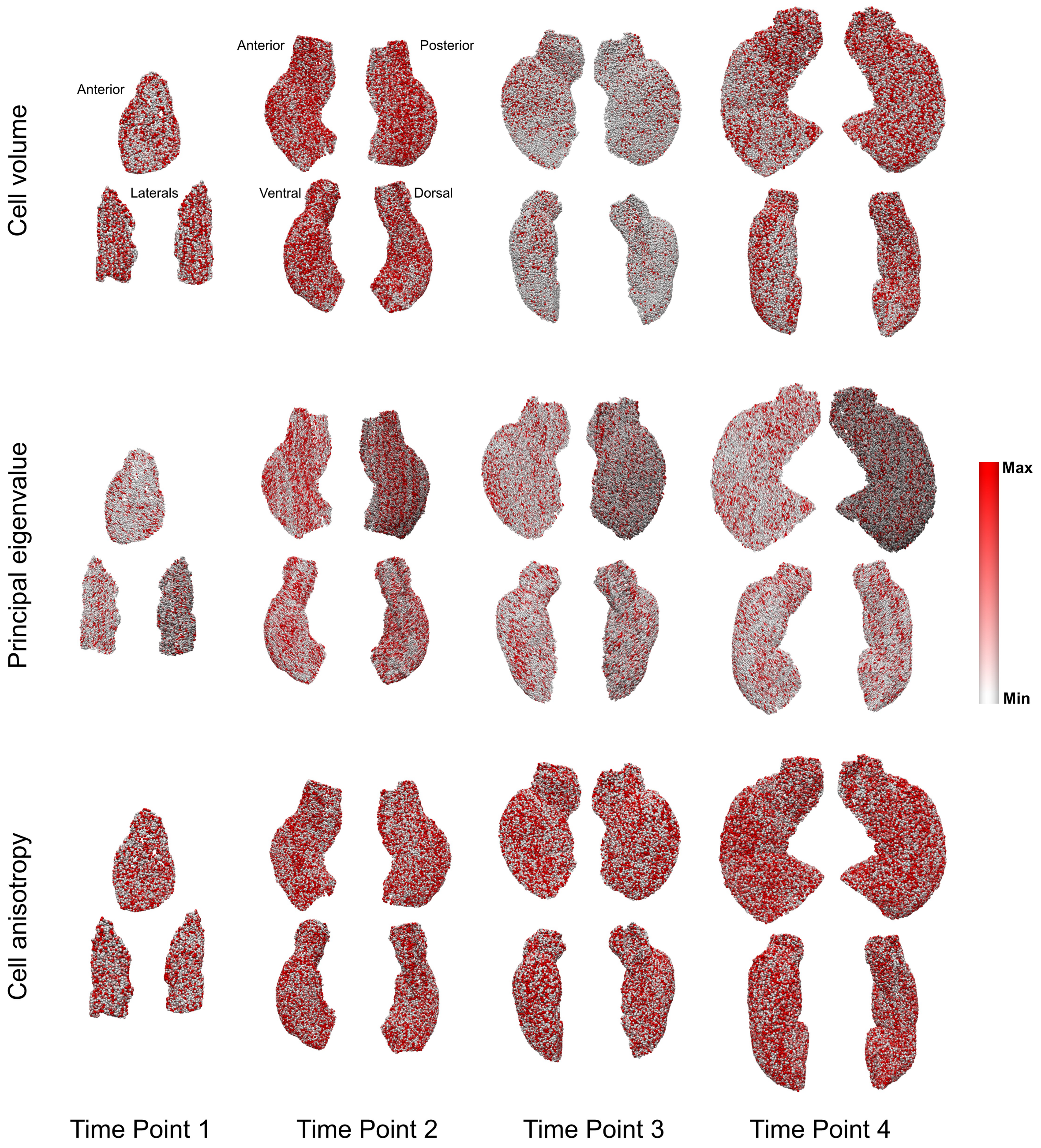

Figure S16 – **Variance analysis from the projected cell data, , Related to Figure 8.** Our variance analysis revealed a similar pattern for the different parameters. In this figure the variance patterns of cell volume, principal eigenvalue, and anisotropy are shown. A white to red spectrum shows the minimum to maximum values in the adjusted range (Time Point 1: $37\,\mu m^2 - 214\,\mu m^2$, Time Point 2: $9\,\mu m^2 - 53\,\mu m^2$, Time Point 3: $19\,\mu m^2 - 200\,\mu m^2$, Time Point 4: $29\,\mu m^2 - 218\,\mu m^2$). Note that in this figure the spectrum range is set the same as those in Figure 8 for comparison. The variability is much higher and thus the range of values is broader.

Supplementary Tables

Measure	Definition
Volume3d	Volume of each separated object
EigenVal1	The largest eigenvalue of the covariance matrix
EigenVal2	The middle eigenvalue of the covariance matrix
EigenVal3	The smallest eigenvalue of the covariance matrix
EigenVec1X	X component of the normalized eigenvector 1
EigenVec1Y	Y component of the normalized eigenvector 1
EigenVec1Z	Z component of the normalized eigenvector 1
EigenVec2X	X component of the normalized eigenvector 2
EigenVec2Y	Y component of the normalized eigenvector 2
EigenVec2Z	Z component of the normalized eigenvector 2
EigenVec3X	X component of the normalized eigenvector 3
EigenVec3Y	Y component of the normalized eigenvector 3
EigenVec3Z	Z component of the normalized eigenvector 3
Anisotropy	1 minus the ratio of the smallest to the largest eigenvalue

Table. S1 – Measures used in Amira for cell feature extraction with their definitions, Related to Figure 5.

Tests of Normality

	Time	Kolmogorov-Smirnov[a]			Shapiro-Wilk		
		Statistic	df	Sig.	Statistic	df	Sig.
Bin volume	1	.185	513	.000	.921	513	.000
	2	.192	575	.000	.903	575	.000
	3	.121	552	.000	.931	552	.000
	4	.231	558	.000	.864	558	.000
Cell number	1	.158	513	.000	.802	513	.000
	2	.089	575	.000	.947	575	.000
	3	.064	552	.000	.960	552	.000
	4	.101	558	.000	.909	558	.000
Cell volume	1	.061	513	.000	.961	513	.000
	2	.055	575	.000	.962	575	.000
	3	.085	552	.000	.875	552	.000
	4	.067	558	.000	.951	558	.000
ICS volume	1	.152	513	.000	.934	513	.000
	2	.117	575	.000	.938	575	.000
	3	.081	552	.000	.950	552	.000
	4	.220	558	.000	.880	558	.000
Cell density	1	.134	513	.000	.887	513	.000
	2	.119	575	.000	.885	575	.000
	3	.100	552	.000	.858	552	.000
	4	.090	558	.000	.860	558	.000

a. Lilliefors Significance Correction

Table. S2 – Normality test of all raw parameters, Related to Figures 6 and 7.

Descriptive statistics

		Statistic								Std. Error		
	Time	Mean	Median	Variance	Std. Deviation	Minimum	Maximum	Skewness	Kurtosis	Mean	Skewness	Kurtosis
BinVolume	1	15468.32184	17420.55860	44182351.50	6646.980630	3803.130096	27148.70054	-.186	-1.233	293.4713640	.108	.215
	2	27145.50638	24171.97270	103752187.5	10185.88177	13517.21880	48998.72826	.661	-.734	424.7806339	.102	.203
	3	28672.44276	26700.84735	137821578.7	11739.74355	11626.50114	50208.31219	.378	-.947	499.6766684	.104	.208
	4	46662.57645	38832.74918	406973388.0	20173.58144	21929.30411	92453.58795	.931	-.303	854.0158015	.103	.206
CellNumber	1	9.16	7.00	64.125	8.008	1	64	2.267	8.159	.354	.108	.215
	2	17.48	16.00	114.518	10.701	1	70	.927	1.310	.446	.102	.203
	3	18.87	18.00	127.294	11.282	1	61	.711	.535	.480	.104	.208
	4	27.26	24.00	315.939	17.775	1	145	1.468	4.518	.752	.103	.206
Volume3d	1	142.2749337	139.2996550	5553.458	74.52152836	4.733770000	503.6260000	.819	2.069	3.290205854	.108	.215
	2	256.5202725	256.3806895	4323.368	65.75232411	11.31480000	675.5370000	.246	3.894	2.742061467	.102	.203
	3	114.8632555	109.7892312	3068.656	55.39544935	7.389290000	621.0470000	2.228	15.083	2.357786902	.104	.208
	4	131.7947778	130.5358587	1784.203	42.23982564	5.311050000	346.5380000	.628	3.632	1.788154407	.103	.206
ICSVolume	1	14179.68848	16233.56846	36965264.85	6079.906648	3347.419166	27087.50794	-.186	-1.193	268.4344361	.108	.215
	2	22753.29283	20611.13094	88825469.01	9424.726469	-418.6374682	46452.79266	.652	-.486	393.0382635	.102	.203
	3	26546.53460	24549.41429	122857447.7	11084.10789	6467.780585	49408.48473	.389	-.873	471.7709615	.104	.208
	4	43079.33531	36512.05970	359229396.0	18953.34788	15190.61599	91395.30335	.923	-.269	802.3591961	.103	.206
Density	1	5.909801E-4	5.156790E-4	.000	3.884548E-4	3.6800E-005	2.6531E-003	1.570	3.801	1.715070E-5	.108	.215
	2	6.739390E-4	5.729630E-4	.000	4.317639E-4	2.2600E-005	3.7730E-003	1.687	5.629	1.800580E-5	.102	.203
	3	6.808473E-4	6.080225E-4	.000	4.133211E-4	3.7300E-005	4.7337E-003	2.513	17.306	1.759211E-5	.104	.208
	4	5.954656E-4	5.562350E-4	.000	3.445916E-4	2.3300E-005	3.6470E-003	2.416	15.045	1.458772E-5	.103	.206

Table. S3 – Descriptive statistics of all parameters for the heart cellular analysis, Related to Figure 6.

	Time Point	N	Mean Rank	Sum of Ranks	Mann-Whitney U	Wilcoxon W	Z	Asymp. Sig. (2-tailed)
	**Ranks**				**Test Statistics**			
Bin volume	1	513	356.22	182742.00	50901.000	182742.000	-18.673	.000
	2	575	712.48	409674.00				
	Total	1088						
	2	575	540.83	310975.00	145375.000	310975.000	-2.440	.015
	3	552	588.14	324653.00				
	Total	1127						
	3	552	401.35	221547.00	68919.000	221547.000	-15.938	.000
	4	558	707.99	395058.00				
	Total	1110						
Cell number	1	513	397.27	203801.00	71960.000	203801.000	-14.610	.000
	2	552	675.85	388615.00				
	Total	1088						
	2	552	476.52	263040.00	110412.000	263040.000	-8.166	.000
	3	575	633.63	353565.00				
	Total	1110						
	3	575	543.86	312722.00	147122.000	312722.000	-2.313	.062
	4	579	584.97	322906.00				
	Total	1127						
Cell volume	1	513	321.08	164714.00	32873.000	164714.000	-22.153	.000
	2	575	743.83	427702				
	Total	1088						
	2	575	813.87	467975.00	15025.000	167653.000	-26.304	.000
	3	552	303.72	167653.00				
	Total	1127						
	3	552	480.28	265112.00	112484.000	265112.000	-7.776	.000
	4	558	629.92	351493.00				
	Total	1110						
ICS volume	1	513	402.19	206325.00	74484.000	206325.000	-14.110	.000
	2	575	671.46	386091.00				
	Total	1088						
	2	575	508.93	292635.00	127035.000	292635.000	-5.797	.000
	3	552	621.36	342993.00				
	Total	1127						
	3	552	408.05	225245.00	72617.000	225245.000	-15.241	.000
	4	558	701.36	391360.00				
	Total	1110						
Cell density	1	513	508.69	260959.00	129118.000	260959.000	-3.550	.000
	2	575	576.45	331457.00				
	Total	1088						
	2	558	522.26	291421.00	152630.000	318230.000	-4.223	.000
	3	575	553.44	318230.00				
	Total	1127						
	3	575	553.44	318230.00	135460.000	291421.000	-3.473	.001
	4	558	522.26	291421.00				
	Total	1110						

Table. S4 – Mann-Whitney U test for temporal changes of all parameters between sequential time points within the heart, Related to Figure 6.

Descriptive statistics[a]

		Statistic								Std. Error		
	Region	Mean	Median	Variance	Std. Deviation	Minimum	Maximum	Skewness	Kurtosis	Mean	Skewness	Kurtosis
Bin volume	Dorsal	19680.97988	17520.96755	21554449.10	4642.676932	9925.848507	27148.70054	-.063	-.785	594.4338690	.306	.604
	OFT	6606.493285	5633.003179	2844412.414	1686.538590	3803.130096	9214.816517	.461	-1.137	153.9592049	.221	.438
	Ventral	19335.90926	19810.97980	6149194.044	2479.756852	12695.40829	22689.32979	-1.618	2.490	253.0891239	.246	.488
Cell number	Dorsal	11.03	9.00	94.932	9.743	1	38	.959	.038	1.248	.306	.604
	OFT	3.64	3.00	5.156	2.271	1	14	1.426	2.892	.207	.221	.438
	Ventral	12.56	11.50	37.533	6.126	3	36	.973	1.618	.625	.246	.488
Cell volume	Dorsal	97.07272950	84.46605394	6845.036	82.73473057	4.733770000	480.3040000	2.686	10.285	10.59309676	.306	.604
	OFT	141.6908936	133.6422637	5860.030	76.55083548	5.830615000	503.6260000	1.302	4.451	6.988103231	.221	.438
	Ventral	175.4696470	169.6400025	3669.846	60.57925075	11.51690750	391.1703875	.385	1.654	6.182843889	.246	.488
ICS volume	Dorsal	18562.63024	17409.78187	17473962.34	4180.186879	9671.148427	27087.50794	-.072	-.580	535.2180855	.306	.604
	OFT	6125.452285	5363.547268	2744275.609	1656.585527	3347.419166	9203.155287	.466	-1.132	151.2248770	.221	.438
	Ventral	17184.01720	17668.47394	5824667.837	2413.434863	8917.286269	21193.92253	-1.379	2.269	246.3201642	.246	.488
Cell density	Dorsal	.0005424190	.0004255220	.000	.0004232018	.000036800	.001575306	.616	-.649	.0000541854	.306	.604
	OFT	.0005769524	.0005292295	.000	.0003982436	.000108521	.002488258	2.055	5.696	.0000363545	.221	.438
	Ventral	.0006462944	.0006278950	.000	.0002996454	.000153507	.001842087	.996	1.843	.0000305824	.246	.488
Cell anisotropy	Dorsal	.8266634476	.8230150000	.003	.0533197165	.667866000	.983145000	.313	1.298	.0068268901	.306	.604
	OFT	.7450267410	.7534798930	.012	.1110682440	.438790500	.956503000	-.501	.046	.0101390971	.221	.438
	Ventral	.8010981748	.8089272800	.002	.0492100054	.686403895	.896681539	-.366	-.640	.0050224751	.246	.488

a. Time Point 1

Table. S5 – Descriptive statistics of all parameters for a regionalised analysis at Time Point 1, Related to Figure 6.

Descriptive statistics[a]

	Region	Statistic								Std. Error		
		Mean	Median	Variance	Std. Deviation	Minimum	Maximum	Skewness	Kurtosis	Mean	Skewness	Kurtosis
Bin volume	Dorsal_P	19501.09063	20241.33138	17307272.64	4160.201034	13517.21880	25502.85256	-.156	-1.204	424.5987400	.246	.488
	OFT	24592.69291	24171.97270	68378376.12	8269.121847	15707.83232	44211.34649	1.150	.824	693.9292903	.203	.404
	Ventral_	36879.78697	38629.11190	74216602.54	8614.905834	22169.29845	48998.72826	-.352	-1.000	879.2551448	.246	.488
Cell number	Dorsal_P	13.02	12.00	53.915	7.343	2	34	.661	.022	.749	.246	.488
	OFT	13.52	10.00	131.698	11.476	1	62	1.448	2.174	.963	.203	.404
	Ventral_	19.03	19.00	44.410	6.664	4	37	.372	.490	.680	.246	.488
Cell volume	Dorsal_P	240.5360199	245.9856768	6509.203	80.67963484	27.82505000	444.2355364	-.096	.262	8.234330749	.246	.488
	OFT	259.2447984	256.6005525	5189.118	72.03553572	11.31480000	675.5370000	1.063	8.012	6.045087871	.203	.404
	Ventral_	266.6421344	262.3272868	2197.179	46.87407848	180.7502000	438.4531889	.717	.903	4.784065602	.246	.488
ICS volume	Dorsal_P	16378.83458	16182.85408	12678489.82	3560.686706	9597.694202	23066.02206	.035	-.894	363.4110651	.246	.488
	OFT	21312.83208	21171.35739	58307269.00	7635.919657	8119.922710	43128.82449	1.084	.681	640.7921429	.203	.404
	Ventral_	31895.40046	33904.00089	69587769.23	8341.928388	14425.84875	46452.79266	-.208	-.946	851.3945009	.246	.488
Cell density	Dorsal_P	.0006583546	.0005918380	.000	.0003372522	.000095100	.001747754	.740	.725	.0000344207	.246	.488
	OFT	.0005404836	.0004381595	.000	.0004473886	.000043605	.002703512	2.001	5.123	.0000375440	.203	.404
	Ventral_	.0005351047	.0005000100	.000	.0002120026	.000095700	.001623867	1.595	6.462	.0000216374	.246	.488
Cell anisotropy	Dorsal_P	.7228193424	.7200055525	.003	.0526790045	.554956750	.858648600	-.233	.877	.0053765284	.246	.488
	OFT	.6451674173	.6636437560	.007	.0817970251	.422882333	.903276000	-.484	.400	.0068642539	.203	.404
	Ventral_	.6899009549	.6929548490	.004	.0632997681	.565229241	.836968636	.001	-.808	.0064605055	.246	.488

a. Time Point 2

Table. S6 – Descriptive statistics of all parameters for a regionalised analysis at Time Point 2, Related to Figure 6.

Descriptive statistics[a]

		Statistic								Std. Error		
	Region	Mean	Median	Variance	Std. Deviation	Minimum	Maximum	Skewness	Kurtosis	Mean	Skewness	Kurtosis
BinVolume	Dorsal	23226.09662	22953.39376	43712313.79	6611.528854	.000000	31961.42666	-.764	.802	758.3943720	.276	.545
	Ventral	19800.86839	18672.56905	41581375.10	6448.362203	11626.50114	29560.13751	.258	-1.367	543.0500617	.204	.406
	Ventral_	42457.56668	43907.04226	48687719.97	6977.658631	28895.89487	50208.31219	-.524	-.920	712.1543019	.246	.488
CellNumber	Dorsal	15.70	15.00	76.721	8.759	2	42	.836	1.196	1.005	.276	.545
	Ventral	16.40	14.00	149.798	12.239	1	76	1.740	4.738	1.031	.204	.406
	Ventral_	30.47	29.00	110.315	10.503	13	64	.840	.695	1.072	.246	.488
Volume3d	Dorsal	56.41608082	49.87358548	1615.884	40.19805583	2.078233333	207.8812800	.943	1.311	4.611033239	.276	.545
	Ventral	96.66264601	93.86711667	2655.016	51.52684966	1.039120000	311.2739750	.842	1.502	4.339343542	.204	.406
	Ventral_	95.52541505	92.59098733	1201.964	34.66935627	32.13393091	217.5562553	.903	1.281	3.538426357	.246	.488
ICSVolume	Dorsal	22276.79779	22622.60746	38762151.75	6225.925775	-5.080140	31816.75826	-.797	.979	714.1626653	.276	.545
	Ventral	18446.86228	17129.47649	38788582.93	6228.048084	9704.246344	28864.27390	.277	-1.363	524.4962658	.204	.406
	Ventral_	39580.09519	40243.57097	46069148.44	6787.425759	25488.27641	49304.16198	-.242	-1.021	692.7387407	.246	.488
Density	Dorsal	.0006612174	.0006178430	.000	.0003271123	.000000000	.001572984	.600	.684	.0000375224	.276	.545
	Ventral	.0008264909	.0007209510	.000	.0005536111	.000043900	.003174363	1.649	3.564	.0000466225	.204	.406
	Ventral_	.0007373636	.0007133500	.000	.0002719264	.000260123	.001764957	.780	1.533	.0000277534	.246	.488
Anisotropy	Dorsal	.8225737456	.8269061665	.002	.0491144673	.614572667	.924950500	-1.022	3.380	.0056338158	.276	.545
	Ventral	.8074175270	.8090683640	.002	.0473266851	.553450000	.947463000	-.923	5.234	.0039856259	.204	.406
	Ventral_	.7982192792	.8011795460	.001	.0312706856	.713920818	.874475500	-.270	.087	.0031915510	.246	.488

a. Time Point 3

Table. S7 – Descriptive statistics of all parameters for a regionalised analysis at Time Point 3, Related to Figure 6.

Descriptive statistics[a]

		Statistic								Std. Error		
	Region	Mean	Median	Variance	Std. Deviation	Minimum	Maximum	Skewness	Kurtosis	Mean	Skewness	Kurtosis
BinVolume	Dorsal	36250.76318	37039.73061	18359868.93	4284.841762	29227.88117	42532.22134	-.369	-1.156	473.1816148	.266	.526
	OFT	30387.01654	27194.43631	51030537.46	7143.566158	21929.30411	39020.48840	.025	-1.887	612.5557445	.208	.413
	Ventral	70812.84053	69657.51562	207230283.5	14395.49525	44858.10332	92453.58795	-.216	-1.023	1469.234082	.246	.488
CellNumber	Dorsal	14.74	14.00	103.057	10.152	1	58	1.678	4.878	1.121	.266	.526
	OFT	17.55	16.00	102.412	10.120	1	65	.873	2.242	.868	.208	.413
	Ventral	41.10	38.50	224.705	14.990	8	77	.476	-.299	1.530	.246	.488
Volume3d	Dorsal	126.0344731	129.1826900	3334.763	57.74740375	11.66120000	316.2675750	.589	1.672	6.377133923	.266	.526
	OFT	129.9614690	127.6565104	2512.110	50.12095005	5.311050000	339.6764600	.581	2.341	4.297836010	.208	.413
	Ventral	132.3568584	129.4343167	584.101	24.16818899	83.55454574	208.6442211	.559	.474	2.466655460	.246	.488
ICSVolume	Dorsal	34321.51316	36328.08805	20256199.10	4500.688736	25476.19887	40873.78771	-.456	-1.253	497.0179255	.266	.526
	OFT	28075.10969	26312.87846	49491140.27	7034.993978	15190.61599	39007.44170	-.003	-1.689	603.2457568	.208	.413
	Ventral	65483.98380	65857.57252	187587076.7	13696.24316	38294.33452	91395.30335	-.299	-.875	1397.866964	.246	.488
Density	Dorsal	.0004077500	.0003629160	.000	.0002782671	.000030800	.001493585	1.507	3.528	.0000307295	.266	.526
	OFT	.0005927674	.0005619515	.000	.0003736122	.000025600	.002964070	2.198	11.107	.0000320370	.208	.413
	Ventral	.0005878051	.0005804280	.000	.0001879261	.000086500	.001070041	.121	.258	.0000191801	.246	.488
Anisotropy	Dorsal	.8252620051	.8255869355	.003	.0557431803	.570111200	.948978000	-1.412	5.773	.0061558045	.266	.526
	OFT	.8040068149	.8050518460	.002	.0424146915	.703105800	.900278800	-.095	-.478	.0036370298	.208	.413
	Ventral	.7827885961	.7850053215	.001	.0379360246	.692005000	.865696143	-.162	-.315	.0038718293	.246	.488

a. Time Point 4

Table. S8 – Descriptive statistics of all parameters for a regionalised analysis at Time Point 4, Related to Figure 6.

	Region	N	Mean Rank	Sum of Ranks	Mann-Whitney U	Wilcoxon W	Z	Asymp. Sig. (2-tailed)	Corrected Sig.
Bin volume	OFT	120	60.50	7260.00	.000	7260.000	-12.656	.000	.000
	Ventral	96	168.50	16176.00					
	Total	216							
	OFT	120.00	60.50	7260.00	.000	7260.000	-11.030	.000	.000
	Dorsal	61.00	151.00	9211.00					
	Total	181.00							
	Ventral	96.00	80.34	7713.00	2799.000	4690.000	-.466	.641	1.000
	Dorsal	61.00	76.89	4690.00					
	Total	157.00							
Cell number	OFT	120	65.91	7909.50	649.500	7909.500	-11.241	.000	.000
	Ventral	96	161.73	15526.50					
	Total	216							
	OFT	120.00	77.28	9273.50	2013.500	9273.500	-4.987	.000	.000
	Dorsal	61.00	117.99	7197.50					
	Total	181.00							
	Ventral	96.00	85.68	8225.50	2286.500	4177.500	-2.313	.021	.062
	Dorsal	61.00	68.48	4177.50					
	Total	157.00							
Cell volume	OFT	120	92.12	11054.00	3794.000	11054.000	-4.307	.000	.000
	Ventral	96	128.98	12382.00					
	Total	216							
	OFT	120.00	104.08	12490.00	2090.000	3981.000	-4.712	.000	.000
	Dorsal	61.00	65.26	3981.00					
	Total	181.00							
	Ventral	96.00	100.52	9650.00	862.000	2753.000	-7.440	.000	.000
	Dorsal	61.00	45.13	2753.00					
	Total	157.00							
ICS volume	OFT	120	60.54	7265.00	5.000	7265.000	-12.609	.000	.000
	Ventral	96	168.45	16171.00					
	Total	216							
	OFT	120.00	60.50	7260.00	.000	7260.000	-10.985	.000	.000
	Dorsal	61.00	151.00	9211.00					
	Total	181.00							
	Ventral	96.00	74.60	7162.00	2506.000	7162.000	-1.520	.129	.387
	Dorsal	61.00	85.92	5241.00					
	Total	157.00							
Cell density	OFT	120	98.35	11802.00	4542.000	11802.000	-2.669	.008	.023
	Ventral	96	121.19	11634.00					
	Total	216							
	OFT	120.00	94.12	11294.00	3286.000	5177.000	-1.123	.262	.786
	Dorsal	61.00	84.87	5177.00					
	Total	181.00							
	Ventral	96.00	85.39	8197.00	2315.000	4206.000	-2.208	.027	.082
	Dorsal	61.00	68.95	4206.00					
	Total	157.00							
Cell anisotropy	OFT	120	93.18	11181.00	3921.000	11181.000	-4.029	.000	.000
	Ventral	96	127.66	12255.00					
	Total	216							
	OFT	120	76.58	9190.00	1930.000	9190.000	-5.192	.000	.000
	Dorsal	61	119.36	7281.00					
	Total	181							
	Ventral	96.00	72.02	6914.00	2258.000	6914.000	-2.413	.016	.047
	Dorsal	61.00	89.98	5489.00					
	Total	157.00							

Ranks[a] / Test Statistics[a]

a. Time Point 1

Table. S9 – Mann-Whitney U test for regional spatial comparisons from the projected cellular data at Time Point 1, Related to Figure 6.

	Ranks[a]				Test Statistics[a]				
	Region	N	Mean Rank	Sum of Ranks	Mann-Whitney U	Wilcoxon W	Z	Asymp. Sig. (2-tailed)	Corrected Sig.
Bin volume	OFT	142	88.70	12595.00	2442.000	12595.000	-8.418	.000	.000
	Ventral	96	165.06	15846.00					
	Total	238							
	OFT	142	138.07	19606.00	4179.00	8835.00	-5.08	.000	.000
	Dorsal	96	92.03	8835.00					
	Total	238							
	Ventral	96	140.84	13521.00	351.00	5007.00	-11.08	.000	.000
	Dorsal	96	52.16	5007.00					
	Total	192							
Cell number	OFT	142	97.96	13911.00	3758.000	13911.000	-5.873	.000	.000
	Ventral	96	151.35	14530.00					
	Total	238							
	OFT	142	115.02	16332.50	6179.50	16332.50	-1.22	.220	.604
	Dorsal	96	126.13	12108.50					
	Total	238							
	Ventral	96	119.40	11462.50	2409.50	7065.50	-5.72	.000	.000
	Dorsal	96	73.60	7065.50					
	Total	192							
Cell volume	OFT	142	115.75	16436.00	6283.000	16436.000	-1.023	.306	.921
	Ventral	96	125.05	12005.00					
	Total	238							
	OFT	142	125.94	17883.00	5902.00	10558.00	-1.75	.079	.238
	Dorsal	96	109.98	10558.00					
	Total	238							
	Ventral	96	106.48	10222.00	3650.00	8306.00	-2.49	.013	.039
	Dorsal	96	86.52	8306.00					
	Total	192							
ICS volume	OFT	142	89.65	12731.00	2578.000	12731.000	-8.133	.000	.000
	Ventral	96	163.65	15710.00					
	Total	238							
	OFT	142	138.37	19649.00	4136.00	8792.00	-5.14	.000	.000
	Dorsal	96	91.58	8792.00					
	Total	238							
	Ventral	96	140.08	13448.00	424.00	5080.00	-10.87	.000	.000
	Dorsal	96	52.92	5080.00					
	Total	192							
Cell density	OFT	142	111.85	15883.00	5730.000	15883.000	-2.084	.037	.112
	Ventral	96	130.81	12558.00					
	Total	238							
	OFT	142	105.75	15017.00	4864.00	15017.00	-3.75	.000	.000
	Dorsal	96	139.83	13424.00					
	Total	238							
	Ventral	96	85.35	8194.00	3538.00	8194.00	-2.78	.005	.020
	Dorsal	96	107.65	10334.00					
	Total	192							
Cell anisotropy	OFT	142	104.96	14904.00	4751.000	14904.000	-3.963	.000	.000
	Ventral	96	141.01	13537.00					
	Total	238							
	OFT	142	90.92	12910.00	2757.00	12910.00	-7.79	.000	.000
	Dorsal	96	161.78	15531.00					
	Total	238							
	Ventral	96	81.79	7852.00	3196.00	7852.00	-3.67	.000	.000
	Dorsal	96	111.21	10676.00					
	Total	192							

a. Time Point 2

Table. S10 – Mann-Whitney U test for regional spatial comparisons from the projected cellular data at Time Point 2, Related to Figure 6.

	Ranks[a]				Test Statistics[a]				
	Region	N	Mean Rank	Sum of Ranks	Mann-Whitney U	Wilcoxon W	Z	Asymp. Sig. (2-tailed)	Corrected Sig.
Bin volume	OFT	141	72.60	10236.00	225.000	10236.000	-12.664	.000	.000
	Ventral	96	187.16	17967.00					
	Total	237							
	OFT	141	97.17	13701.00	3690.00	13701.00	-3.79	.000	.000
	Dorsal	76	130.95	9952.00					
	Total	217							
	Ventral	96	123.09	11817.00	135.00	3061.00	-10.86	.000	.000
	Dorsal	76	40.28	3061.00					
	Total	172							
Cell number	OFT	141	86.77	12235.00	2224.00	12235.00	-8.77	.000	.000
	Ventral	96	166.33	15968.00					
	Total	237							
	OFT	141	107.37	15139.50	5128.50	15139.50	-.52	.603	1.000
	Dorsal	76	112.02	8513.50					
	Total	217							
	Ventral	96	115.20	11059.50	892.50	3818.50	-8.50	.000	.000
	Dorsal	76	50.24	3818.50					
	Total	172							
Cell volume	OFT	141	117.84	16615.00	6604.00	16615.00	-.32	.752	1.000
	Ventral	96	120.71	11588.00					
	Total	237							
	OFT	141	126.99	17906.00	2821.00	5747.00	-5.75	.000	.000
	Dorsal	76	75.62	5747.00					
	Total	217							
	Ventral	96	107.76	10345.00	1607.00	4533.00	-6.29	.000	.000
	Dorsal	76	59.64	4533.00					
	Total	172							
ICS volume	OFT	141	72.16	10175.00	164.00	10175.00	-12.75	.000	.000
	Ventral	96	187.79	18028.00					
	Total	237							
	OFT	141	96.41	13594.00	3583.00	13594.00	-4.02	.000	.000
	Dorsal	76	132.36	10059.00					
	Total	217							
	Ventral	96	122.91	11799.00	153.00	3079.00	-10.78	.000	.000
	Dorsal	76	40.51	3079.00					
	Total	172							
Cell density	OFT	141	118.73	16741.00	6730.00	16741.00	-.07	.942	1.000
	Ventral	96	119.40	11462.00					
	Total	237							
	OFT	141	113.82	16048.00	4679.00	7605.00	-1.54	.124	.372
	Dorsal	76	100.07	7605.00					
	Total	217							
	Ventral	96	92.80	8909.00	3043.00	5969.00	-1.87	.062	.187
	Dorsal	76	78.54	5969.00					
	Total	172							
Cell anisotropy	OFT	141	125.93	17756.00	5791.00	10447.00	-1.89	.059	.059
	Ventral	96	108.82	10447.00					
	Total	237							
	OFT	141	100.82	14216.00	4205.00	14216.00	-2.61	.009	.018
	Dorsal	76	124.17	9437.00					
	Total	217							
	Ventral	96	72.02	6914.00	2258.00	6914.00	-4.29	.000	.000
	Dorsal	76	104.79	7964.00					
	Total	172							

a. Time Point 3

Table. S11 – Mann-Whitney U test for regional spatial comparisons from the projected cellular data at Time Point 3, Related to Figure 6.

	Ranks[a]				Test Statistics[a]				
	Region	N	Mean Rank	Sum of Ranks	Mann-Whitney U	Wilcoxon W	Z	Asymp. Sig. (2-tailed)	Corrected Sig.
Bin volume	OFT	136	68.50	9316.00	.000	9316.000	-13.003	.000	.000
	Ventral	96	184.50	17712.00					
	Total	232							
	OFT	136	88.58	12047.00	2731.00	12047.00	-6.33	.000	.000
	Dorsal	82	144.20	11824.00					
	Total	218							
	Ventral	96	130.50	12528.00	.00	3403.00	-11.52	.000	.000
	Dorsal	82	41.50	3403.00					
	Total	178							
Cell number	OFT	136	76.64	10423.50	1107.50	10423.50	-10.77	.000	.000
	Ventral	96	172.96	16604.50					
	Total	232							
	OFT	136	117.22	15942.00	4526.00	7929.00	-2.33	.020	.060
	Dorsal	82	96.70	7929.00					
	Total	218							
	Ventral	96	125.93	12089.00	439.00	3842.00	-10.21	.000	.000
	Dorsal	82	46.85	3842.00					
	Total	178							
Cell volume	OFT	136	114.31	15546.00	6230.00	15546.00	-.59	.554	1.000
	Ventral	96	119.60	11482.00					
	Total	232							
	OFT	136	111.03	15100.00	5368.00	8771.00	-.46	.645	1.000
	Dorsal	82	106.96	8771.00					
	Total	218							
	Ventral	96	92.19	8850.00	3678.00	7081.00	-.75	.452	1.000
	Dorsal	82	86.35	7081.00					
	Total	178							
ICS volume	OFT	136	68.55	9323.00	7.00	9323.00	-12.95	.000	.000
	Ventral	96	184.43	17705.00					
	Total	232							
	OFT	136	86.49	11763.00	2447.00	11763.00	-6.94	.000	.000
	Dorsal	82	147.66	12108.00					
	Total	218							
	Ventral	96	129.93	12473.00	55.00	3458.00	-11.33	.000	.000
	Dorsal	82	42.17	3458.00					
	Total	178							
Cell density	OFT	136	113.22	15398.00	6082.00	15398.00	-.89	.376	1.000
	Ventral	96	121.15	11630.00					
	Total	232							
	OFT	136	123.67	16819.00	3649.00	7052.00	-4.27	.000	.000
	Dorsal	82	86.00	7052.00					
	Total	218							
	Ventral	96	110.04	10564.00	1964.00	5367.00	-5.76	.000	.000
	Dorsal	82	65.45	5367.00					
	Total	178							
Cell anisotropy	OFT	136	130.35	17727.00	4645.00	9301.00	-3.74	.000	.000
	Ventral	96	96.89	9301.00					
	Total	232							
	OFT	136	97.13	13209.00	3893.00	13209.00	-3.73	.000	.000
	Dorsal	82	130.02	10662.00					
	Total	218							
	Ventral	96	65.66	6303.00	1647.00	6303.00	-6.68	.000	.000
	Dorsal	82	117.41	9628.00					
	Total	178							

a. Time Point 4

Table. S12 – Mann-Whitney U test for regional spatial comparisons from the projected cellular data at Time Point 4, Related to Figure 6.

	Ranks[a]				Test Statistics[a]			
	Time Point	N	Mean Rank	Sum of Ranks	Mann-Whitney U	Wilcoxon W	Z	Asymp. Sig. (2-tailed)
OFT	1	120	60.50	7260.00	.000	7260.000	-13.981	.000
	2	142	191.50	27193.00				
	Total	262						
	2	142	166.30	23615.00	6560.000	16571.000	-5.027	.000
	3	141	117.52	16571.00				
	Total	283						
	3	141	94.15	13275.00	3264.000	13275.000	-9.514	.000
	4	136	185.50	25228.00				
	Total	277						
Ventral	1	96	49.34	4737.00	81.000	4737.000	-11.786	.000
	2	96	143.66	13791.00				
	Total	192						
	2	96	76.72	7365.00	2709.000	7365.000	-4.944	.000
	3	96	116.28	11163.00				
	Total	192						
	3	96	51.31	4926.00	270.000	4926.000	-11.294	.000
	4	96	141.69	13602.00				
	Total	192						
Dorsal	1	61	81.56	4975.00	2772.000	7428.000	-.563	.573
	2	96	77.38	7428.00				
	Total	157						
	2	96	70.50	6768.00	2112.000	6768.000	-4.748	.000
	3	76	106.71	8110.00				
	Total	172						
	3	76	41.46	3151.00	225.000	3151.000	-10.087	.000
	4	82	114.76	9410.00				
	Total	158						

a. Bin volume

Table. S13 – Mann-Whitney U test for the regional temporal pattern of tissue volume from projected cellular data, Related to Figure 7.

	Ranks[a]				Test Statistics[a]			
	Time Point	N	Mean Rank	Sum of Ranks	Mann-Whitney U	Wilcoxon W	Z	Asymp. Sig. (2-tailed)
OFT	1	120	83.55	10026.00	2766.000	10026.000	-9.454	.000
	2	142	172.02	24427.00				
	Total	262						
	2	142	129.20	18347.00	8194.000	18347.000	-2.642	.008
	3	141	154.89	21839.00				
	Total	283						
	3	141	130.96	18466.00	8455.000	18466.000	-1.701	.089
	4	136	147.33	20037.00				
	Total	277	277.00					
Ventral	1	96	69.84	6705.00	2049.000	6705.000	-6.655	.000
	2	96	123.16	11823.00				
	Total	192						
	2	96	64.92	6232.50	1576.500	6232.500	-7.882	.000
	3	96	128.08	12295.50				
	Total	192						
	3	96	75.43	7241.00	2585.000	7241.000	-5.257	.000
	4	96	117.57	11287.00				
	Total	192						
Dorsal	1	61	68.48	4177.50	2286.500	4177.500	-1.847	.065
	2	96	85.68	8225.50				
	Total	157						
	2	96	79.35	7618.00	2962.000	7618.000	-1.547	.122
	3	76	95.53	7260.00				
	Total	172						
	3	76	83.08	6314.00	2844.000	6247.000	-.947	.343
	4	82	76.18	6247.00				
	Total	158						

a. Cell Number

Table. S14 – Mann-Whitney U test for the regional temporal pattern of the number of cells from projected cellular data, Related to Figure 7.

	Ranks[a]				Test Statistics[a]			
	Time Point	N	Mean Rank	Sum of Ranks	Mann-Whitney U	Wilcoxon W	Z	Asymp. Sig. (2-tailed)
OFT	1	120	74.94	8993.00	1733.000	8993.000	-11.106	.000
	2	142	179.30	25460.00				
	Total	262						
	2	142	207.72	29496.00	679.000	10690.000	-13.557	.000
	3	141	75.82	10690.00				
	Total	283						
	3	141	111.44	15713.00	5702.000	15713.000	-5.830	.000
	4	136	167.57	22790.00				
	Total	277						
Ventral	1	96	58.64	5629.00	973.000	5629.000	-9.442	.000
	2	96	134.36	12899.00				
	Total	192						
	2	96	144.24	13847.00	25.000	4681.000	-11.904	.000
	3	96	48.76	4681.00				
	Total	192						
	3	96	65.80	6317.00	1661.000	6317.000	-7.655	.000
	4	96	127.20	12211.00				
	Total	192						
Dorsal	1	61	39.21	2392.00	501.000	2392.000	-8.740	.000
	2	96	104.28	10011.00				
	Total	157						
	2	96	122.39	11749.00	203.000	3129.000	-10.622	.000
	3	76	41.17	3129.00				
	Total	172						
	3	76	50.89	3868.00	942.000	3868.000	-7.566	.000
	4	82	106.01	8693.00				
	Total	158						

a. Cell volume

Table. S15 – Mann-Whitney U test for the regional temporal pattern of cell volume from projected cellular data, Related to Figure 7.

	Ranks[a]				Test Statistics[a]			
	Time Point	N	Mean Rank	Sum of Ranks	Mann-Whitney U	Wilcoxon W	Z	Asymp. Sig. (2-tailed)
OFT	1	120	60.69	7283.00	23.000	7283.000	-13.904	.000
	2	142	191.34	27170.00				
	Total	262						
	2	142	156.07	22162.00	8013.000	18024.000	-2.903	.004
	3	141	127.83	18024.00				
	Total	283						
	3	141	95.96	13531.00	3520.000	13531.000	-9.104	.000
	4	136	183.62	24972.00				
	Total	277						
Ventral	1	96	52.40	5030.00	374.000	5030.000	-10.997	.000
	2	96	140.60	13498.00				
	Total	192						
	2	96	73.22	7029.00	2373.000	7029.000	-5.805	.000
	3	96	119.78	11499.00				
	Total	192						
	3	96	53.23	5110.00	454.000	5110.000	-10.790	.000
	4	96	139.77	13418.00				
	Total	192						
Dorsal	1	61	93.43	5699.00	2048.000	6704.000	-3.169	.002
	2	96	69.83	6704.00				
	Total	157						
	2	96	63.08	6056.00	1400.000	6056.000	-6.931	.000
	3	76	116.08	8822.00				
	Total	172						
	3	76	43.30	3291.00	365.000	3291.000	-9.573	.000
	4	82	113.05	9270.00				
	Total	158						

a. ICS volume

Table. S16 – Mann-Whitney U test for the regional temporal pattern of ICS volume from projected cellular data , Related to Figure 7.

	Ranks[a]				Test Statistics[a]			
	Time Point	N	Mean Rank	Sum of Ranks	Mann-Whitney U	Wilcoxon W	Z	Asymp. Sig. (2-tailed)
OFT	1	120	138.87	16664.00	7636.000	17789.000	-1.447	.148
	2	142	125.27	17789.00				
	Total	262						
	2	142	114.99	16328.00	6175.000	16328.000	-5.573	.000
	3	141	169.21	23858.00				
	Total	283						
	3	141	156.81	22110.00	7077.000	16393.000	-3.767	.000
	4	136	120.54	16393.00				
	Total	277						
Ventral	1	96	107.11	10283.00	3589.000	8245.000	-2.647	.008
	2	96	85.89	8245.00				
	Total	192						
	2	96	73.99	7103.00	2447.000	7103.000	-5.613	.000
	3	96	119.01	11425.00				
	Total	192						
	3	96	112.34	10785.00	3087.000	7743.000	-3.951	.000
	4	96	80.66	7743.00				
	Total	192						
Dorsal	1	61	68.56	4182.00	2291.000	4182.000	-2.294	.022
	2	96	85.64	8221.00				
	Total	157						
	2	96	85.61	8219.00	3563.000	8219.000	-.262	.793
	3	76	87.62	6659.00				
	Total	172						
	3	76	100.01	7601.00	1557.000	4960.000	-5.426	.000
	4	82	60.49	4960.00				
	Total	158						

a. Cell density

Table. S17 – Mann-Whitney U test for the regional temporal pattern of cell density from projected cellular data, Related to Figure 7.

	Time Point	N	Mean Rank	Sum of Ranks	Mann-Whitney U	Wilcoxon W	Z	Asymp. Sig. (2-tailed)
		**Ranks[a]**			**Test Statistics[a]**			
OFT	1	120	170.57	20468.00	3832.000	13985.000	-7.671	.000
	2	142	98.49	13985.00				
	Total	262						
	2	142	75.21	10680.00	527.000	10680.000	-13.777	.000
	3	141	209.26	29506.00				
	Total	283						
	3	141	142.28	20061.00	9126.000	18442.000	-.693	.488
	4	136	135.60	18442.00				
	Total	277						
Ventral	1	96	136.09	13065.00	807.000	5463.000	-9.873	.000
	2	96	56.91	5463.00				
	Total	192						
	2	96	54.10	5194.00	538.000	5194.000	-10.571	.000
	3	96	138.90	13334.00				
	Total	192						
	3	96	108.13	10380.00	3492.000	8148.000	-2.899	.004
	4	96	84.88	8148.00				
	Total	192						
Dorsal	1	61	119.85	7311.00	436.000	5092.000	-8.974	.000
	2	96	53.04	5092.00				
	Total	157						
	2	96	54.33	5216.00	560.000	5216.000	-9.521	.000
	3	76	127.13	9662.00				
	Total	172						
	3	76	77.32	5876.00	2950.000	5876.000	-.578	.563
	4	82	81.52	6685.00				
	Total	158						

a. Cell anisotropy

Table. S18 – Mann-Whitney U test for the regional temporal pattern of cell anisotropy from projected cellular data, Related to Figure 7.

Parameter Estimates

Parameter	B	Std. Error	95% Wald Confidence Interval Lower	Upper	Hypothesis Test Wald Chi-Square	df	Sig.
(Intercept)	-2512.053	1142.5325	-4751.376	-272.731	4.834	1	0.028
[Time=1]	2009.398	1436.0401	-805.189	4823.985	1.958	1	0.162
[Time=2]	1442.724	1634.9428	-1761.705	4647.153	0.779	1	0.378
[Time=3]	0[a]						
[Region_4=Dorsal]	2237.203	1345.9791	-400.868	4875.273	2.763	1	0.096
[Region_4=Lateral]	3954.942	1385.2280	1239.945	6669.940	8.152	1	0.004
[Region_4=OFT]	9016.207	1305.8622	6456.764	11575.650	47.671	1	0.000
[Region_4=Ventral]	0[a]						
transdCellNumber	153.474	21.0692	112.179	194.769	53.061	1	0.000
transdVolume3d	1.919	3.1474	-4.250	8.088	0.372	1	0.542
transdICSVolume	0.707	0.0253	0.658	0.757	782.403	1	0.000
[Time=1] * [Region_4=Dorsal]	-1910.498	1918.5552	-5670.797	1849.801	0.992	1	0.319
[Time=1] * [Region_4=Lateral]	9073.769	1975.2658	5202.319	12945.219	21.102	1	0.000
[Time=1] * [Region_4=OFT]	-5035.323	1841.6818	-8644.953	-1425.693	7.475	1	0.006
[Time=1] * [Region_4=Ventral]	0[a]						
[Time=2] * [Region_4=Dorsal]	1603.406	1866.5865	-2055.036	5261.849	0.738	1	0.390
[Time=2] * [Region_4=Lateral]	-3074.482	1956.9642	-6910.062	761.097	2.468	1	0.116
[Time=2] * [Region_4=OFT]	-10118.236	1787.8600	-13622.378	-6614.095	32.029	1	0.000
[Time=2] * [Region_4=Ventral]	0[a]						
[Time=3] * [Region_4=Dorsal]	0[a]						
[Time=3] * [Region_4=Lateral]	0[a]						
[Time=3] * [Region_4=OFT]	0[a]						
[Time=3] * [Region_4=Ventral]	0[a]						
transdAnisotropy	-22923.278	1978.8606	-26801.773	-19044.782	134.191	1	0.000
(Scale)	112156393.622[b]	3916671.1585	104736676.525	120101735.588			

Dependent Variable: transdV

Model: (Intercept), Time, Region_4, transdCellNumber, transdVolume3d, transdICSVolume, Time * Region_4, transdAnisotropy

a. Set to zero because this parameter is redundant.

b. Maximum likelihood estimate.

Table. S19 – Parameter estimates for the final General Linear Model, Related to Figure 9.
